# Packaging and delivering enzymes by amorphous metal-organic frameworks

**DOI:** 10.1038/s41467-019-13153-x

**Published:** 2019-11-14

**Authors:** Xiaoling Wu, Hua Yue, Yuanyu Zhang, Xiaoyong Gao, Xiaoyang Li, Licheng Wang, Yufei Cao, Miao Hou, Haixia An, Lin Zhang, Sai Li, Jingyuan Ma, He Lin, Yanan Fu, Hongkai Gu, Wenyong Lou, Wei Wei, Richard N. Zare, Jun Ge

**Affiliations:** 1 Key Laboratory of Industrial Biocatalysis, Ministry of Education, Department of Chemical Engineering, Tsinghua University, Beijing, 100084 China; 2 School of Food Science and Engineering, South China University of Technology, Guangzhou, 510640 China; 3 State Key Laboratory of Biochemical Engineering, Institute of Process Engineering, Chinese Academy of Sciences, Beijing, 100190 China; 4 Department of Biochemical Engineering and Key Laboratory of Systems Bioengineering of the Ministry of Education, School of Chemical Engineering and Technology, Tianjin University, Tianjin, 300354 China; 5 Key Laboratory of Protein Science, Ministry of Education, School of Life Sciences, Tsinghua University, Beijing, 100084 China; 6 Tsinghua-Peking Joint Center for Life Sciences, Beijing, 100084 China; 7 Beijing Advanced Innovation Center for Structural Biology, Beijing, 100084 China; 8 Shanghai Synchrotron Radiation Facility, Zhangjiang Lab, Shanghai Advanced Research Institute, Chinese Academy of Sciences, Shanghai, 201204 China; 9 State Key Laboratory of Superhard Materials, Jilin University, Changchun, 130012 China; 10 Department of Chemistry, Fudan University, Jiangwan Campus, Shanghai, 200438 China; 11 Biopharmaceutical and Health Engineering Division, Tsinghua Shenzhen International Graduate School, Shenzhen, China

**Keywords:** Enzymes, Metal-organic frameworks, Biomaterials - proteins

## Abstract

Enzymatic catalysis in living cells enables the in-situ detection of cellular metabolites in single cells, which could contribute to early diagnosis of diseases. In this study, enzyme is packaged in amorphous metal-organic frameworks (MOFs) via a one-pot co-precipitation process under ambient conditions, exhibiting 5–20 times higher apparent activity than when the enzyme is encapsulated in corresponding crystalline MOFs. Molecular simulation and cryo-electron tomography (Cryo-ET) combined with other techniques demonstrate that the mesopores generated in this disordered and fuzzy structure endow the packaged enzyme with high enzyme activity. The highly active glucose oxidase delivered by the amorphous MOF nanoparticles allows the noninvasive and facile measurement of glucose in single living cells, which can be used to distinguish between cancerous and normal cells.

## Abstract (web summary)

For packaging enzymes into metal–organic frameworks (MOFs), crystalline MOFs are usually used. Here, the authors encapsulated enzymes in amorphous MOFs a via one-pot co-precipitation process under ambient condition, which led to higher enzymatic activity than in a corresponding crystalline MOF composite.

## Introduction

Detection of intracellular metabolites is important for biomedical applications, for example, the diagnosis of cancers as well as many other diseases^1,2^. Enzymes, as the catalysts in cells, in principle, can accelerate specific reactions to covert intracellular metabolites to detectable products. This biocatalysis in cells could provide a new method for the precise detection of metabolites in living cells. However, the delivery of enzymes and the retention of enzyme activity in cells remain challenging.

Enzymes usually lose their three-dimensional structure under harsh conditions such as high temperatures, polar organic solvents, pH extremes, and protease interactions, resulting in the serious deactivation of enzymes. Previous efforts have been made to increase enzyme stability for the wide applications in biocatalysis, biosensing, and biomedicine. Recently, as a promising candidate, crystalline metal-organic frameworks (MOFs) have been utilized to protect the encapsulated enzymes inside MOFs under harsh conditions^3^. To date, there are two approaches to incorporate enzymes in MOFs. The most frequently used strategy is the adsorption of enzymes in as-synthesized crystalline MOFs^4–7^, which involves the pre-synthesis of mesoporous MOFs followed by the loading of enzyme molecules in the well-designed mesopores in MOFs that have sizes slightly larger than protein molecules. The other approach proposed by our group and others is a one-step process, in which the enzyme molecules, metal ions, and organic ligands are mixed in solution to readily form the enzyme-crystalline MOF composites^8–13^. However, the possibility of hosting molecules by amorphous MOFs (aMOFs) with disordered and fuzzy structures has not been explored.

Here, we report the in situ packaging of enzymes in aMOFs at ambient conditions. The enzyme-aMOF composites are prepared by a one-step process, simply by mixing enzyme, metal ions, and organic ligands in an aqueous solution. The extended X-ray absorption fine structure (EXAFS) analysis and molecular dynamic (MD) simulations suggest that the formation of amorphous structures is mainly caused by coordination defects between metal ions and organic ligands. The mesopores in aMOFs as observed by cryo-electron tomography (Cryo-ET) allow the encapsulated glucose oxidase (GOx) to display 20 times higher activity than that in crystalline MOFs. The aMOFs provide a reasonable protecting effect for the encapsulated enzyme, displaying higher stability compared with the native counterpart. This high activity and stability of enzyme-aMOF composites allows its application in intracellular biosensing. The delivery of GOx by aMOFs enables noninvasive measurement of glucose in a single living cell, which identifies cancer cells from normal cells. This capability shows great promise in cancer diagnosis and understanding tumor metabolism.

## Results and discussion

### Synthesis of enzyme-incorporated amorphous composites

**Fig. 1:**
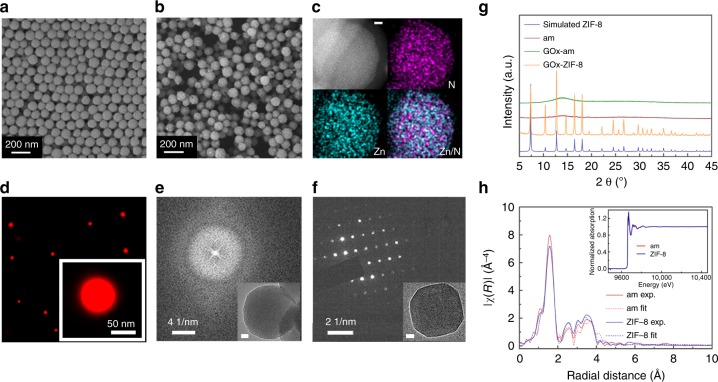
Structural characterizations of enzyme-incorporated composites. Scanning electron microscopy (SEM) images of nanocomposites without (**a**) and with (**b**) GOx enzyme. **c** High-angle annular dark field scanning transmission electron microscopy (HAADF-STEM) image of the GOx-incorporated nanocomposite and energy-dispersive spectrum (EDS) analysis, showing the distribution of Zn and N. Scale bar is 10 nm. **d** Stochastic optical reconstruction microscopy (STORM) image of GOx-incorporated nanocomposites. GOx is labeled with switchable fluorescent dye, Cy5. Inset is a high-resolution image showing the distribution of GOx-Cy5 in a single nanocomposite. **e**, **f** Transmission electron microscopy (TEM) image and selected area electron diffraction (SAED) patterns of GOx-incorporated amorphous nanocomposite (**e**, scale bar in inset is 10 nm) and GOx-incorporated ZIF-8 (**f**, scale bar in inset is 20 nm). **g** X-ray diffraction (XRD) patterns of simulated ZIF-8, amorphous nanocomposite (am), GOx-incorporated amorphous nanocomposite (GOx-am), GOx-incorporated ZIF-8 (GOx-ZIF-8). **h** Pseudoradial distribution functions for ZIF-8, amorphous ZIF from experiment and corresponding fitting data. Data were extracted through Fourier transformation of the X-ray adsorption spectra (inset) obtained at the K edge of zinc. Source data are provided as a Source Data file

Enzyme encapsulation in aMOFs was discovered by accident when decreasing the concentration of 2-methylimidazole (2-MeIM) compared to the previous report^11^ of the in situ incorporation of enzyme in a crystalline zeolitic imidazolate framework-8 (ZIF-8). After mixing 2-MeIM (40 mM), zinc acetate (10 mM), and GOx (0.25 mg mL^−1^) in aqueous solution at room temperature under stirring for 30 min, the product was collected via centrifugation and washed with deionized water. Scanning electron microscopy (SEM) (Fig. 1a, b) and transmission electron microscopy (TEM) images (Supplementary Fig. 1a, b) showed that the composites (with and without enzyme) exhibited the form of nanospheres (~100 nm in diameter). High-angle annular dark field scanning TEM images and energy-dispersive spectrum (EDS) mapping (Fig. 1c) confirmed the distribution of Zn and N (from both 2-MeIM and protein) in nanospheres. The stochastic optical reconstruction microscopy image (Fig. 1d, Supplementary Fig. 2) showed a uniform distribution of GOx in nanocomposites. The characteristic absorption bands at 1640 to 1660 cm^−1^ and 1510 to 1560 cm^−1^ in Fourier transform infrared spectroscopy (FT-IR) (Supplementary Fig. 3, Supplementary Note 1) confirmed again the presence of GOx. Determined by thermogravimetry analysis, the weight percentage of protein in the composites was ~10% (Supplementary Fig. 4, Supplementary Note 2). The selected area electron diffraction (SAED) pattern of the enzyme-MOF nanocomposites (Fig. 1e) suggested a possible amorphous structure, which was markedly different from the SAED pattern of enzyme-ZIF-8 composites (Fig. 1f), which show a clear crystalline structure.

Thus, we examined the crystallinity of enzyme-MOF nanocomposites by X-ray diffraction analysis. Different from pure ZIF-8 and enzyme-ZIF-8 composites, XRD patterns of nanocomposites with/without enzyme (Fig. 1g) implied the existence of amorphous structures. Previously, aMOFs were mostly synthesized via amorphization of crystalline MOFs by high pressure^14,15^, ball milling^16,17^, or heating^18,19^. Here, we highly suspected that, we have prepared aMOFs and their composites with protein in aqueous solution under ambient conditions by just modulating the concentration of organic ligands.

The loss of long-distance order as indicated by XRD patterns makes it difficult to characterize the amorphous composite. We therefore investigated the chemical environment of Zn to determine whether the amorphous structure was still based on the coordination between Zn^2+^ and 2-MeIM. The X-ray photon spectroscopy (Supplementary Figs. 5 and 6) and FT-IR spectra (Supplementary Fig. 7) suggested that a similar coordination between Zn and N was present in amorphous structure as in ZIF-8. Data extracted through Fourier transformation of the X-ray absorption spectra from EXAFS of the amorphous composite was also similar to ZIF-8 (Fig. 1h), exhibiting one strong peak at ca. 1.6 Å for Zn-N coordination^20^, but the number of N coordinated with Zn was decreased in the amorphous structure compared with that in ZIF-8 (Supplementary Fig. 8, Supplementary Table 1). By elemental analysis and inductively coupled plasma optical emission spectrometer (ICP-OES), the molar ratio of N to Zn in ZIF-8 was determined to be 4.0, whereas in the amorphous composites, the ratio of N to Zn dropped to 3.4 (Supplementary Table 2). This result indicates that one zinc ion in the amorphous composites was coordinated with 3.4 nitrogen atoms on average.

### Coordination defects and mesopores in amorphous composites

**Fig. 2:**
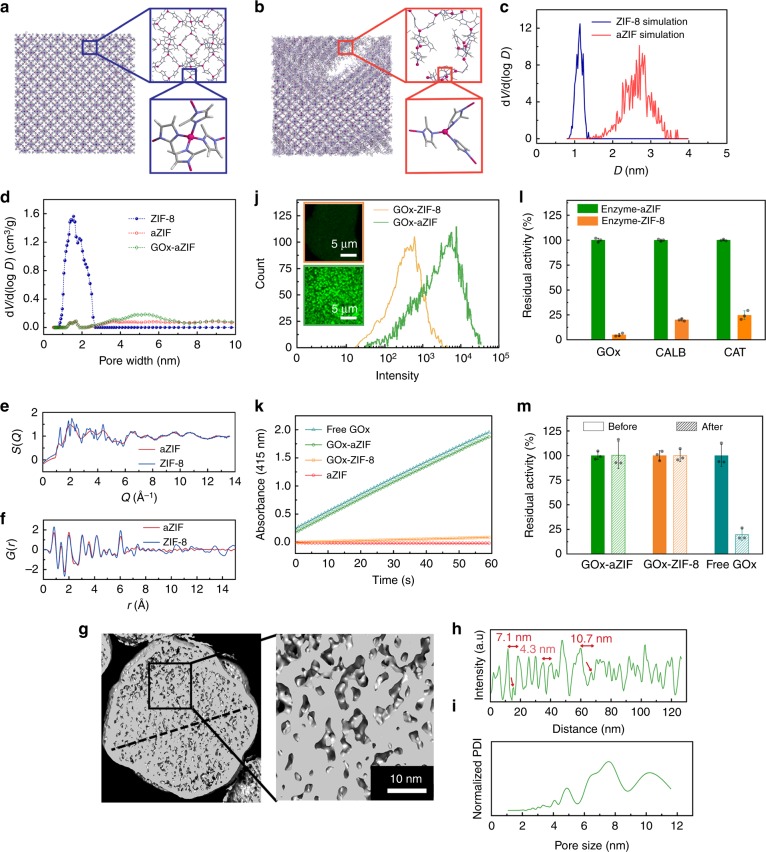
Coordination defects, mesopores, and activities of enzyme-aZIF nanocomposites. Structures of ZIF-8 (**a**) and aZIF (**b**) by molecular simulations (insets: schemes showing coordination). **c** Pore size distribution of ZIF-8 and aZIF by molecular simulations. **d** Density functional theory (DFT) pore size distribution detected with N_2_ adsorption and desorption at 77 K for ZIF-8, amorphous ZIF, and GOx-incorporated amorphous ZIF. X-ray total scattering data (**e**) and PDF (**f**) of aZIF and ZIF-8. **g** Cryo-electron tomography (Cryo-ET) reconstruction and its zoomed image of a single GOx-aZIF nanocomposite. **h** Linear scan of electron density along the dashed line. Three characteristic pore sizes are shown on the plot, tiny peaks possibly representing the encapsulated enzyme molecules are marked by arrowheads. **i** Fast Fourier transformation of the electron density linear scan is converted to pore size distribution of the GOx-aZIF in the left panel. Normalized PDI: normalized pore distribution intensity. **j** Fluorescence intensity of glucose analogs in GOx-ZIF-8 and GOx-aZIF nanocomposites detected via flow cytometry. Inset: confocal laser scanning microscopy (CLSM) showing the fluorescent glucose analog entering the pore of ZIF-8 (up) and amorphous ZIF (down). **k** Enzymatic reaction kinetics for the oxidation of glucose catalyzed by amorphous ZIF, free GOx, GOx-ZIF-8, and GOx-aZIF nanocomposites. **l** Enzymatic activities of enzyme-ZIF-8 and enzyme-incorporated amorphous ZIF, including GOx, *Candida antartic* lipase B (CALB) and catalase (CAT). The activity of corresponding free enzyme was referenced as 100%. Data were represented as mean ± s.d. (*n* = 3). **m** Stability of GOx-aZIF nanocomposites and free GOx against protease digestion under 40 °C for 3 h. Data were represented as mean ± s.d. (*n* = 3). Source data are provided as a Source Data file

Based on the measured ratio of 2-MeIM and Zn^2+^ in the aZIF, MD simulations were utilized to investigate the formation of aZIF. When the ratio between 2-MeIM and Zn^2+^ was 2:1, a perfect structure of ZIF-8 was obtained and stabilized (Fig. 2a), giving an average pore diameter of 1.2 nm (Fig. 2c). When lowering the ratio between 2-MeIM and Zn^2+^ to 1.69:1, the rearrangement of framework occurred (Fig. 2b). Some ordered structure similar to ZIF-8 was preserved. At the same time, the disordered structure emerged in the framework caused by the irregular coordination between 2-MeIM and Zn^2+^ and consequent molecular collapse (Supplementary Fig. 9), leading to the generation of larger pores (Fig. 2b). The appearance of larger pores was further demonstrated by the statistics of the pore size distribution, showing the existence of pores with diameters ranging from 1.5 to 3.5 nm (Fig. 2c), while only micropores of 0.3–1.2 nm can be found in the crystalline ZIF-8. In addition, the result of simulated radial distribution function (Supplementary Fig. 10) also indicated the disappearance of long-distance order in aZIF. A synchrotron radiation X-ray pair distribution function (PDF) experiment was carried out to obtain the total scattering factor, *S*(*Q*) and PDF, *G*(*r*) of both ZIF-8 and aZIF. The experimental results agreed well with the main peaks obtained from simulation (Fig. 2e, f, Supplementary Fig. 11), demonstrating the accuracy of the simulation model. In addition, the XRD spectrum obtained from simulation (Supplementary Fig. 12, Supplementary Note 3) and the simulated atom pair distance distribution (Supplementary Fig. 13) also agreed well with crystallographic structure of ZIF-8, which again proved the model.

The existence of mesopores in aZIF was experimentally proven by Cryo-ET, which provides a three-dimensional landscape of protein-incorporated aZIF nanostructure in the form of thin vitreous hydrated specimens. Imaged by Cryo-ET, the cross-section of GOx-aZIF nanocomposites exhibited the form of spherical particles (~100 nm in diameter) (Fig. 2g, Supplementary Fig. 14) with mesopores plainly distributed inside the particle. Mesopores ranging from ~1 to ~10 nm were clearly observed (Fig. 2g) and measured (Fig. 2h, i). A series of cross-sections of a single GOx-aZIF nanocomposite (Supplementary Fig. 15, Supplementary Movies 1 and 2) suggested that the generated mesopores were interconnected and extended to the surface of the nanocomposite. MD simulations of aZIF without enzyme showed the existence of pores with sizes from 1.5 to 3.5 nm (Fig. 2c). Here, under Cryo-ET, the linear density profile along the dashed line in Fig. 2g demonstrated that the pore size of GOx-aZIF was ~1 to 10 nm by measuring the distance of two neighboring peaks (Fig. 2h) or by its Fourier transformation (Fig. 2i). Interestingly, tiny peaks (arrowheads in Fig. 2h) were often seen in larger mesopores (diameter >8 nm). Because the size of the GOx molecule^21^ is 5.5 nm × 7 nm × 8 nm, it is highly suspected that such tiny peaks represent enzyme molecules located in the mesopores of the GOx-aZIF nanocomposite with a size >8 nm. Other mesopores <8 nm were not occupied by enzyme molecules and were generated mainly during the formation of aZIF, as indicated by MD simulations. A zoomed image of the cross-section of a crystalline ZIF-8 particle was given in Supplementary Fig. 16a, which displayed more regular structure than amorphous ZIFs (aZIF) (Fig. 2g–i). Parallel patterns were observed (red dashed lines), measuring ca. 1.2 nm in distance between the two neighboring dashed lines. This may correspond to the crystallographic planes (1 1 0) of ZIF-8 (Supplementary Fig. 16b), whose interplanar spacing (*d*_1 1 0_) is ca 1.19 nm^22^. Similarly, a linear electron density scan and its fast Fourier transformation were performed along the red dashed line in Supplementary Fig. 16c, d and Supplementary Note 4. Compared with aZIF (Fig. 2g–i), the electron density scan curve of the ZIF-8 sample (Supplementary Fig. 16c, d) showed a more frequent electron density variation, which, in other words, indicate that the pore sizes of crystalline ZIF-8 are generally smaller. Simultaneously, the N_2_ adsorption analysis (Fig. 2d, Supplementary Fig. 17, and Table 3) experimentally supported the existence of mesopores in aZIF and GOx-aZIF. Collapse of micropores to mesopores leads to lower specific surface and thus low nitrogen sorption capacity. Different from ZIF-8, which only has micropores, mesopores from 1 to 4 nm and from 1 to 10 nm were generated in aZIF and GOx-aZIF, respectively, which agrees with the results of MD simulations and Cryo-ET (Fig. 2i). Please note that the different approach utilized in MD simulations (see Supplementary Method section of Molecular simulations for simulation details) resulted in slight difference in pore size distributions of ZIF-8 and aZIF compared with experimental results. From the above investigation, we concluded that although the composites synthesized in this study kept similar coordination and chemical composition as crystalline ZIFs, the low concentration of 2-MeIM in the synthesis resulted in coordination defects in frameworks. The coordination defects led to the loss of long-distance ordering and crystallinity, producing aZIFs and enzyme-aZIF nanocomposites with extra mesopores.

The generation of mesopores facilitated the entering of the substrate glucose into GOx-aZIF nanocomposites. The high affinity of substrate toward enzyme-aZIF composite was proven by using the fluorescent glucose analog 2-deoxy-2-[(7-nitro-2,1,3-benzoxadiazol-4-yl) amino]-d-glucose (2-NBDG). 2-NBDG was incubated with GOx-ZIF-8 or GOx-aZIF nanocomposites under the same conditions. The fluorescence intensity of GOx-aZIF nanocomposites augmented quickly and reached a plateau after 6 min (Supplementary Fig. 18), while the fluorescence intensity of GOx-ZIF-8 was much weaker due to the strong diffusion barrier of micropores in ZIF-8. After incubation for 10 min, the GOx-aZIF nanocomposite captured more fluorescent molecules than GOx-ZIF-8 (Fig. 2j).

### High activity of enzyme packaged in aZIF

The facilitated substrate transportation encouraged us to measure the activity of GOx-aZIF nanocomposites. Surprisingly, the GOx-aZIF nanocomposites exhibited almost ~100% relative activity compared to native GOx at the same protein concentration (Supplementary Fig. 19). This result represents 20 times higher activity than that of GOx-ZIF-8 composites (~5%) (Fig. 2k). Further exploration into the enzymatic kinetics of the GOx-aZIF nanocomposites showed that the Michaelis–Menten (MM) constant, *K*_m_, of the encapsulated GOx was 2.0 mM (Supplementary Fig. 20), whereas for free GOx, *K*_m_ was 2.2 mM (Supplementary Fig. 21), indicating the similar affinity toward substrate glucose (please see Supplementary Table 4 and Supplementary Note 5 for analysis of variance of *K*_m_ values). In contrast, for GOx encapsulated in ZIF-8, *K*_m_ was increased to 12 mM, suggesting a severe limitation for substrate transportation (Supplementary Fig. 22). It is known that ZIF-8 has cavities with windows of ~3.4 Å, which seriously restricted the transport of substrate and therefore significantly reduced enzyme activity after encapsulation^10,11^.

To prove the generality, different enzymes including *Candida Antartic* lipase B (CALB) and catalase (CAT) were encapsulated in aZIFs by the same procedure, producing similar nanospheres with diameters ranging from 80 to 100 nm (Supplementary Figs. 23 and 24). The amorphous structures were proven by the XRD patterns (Supplementary Fig. 25). Similarly, CAT and CALB in aZIF composites exhibited ~5 times higher activity than in corresponding ZIF-8 composites (Fig. 2l). A diffusion-reaction model was established to possibly explain the different activity enhancement factors for GOx, CAT, and CALB in aZIF (Supplementary Table 5, Supplementary Fig. 26, and Supplementary Note 6). A time-dependent evolution of amorphous structures to crystalline structures (Supplementary Figs. 27, 28, Supplementary Table 6, and Supplementary Notes 7, 8) were observed, and the types of enzymes might affect the evolution time. The feeding concentration of ligand also affected the activity of encapsulated enzyme and the structure of obtained composite (Supplementary Figs. 29-31, Supplementary Note 9). This result indicated that the well control of organic ligand concentration and reaction time resulted in the formation of amorphous structures.

The activities of GOx, CAT, and CALB in aZIFs, to the best of our knowledge, showed the highest record of enzymatic activity of enzyme-MOF composites prepared by the one-step co-precipitation process. Previous studies^9,23^ reported <10% activity for enzyme-MOF composites prepared by co-precipitation in solution. The stability of GOx-aZIF composite under mechanic shaking, sonicating, freeze-thawing cycles, reuse, and at high temperature, in solution with different pH was systematically evaluated. Results showed that stability of GOx-aZIF was greatly enhanced compared with free GOx, although slightly lower than that of GOx-ZIF-8 (Supplementary Figs. 32–39 and Supplementary Note 10). Only a small amount of enzyme (6.7–8.0%) was released from aZIF during the activity test (Supplementary Fig. 40 and Supplementary Note 11). Many crystalline MOFs have been used to load enzymes with sufficient protein protection and substrate diffusivity^4–7^. Different from using the pre-synthesized MOFs with mesopores to load enzymes, our research group among others previously developed the one-pot synthesis of enzyme-MOF composites by directly mixing metal ions, organic ligands, and enzymes in solution^8–11^. The advantage of the one-pot synthesis is the simplicity of preparation and the high enzyme loading. However, up to date, most of previous studies of one-pot synthesis focused on using crystalline ZIFs having small pores, which restricted the diffusion of substrates towards the encapsulated enzyme. In this study, following the previous research of one-pot synthesis, we developed the aZIFs to encapsulate enzyme and the larger pores of aZIFs allowed the facilitated substrate diffusion. Compared with the crystalline ZIF-8, the coordination defects in aZIF decreased its stability at harsh conditions, for example, thermal stability and reusability. At the same time, the mesopores inside aZIF, which are also created by the coordination defects endowed the packaged enzyme with a remarkably higher apparent activity compared with enzyme in crystalline ZIF-8.

The GOx-aZIF nanocomposites can be well dispersed in aqueous solution with an average size around 150 nm (Supplementary Figs. 41 and 42) and zeta potential of −23 mV (Supplementary Fig. 43) as determined by dynamic light scattering analysis. Compared with the positive zeta potential of GOx-ZIF-8 (+25 mV), GOx-aZIF with a negative zeta potential could be more biocompatible when incubated with cells. The GOx-aZIF nanocomposites have slight agglomeration in aqueous solution, resulting in sizes larger than that obtained from SEM images (Supplementary Fig. 44). The protective framework prevented the encapsulated enzyme from being attacked by protease. Similar to GOx-ZIF-8, when GOx-aZIF nanocomposites were immersed in trypsin solution, it retained almost 100% of its original activity (Fig. 2m). Further experiment also demonstrated that the GOx was encapsulated inside the particle rather than adsorbed on the surface of aZIF (Supplementary Fig. 45 and Supplementary Note 12). In contrast, under the same condition, free GOx was digested by trypsin, leading to 80% loss of activity. In general, enzymes encapsulated in aZIF outperform those incorporated in other mesoporous materials, such as hydrogels and mesoporous silica, in terms of activity recovery, stability, water dispersibility, and accessibility^24,25^.

### Intracellular glucose detection by enzyme-aZIF

The high activity, dispersibility, and stability of enzyme-aZIF nanocomposites in physiological conditions enabled us to investigate the detection of intracellular metabolites, concentrations of which usually served as important indicators for biochemical processes. For example, glucose metabolism is pivotal to numerous biochemical processes in natural living matters, including energy production, hormonal regulation, and human diseases. The Warburg effect^26^, transition of cell metabolism from oxidative phosphorylation to anaerobic glycolysis resulting in high uptake of glucose, is usually considered as a sign of cancer progression. Several technologies such as Förster resonance energy transfer^27^, nanopipette-based electrochemical sensors^28^, positron emission tomography^29^, and desorption electrospray ionization mass spectrometry imaging^30^ have shown much promise for in situ analysis of glucose in tissues or cells, but still with some limitations (Supplementary Table 7).

**Fig. 3:**
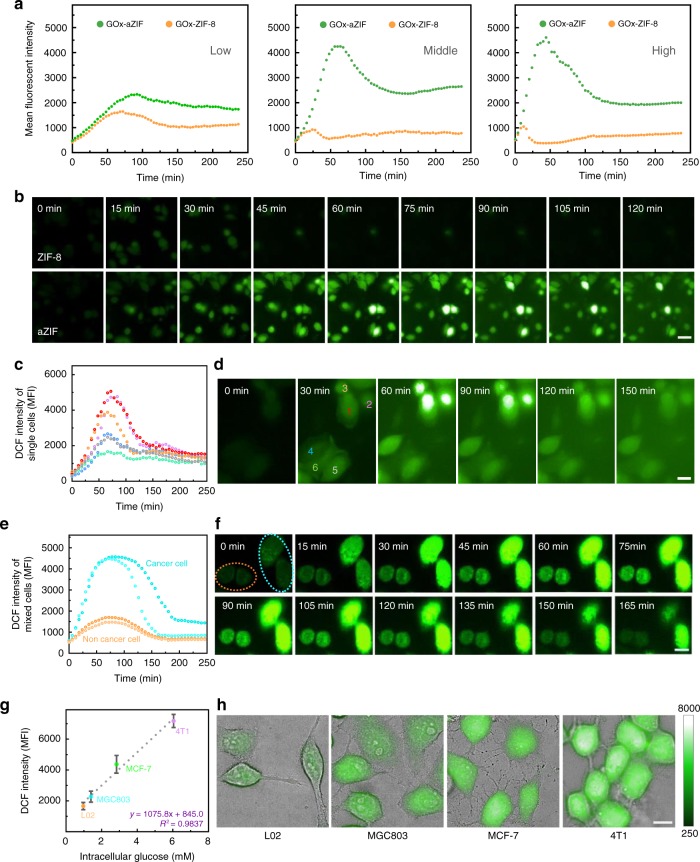
Enzyme delivered into cancerous cells for in situ glucose detection. **a** The kinetics showing the fluorescence intensity of DCFH-DA in MCF-7 cells after delivery of different amounts of GOx-aZIF and GOx-ZIF-8 (concentration of 4.5 μg mL^−1^ (Low), 15 μg mL^−1^ (Moderate), 45 μg mL^−1^ (High)). **b** DCFH-DA cell-staining images in MCF-7 in the presence of GOx-aZIF or GOx-ZIF-8 at moderate concentration of GOx. Scale bar, 30 μm. **c**–**h** Applications of GOx-aZIF for glucose detection in single cells (**c**, **d**), mixed cell types (**e**, **f**), and different cell types (**g**, **h**). DCF intensity kinetics (**c**, **e**). Cell images showing fluorescence changing with time (**d**, **f**). The orange and cyan ovals in (**f**) outline the normal and cancer cells, respectively. **g** Correlation between DCF intensity and intracellular glucose concentration. Data in the calibration plot were presented as mean ± s.d. *n* = 2 biologically independent samples (the images in duplicated samples with a total of ten different fields were captured and calculated). **h** Cell images at the peak intensity of different cell types. Scale bars in **d**, **f**, **h**, 10 μm. Source data are provided as a Source Data file

Enzymes as natural catalysts that responsible for driving metabolic reactions without cell damage, in principle, can be designed as probes for in situ analysis in living cells. For example, GOx catalyzes the oxidation of glucose to d-glucono-1,5-lactone and hydrogen peroxide (H_2_O_2_), which can be identified by the elevated fluorescence of a •OH-sensitive fluorescent dye (2′,7′-dichlorodihydrofluorescein diacetate, DCFH-DA). Considering the acid degradation pathway for free GOx (Supplementary Figs. 46 and 47 and Supplementary Note 13), we concentrated on the comparison of the intracellular activity of the two composites, GOx-aZIF and GOx-ZIF-8. From the kinetic results of fluorescence of DCF (Fig. 3a), a higher enzyme dosage resulted in a faster catalytic speed (represented by a bigger initial slope of the FI–time curve) and a considerable step forward for the maximum peak value of FI. Meanwhile, with the same enzyme dosage, GOx-aZIF displayed a significantly higher activity than GOx-ZIF-8, as indicated by the higher initial slope and the higher peak value of FI. This superiority existed under different enzyme concentrations, especially at a moderate dosage (ca. 15 µg mL^−1^ of nanocomposites), while the peak FI for the GOx-aZIF was four times higher of that for GOx-ZIF-8. At low dosage (4.5 μg mL^−1^ of GOx-aZIF and GOx-ZIF-8), after delivery of enzyme in cells, the majority of GOx-aZIF and GOx-ZIF-8 was possibly deactivated before they can catalyze enough glucose, resulting in slight difference between GOx-aZIF and GOx-ZIF-8 (Fig. 3a, left). At the moderate concentration (15 μg mL^−1^), the difference of enzyme activity between GOx-aZIF and GOx-ZIF-8 was evident and clearly observed (Fig. 3a, middle). This is because the high activity of GOx-aZIF enables it to catalyze the intracellular glucose. In contrast, the activity of GOx-ZIF-8 was still extremely low at moderate dosage. Thus, a huge difference of fluorescent kinetics could be observed between GOx-aZIF and GOx-ZIF-8. At high concentration (45 μg mL^−1^), because both GOx-aZIF and GOx-ZIF-8 were excessive, most of the glucose was consumed very quickly at a similar rate (Fig. 3a, right) giving similar performance. Corresponding images and movies (Fig. 3b, Supplementary Movies 3 and 4) also showed distinguishable cells that rapidly lightened by the GOx-aZIF at a moderate dosage during a continuous detection period (2 h). These results together confirm the feasibility of using GOx-aZIF in dynamic glucose detection.

We investigated further the analysis of glucose in single cells, in mixed cells, and for the discrimination of different cell types to demonstrate its practical application (Supplementary Fig. 48). As shown in Fig. 3c, d, by using GOx-aZIF as the probe, based on the high sensitivity of intracellular fluorescence signal from single-cell level, the active cells (nos. 1–3), which uptake more glucose can be readily distinguished from relatively resting cells (nos. 4–6). Moreover, it can be used to discriminate carcinoma cells (HepG_2_) from normal liver cells (L02) (Fig. 3e, f and Supplementary Movie 5), because HepG_2_ cells uptake more glucose, resulting in higher fluorescence intensity. For further investigation, the intracellular glucose concentrations of four different cell types were firstly measured by a standard chemical lysis method and the glucose concentration increased from the normal tissue cell (L02) to cancer cells (MGC803 < MCF-7 < 4T1). Subsequently, the DCF fluorescence intensity of different cell types was noninvasively detected after delivery of the GOx-aZIF in cells (Fig. 3g, h). GOx-aZIF entering different cells was maintained at the same amount (Supplementary Fig. 49); thus, the peak value of DCF intensity reflects the amount of glucose catalyzed by the GOx-aZIF. It appeared that the peak DCF intensity value accordingly increased with the glucose concentration measured by the standard chemical lysis method (Fig. 3g, Supplementary Fig. 50, and Supplementary Note 14). The linear correlation between the fluorescence intensity and the intracellular glucose concentration can be utilized as a calibration curve. In this sense, the GOx-aZIF not only ensured the absolute quantification of glucose, but also opened up a gentle, non-damage on-live cell detection method that surpassed the chemical lysis method. This method showed good reproducibility (Supplementary Figs. 51–53 and Supplementary Note 15).

In conclusion, we propose that aMOFs can package enzymes with highly retained activity. We studied the chemical structure of aZIF and proved that mesopores (from 1 to 10 nm) were generated in the aZIF during the formation of amorphous structure and incorporation of protein molecules. These mesopores facilitated the substrate transportation and thus greatly improved the activity of encapsulated enzyme, which was demonstrated by different types of enzymes. The delivery of GOx by aZIF allowed the dynamic detection of glucose in a single living cell, which could be used for discriminating different cell types and for distinguishing between normal and cancer cells. This also sheds light on the application in detecting other cellular metabolic processes in a mild and efficient way and promotes the development of new drug delivery systems for medical therapy.

## Methods

### Synthesis of aZIF

In a typical experiment, 1 mL of zinc acetate solution (20 mM) was added into 1 mL of 2-MeIM (80 mM), followed by stirring for 30 min in a 5 mL glass bottle. The synthesized aZIF was then centrifugated (20,000 × *g*, 5 min, ambient temperature), washed for three times with deionized water and lyophilized. Due to the excess amount of 2-MeIM, the yield was calculated based on the conversion of zinc. The product was weighed to be 1.8 ± 0.1 mg with a yield of ca. 41% according to ICP-OES results.

### Synthesis of GOx-aZIF nanocomposites

The synthesis of GOx-aZIF was similar as that of aZIF. After adding zinc acetate solution to 2-MeIM, 80 μL, 0.5–5 mg mL^−1^ GOx was immediately added to the reactor and stirred for 30 min, followed by the same washing and drying procedure. The weight of the product was 2.0 ± 0.1 mg. The yield was calculated to be ca. 45% according to ICP-OES results.

### Enzymatic activity assay of GOx-aZIF nanocomposites

The activity of GOx in aqueous solution was measured by using glucose as the substrate in phosphate-buffered saline (PBS) (pH 7.4). In a typical measurement, 50 μL of GOx-aZIF nanocomposites or free GOx with the same amount of protein was added in PBS containing glucose (100 mM), ABTS (2,2′-azino-bis(3-ethylbenzothiazoline-6-sulfonic acid), 0.28 mg mL^−1^) and horseradish peroxidase (0.05 mg mL^−1^). The increase of absorbance at 415 nm was measured by using a ultraviolet/visible (UV/Vis) spectrophotometer. The MM constant, *K*_m_, was obtained by the non-linear fitting of initial reaction rate with substrate concentration according to the MM equation.

### Enzymatic activity assay of CALB-aZIF nanocomposites

The hydrolytic activity of CALB-ZIF-8 was determined using *p*-nitrophenyl butyrate (*p*-NPB) as the substrate. First, *p*-NPB was dissolved in acetone and then diluted with PBS (50 mM, pH 7.0) containing 1.25% (w/v) Triton X-100, with a final concentration of 0.5 mM. The reaction was initiated by adding 50 µL of enzyme solution (phosphate buffer, 50 mM, pH 7.0) to 950 µL of the substrate solution. The absorbance at 348 nm was recorded using a UV/Vis spectrophotometer.

### Cell culture

The human gastric cancer cell MGC803, human breast cancer cell MCF-7, and hepatic cancer cell HepG_2_ were maintained in Dulbecco’s modified Eagle’s medium medium containing 10% fetal bovine serum (FBS). Mouse breast cancer cell 4T1 and human normal hepatocyte L02 were maintained in RPMI-1640 medium containing 10% FBS. All cells were incubated at 37 °C in an atmosphere of 5% CO_2_. MCF-7 and 4T1 were from the American Type Culture Collection. MGC803 was from the China Infrastructure of Cell Line Resource. L02 was from the Cell bank of Type Culture Collection of the Chinese Academy of Sciences.

### Intracellular detection of glucose

The intracellular glucose was dynamically monitored by using DCFH-DA dye, which could be de-esterified intracellularly to form DCFH. DCFH could react with H_2_O_2_ (the product of glucose conversion catalyzed by GOx) and produce highly fluorescent DCF. Cells were primarily allowed to adhere for 24 h in 96-well plates and be washed for three times with glucose-free PBS prior to the detection. Subsequently, the DCFH-DA dye (at a work concentration of 10 µM) and the GOx-aZIF or GOx-ZIF-8 at certain concentrations were simultaneously added into the cells for dynamic detection (4.5, 15, and 45 µg mL^−1^). The fluorescent images of the cells were recorded during 4-h incubation period (37 °C and 5% CO_2_) via the “Operetta CLS” High Content System (PerkinElmer). Alex Fluor 488 channel (LED power) was selected to acquire the fluorescent signal from DCF, which was excited at 460–490 nm and recorded at 500–550 nm emission wavelength via standard filter sets. Ten percent power was set for the excitation, and 10 ms exposure time was controlled to avoid saturated pixels. Particularly, the instrument was equipped with a 16-bit sCMOS camera, which operated in a fast acquisition mode for exposure time ≤10 ms. The pinhole size was 55 µm.

### Reporting summary

Further information on research design is available in the Nature Research Reporting Summary linked to this article.

## Supplementary information

Supplementary Information

Description of Additional Supplementary Files

Supplementary Movie 1

Supplementary Movie 2

Supplementary Movie 3

Supplementary Movie 4

Supplementary Movie 5

Reporting Summary

Source Data

## Data Availability

Data supporting the findings of this work are available within the paper and its Supplementary Information files. The datasets generated and analyzed during the current study are available from the corresponding author upon request. The source data for Figs. 1g, h, 2c–f, h, i, k–m, 3a, c, e, g and Supplementary Figs. 2–8, 10, 11b, 12b, c, 13, 16c, d, 17, 18a, c, d, 19–22, 25–27, 29, 31, 34–35, 37–38, 40–43, 44b, d, 45, 46, 49, 50, and 52 are provided as a Source Data file.

## Code availability

Codes for the analysis of simulation data are available from the corresponding authors upon request.

## References

[1] Hensley ChristopherT (2016). Metabolic heterogeneity in human lung tumors. Cell.

[2] Carmona-Fontaine Carlos, Deforet Maxime, Akkari Leila, Thompson Craig B., Joyce Johanna A., Xavier Joao B. (2017). Metabolic origins of spatial organization in the tumor microenvironment. Proceedings of the National Academy of Sciences.

[3] Lian X (2017). Enzyme-MOF (metal-organic framework) composites. Chem. Soc. Rev..

[4] Lykourinou V (2011). Immobilization of MP-11 into a mesoporous metal-organic framework, MP-11@ mesoMOF: a new platform for enzymatic catalysis. J. Am. Chem. Soc..

[5] Feng D (2015). Stable metal-organic frameworks containing single-molecule traps for enzyme encapsulation. Nat. Commun..

[6] Li P (2016). Encapsulation of a nerve agent detoxifying enzyme by a mesoporous zirconium metal-organic framework engenders thermal and long-term stability. J. Am. Chem. Soc..

[7] Li P (2018). Hierarchically engineered mesoporous metal-organic frameworks toward cell-free immobilized enzyme systems. Chem.

[8] Lyu F, Zhang Y, Zare RN, Ge J, Liu Z (2014). One-pot synthesis of protein-embedded metal-organic frameworks with enhanced biological activities. Nano Lett..

[9] Wu X, Ge J, Yang C, Hou M, Liu Z (2015). Facile synthesis of multiple enzyme-containing metal-organic frameworks in a biomolecule-friendly environment. Chem. Commun..

[10] Shieh F-K (2015). Imparting functionality to biocatalysts via embedding enzymes into nanoporous materials by a de novo approach: size-selective sheltering of catalase in metal-organic framework microcrystals. J. Am. Chem. Soc..

[11] Liang K (2015). Biomimetic mineralization of metal-organic frameworks as protective coatings for biomacromolecules. Nat. Commun..

[12] Chen T-T, Yi J-T, Zhao Y-Y, Chu X (2018). Biomineralized metal-organic framework nanoparticles enable intracellular delivery and endo-lysosomal release of native active proteins. J. Am. Chem. Soc..

[13] Chen W-H, Vázquez-González M, Zoabi A, Abu-Reziq R, Willner I (2018). Biocatalytic cascades driven by enzymes encapsulated in metal-organic framework nanoparticles. Nat. Catal..

[14] Chapman KW, Halder GJ, Chupas PJ (2009). Pressure-induced amorphization and porosity modification in a metal-organic framework. J. Am. Chem. Soc..

[15] Bennett TD (2011). Reversible pressure-induced amorphization of a zeolitic imidazolate framework (ZIF-4). Chem. Commun..

[16] Bennett TD (2011). Facile mechanosynthesis of amorphous zeolitic imidazolate frameworks. J. Am. Chem. Soc..

[17] Cao S, Bennett TD, Keen DA, Goodwin AL, Cheetham AK (2012). Amorphization of the prototypical zeolitic imidazolate framework ZIF-8 by ball-milling. Chem. Commun..

[18] Nouar F, Eckert J, Eubank JF, Forster P, Eddaoudi M (2009). Zeolite-like metal-organic frameworks (ZMOFs) as hydrogen storage platform: lithium and magnesium ion-exchange and H_2_-(rho-ZMOF) interaction studies. J. Am. Chem. Soc..

[19] Bennett TD (2011). Thermal amorphization of zeolitic imidazolate frameworks. Angew. Chem. Int. Ed..

[20] Goesten M (2013). The molecular pathway to ZIF-7 microrods revealed by in situ time-resolved small- and wide-angle X-ray scattering, quick-scanning extended X-ray absorption spectroscopy, and DFT calculations. Chem. Eur. J..

[21] Hecht HJ, Kalisz HM, Hendle J, Schmid RD, Schomburg D (1993). Crystal structure of glucose oxidase from *Aspergillus niger* refined at 2·3 Å reslution. J. Mol. Biol..

[22] Morris W (2012). NMR and X-ray study revealing the rigidity of zeolitic imidazolate frameworks. J. Phys. Chem. C.

[23] Wu X, Yang C, Ge J (2017). Green synthesis of enzyme/metal-organic framework composites with high stability in protein denaturing solvents. Bioresour. Bioproc..

[24] Yuan J, Wen D, Gaponik N, Eychmüller A (2013). Enzyme-encapsulating quantum dot hydrogels and xerogels as biosensors: multifunctional platforms for both biocatalysis and fluorescent probing. Angew. Chem. Int. Ed..

[25] Fan J (2003). Cubic mesoporous silica with large controllable entrance sizes and advanced adsorption properties. Angew. Chem. Int. Ed..

[26] Katz-Brull R, Seger D, Rivenson-Segal D, Rushkin E, Degani H (2002). Metabolic markers of breast cancer: enhanced choline metabolism and reduced choline-ether-phospholipid synthesis. Cancer Res..

[27] Coverdale JPC (2018). Asymmetric transfer hydrogenation by synthetic catalysts in cancer cells. Nat. Chem..

[28] Nascimento RAS (2016). Single cell “glucose nanosensor” verifies elevated glucose levels in individual cancer cells. Nano Lett..

[29] Gambhir SS (2002). Molecular imaging of cancer with positron emission tomography. Nat. Rev. Cancer.

[30] Banerjee S (2017). Diagnosis of prostate cancer by desorption electrospray ionization mass spectrometric imaging of small metabolites and.ipids. Proc. Natl. Acad. Sci. USA.

