# Supplementary material for: Packaging and delivering enzymes by amorphous metal-organic frameworks
Source: Nat Commun. 2019 Nov 14;10:5165. doi: 10.1038/s41467-019-13153-x (PMC6856190; doi:10.1038/s41467-019-13153-x)
Supplement: Supplementary file 1 — Supplementary Information [file 41467_2019_13153_MOESM1_ESM.pdf]

Supplementary information for

Packaging and delivering enzymes by amorphous metal-organic frameworks

Xiaoling Wu et al.

Supplementary Figures

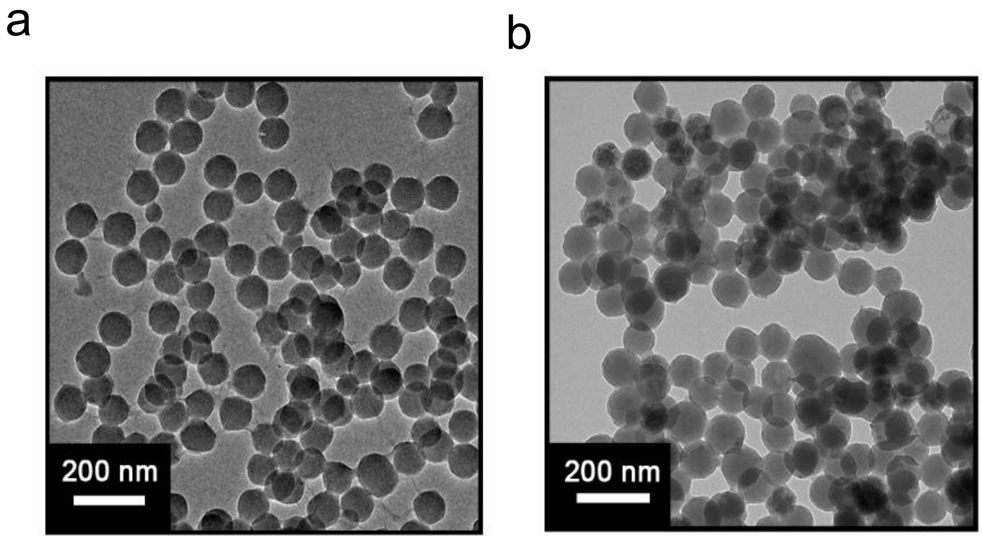

Supplementary Figure 1. Transmission electron microscopy (TEM) images of nanocomposites. Nanocomposites without (a) and with (b) enzyme exhibited the form of nanospheres (~100 nm in diameter).

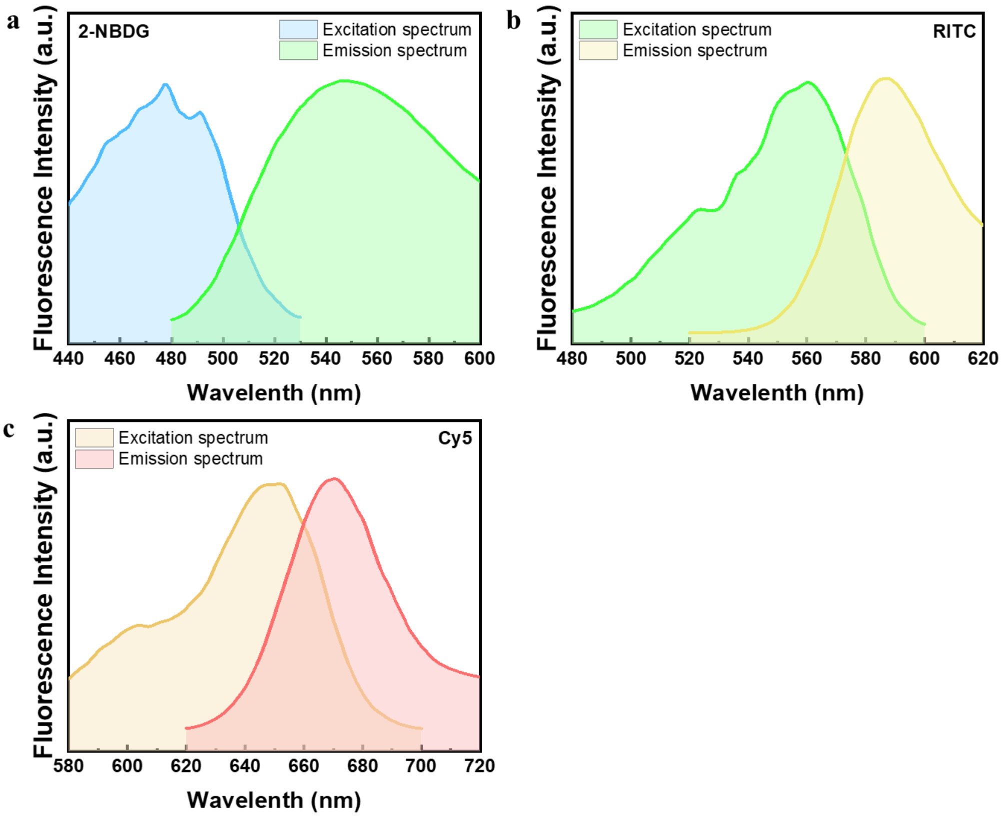

Supplementary Figure 2. The excitation/emission spectra of the dyes used in this study. (a) RITC, (b) 2-NBDG and (c) Cy5. Source data are provided as a Source Data file.

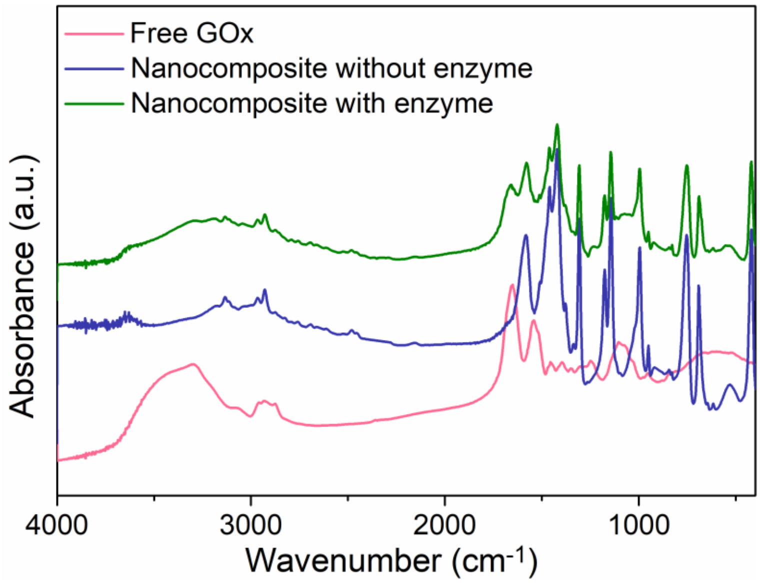

Supplementary Figure 3. FTIR spectra. FT-IR spectra of free GOx, nanocomposites with/without enzymes. The “nanocomposite without enzyme” refers to nanocomposite which was synthesized without adding enzyme. Source data are provided as a Source Data file.

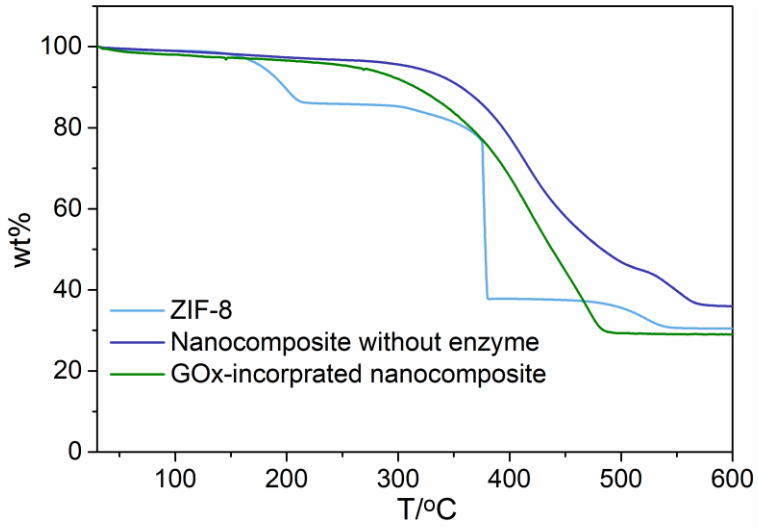

Supplementary Figure 4. TGA curves. TGA curves of ZIF-8, am and GOx-am nanocomposites. The "nanocomposite without enzyme" refers to nanocomposite which was synthesized without adding enzyme. Source data are provided as a Source Data file.

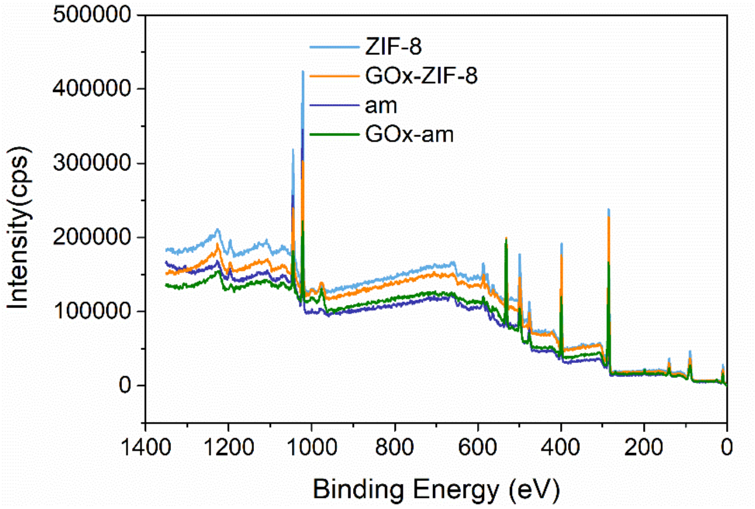

Supplementary Figure 5. XPS spectra (full). XPS spectra of ZIF-8, GOx-ZIF-8, am and GOx-am nanocomposites. Source data are provided as a Source Data file.

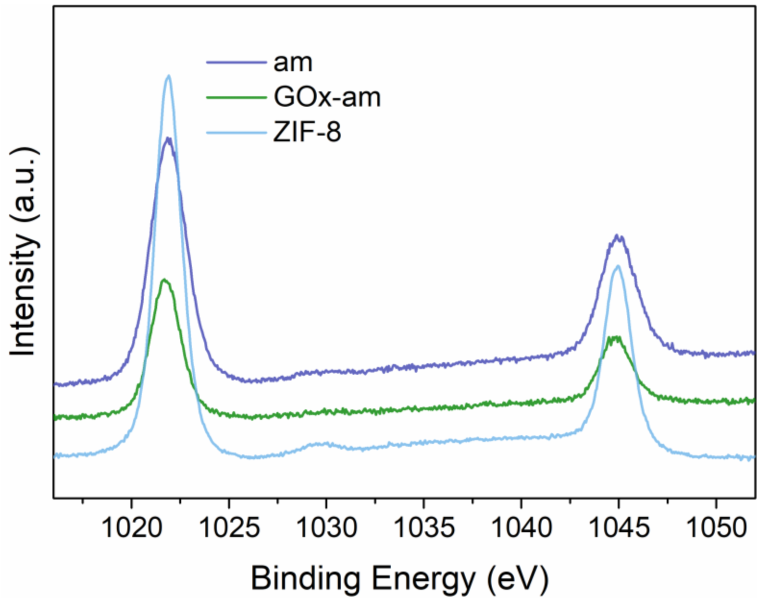

Supplementary Figure 6. High-resolution XPS spectra. High-resolution spectra of Zn *2p* for am, GOx-am nanocomposites and ZIF-8. Source data are provided as a Source Data file.

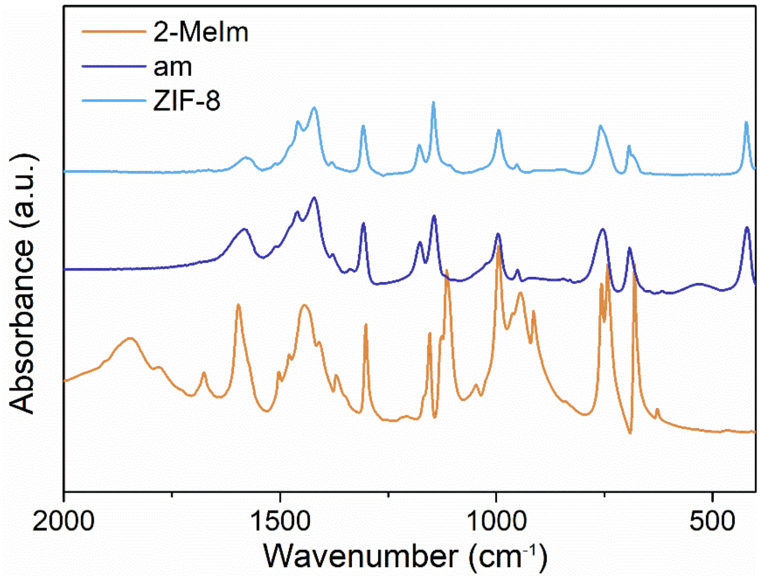

Supplementary Figure 7. FT-IR spectra. FT-IR spectra of 2-methylimidazole (2-MeIm), ZIF-8 and am. The FT-IR spectra suggested that a similar coordination between Zn and N was present in amorphous structure as in ZIF-8. Source data are provided as a Source Data file.

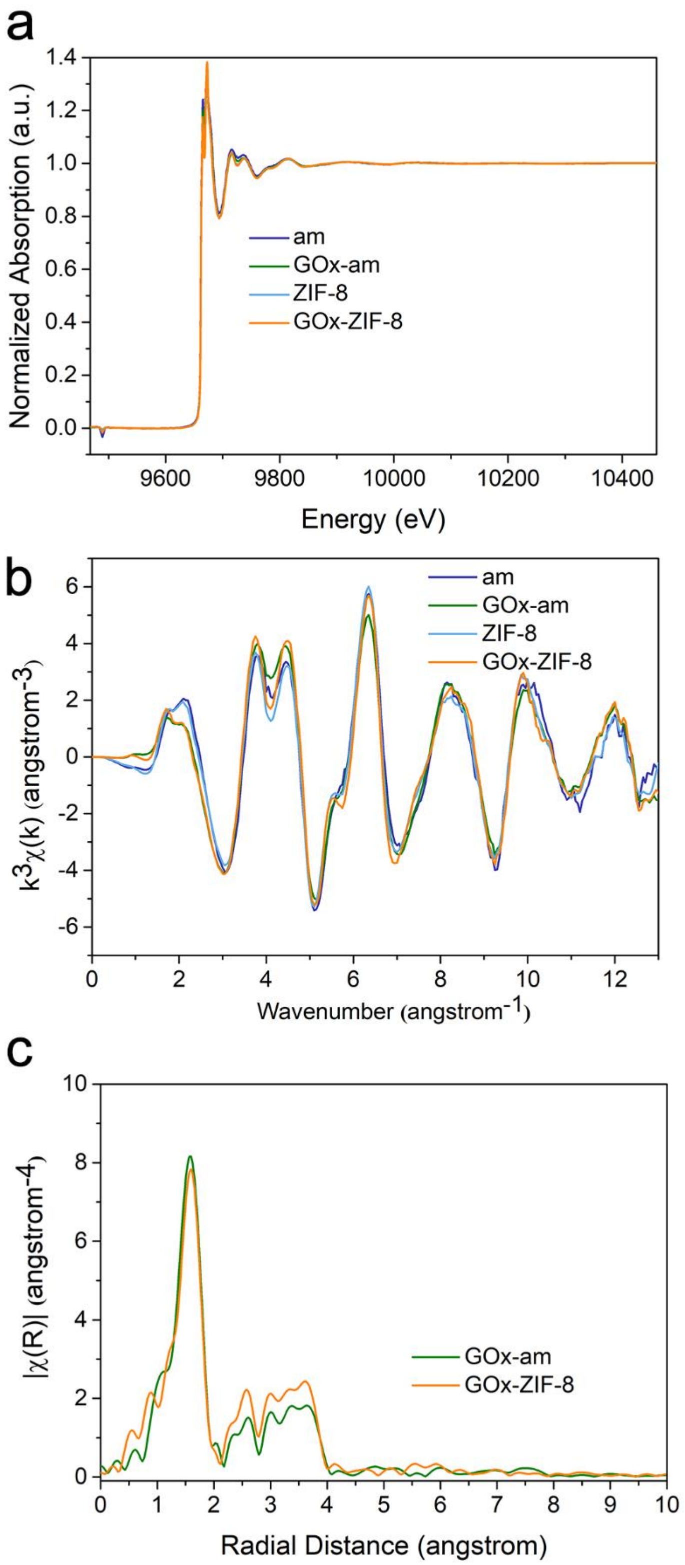

Supplementary Figure 8. X-ray adsorption spectra. X-ray adsorption spectra obtained at the K edge of zinc in E space (a), in k space (b) and in R space (c). Source data are provided as a Source Data file.

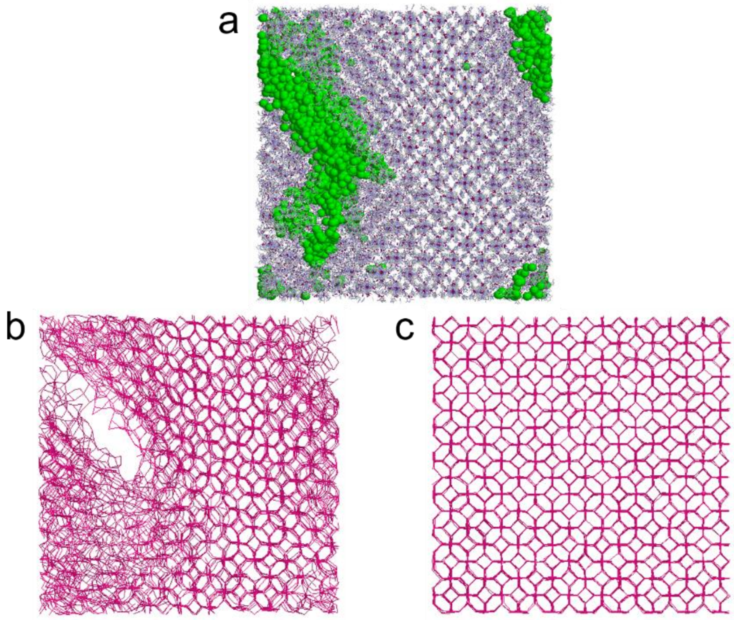

Supplementary Figure 9. Simulated structures. (a) Structure of equilibrated aZIF. The aZIF is shown in ball & stick model with CPK color scheme, where the atom Zn, C, O, H, N is colored deep pink, light grey, red, white, blue, respectively. The large pores formed was marked by green beads. The figure is prepared using Rasmol (http://rasmol.org). Simplified structure of aZIF (b) and ZIF-8 (c).

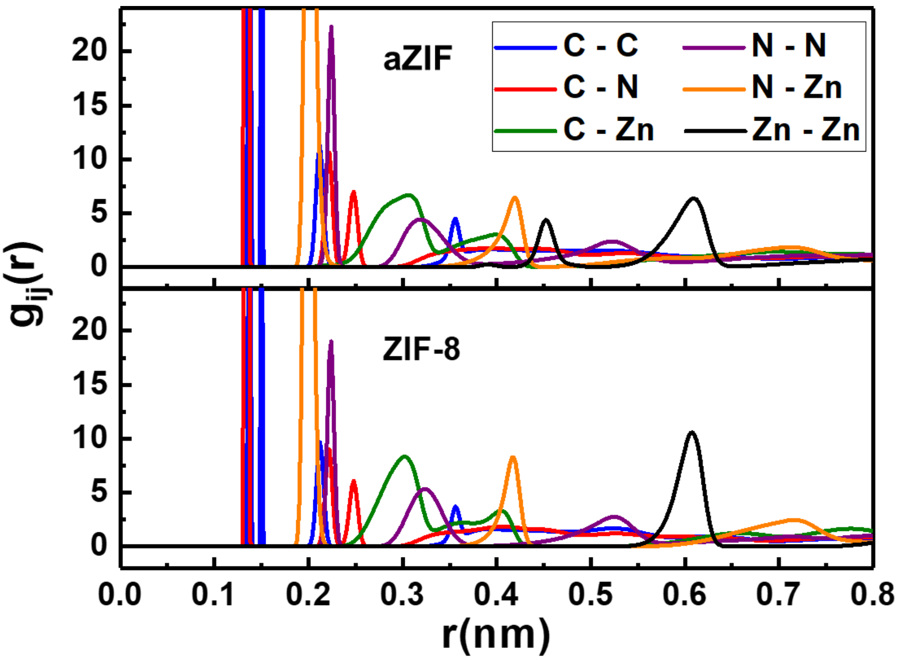

Supplementary Figure 10. Radial distribution functions of aZIF and ZIF-8. gij(r) of each atom pair between C and N of 2-MeIM, and Zn was calculated. Source data are provided as a Source Data file.

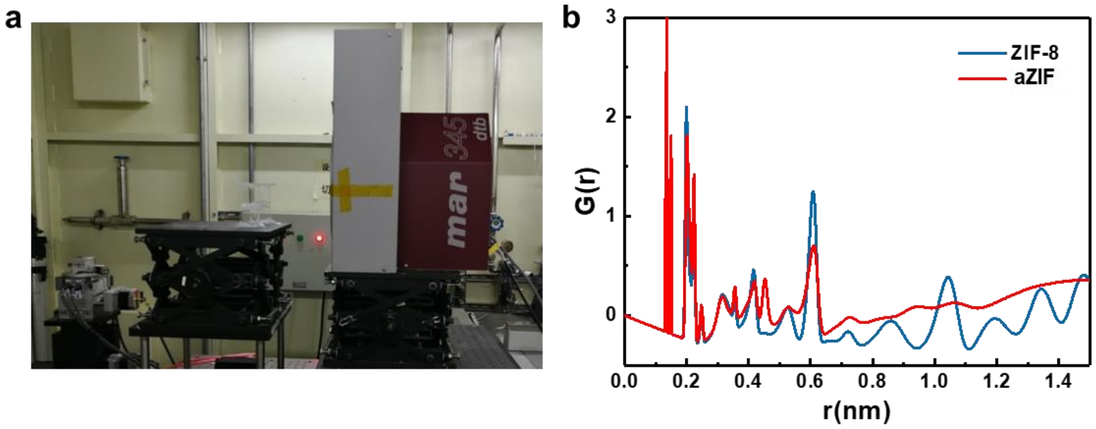

Supplementary Figure 11. PDF experimental set-up and PDF function. PDF experimental set-up at Shanghai Synchrotron Radiation Facilities (a) and PDF function from simulated model (b). The photo of PDF experimental set-up was taken by Xiaoling Wu. Source data are provided as a Source Data file.

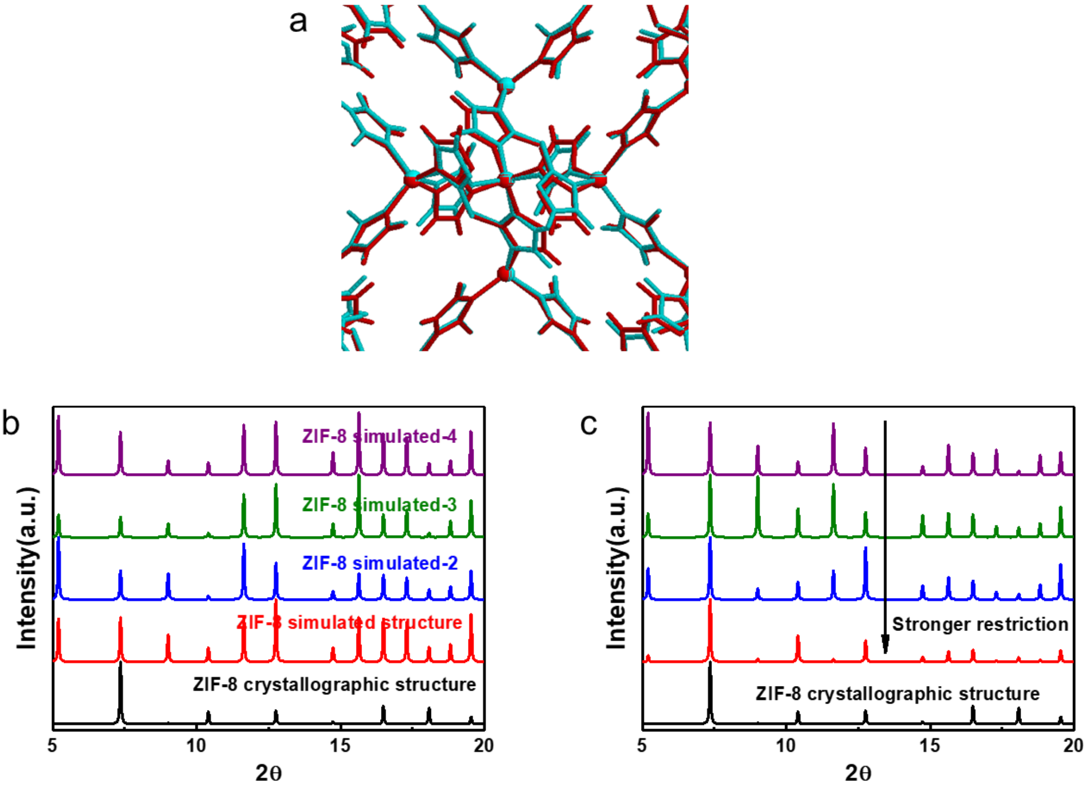

Supplementary Figure 12. Comparison of crystallographic structure and simulated model. (a) Mean atomic positions model for ZIF-8 (in cyan) superimposed on the crystallographic structure (in red). (b) XRD spectrum of ZIF-8 obtained from the simulation model in this study and comparison with crystallographic structure. (c) XRD spectrum of ZIF-8 obtained from the simulation model with stronger restriction. Source data are provided as a Source Data file.

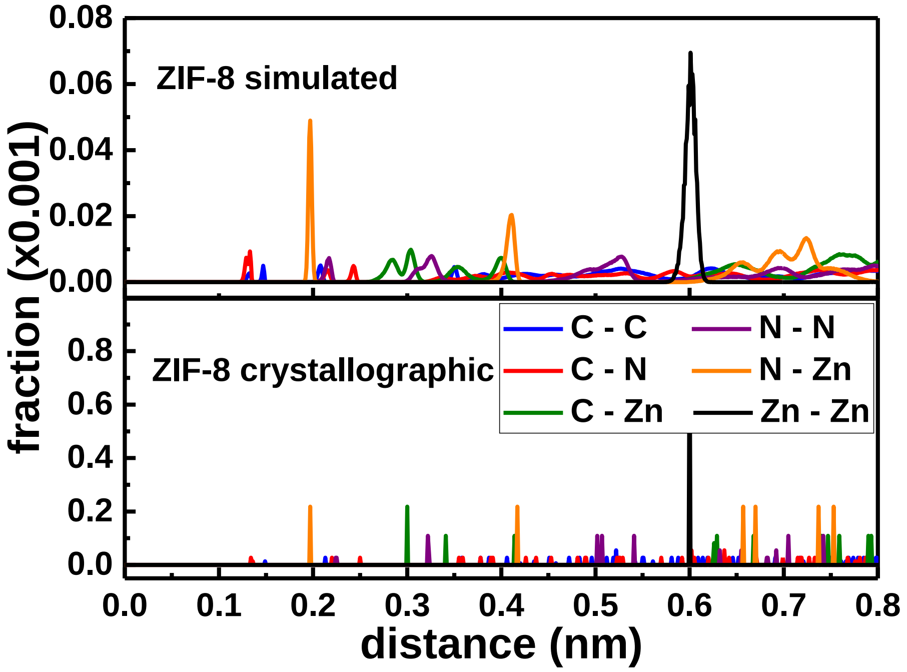

Supplementary Figure 13. Atom pair distance distribution. ZIF-8 simulated: simulated structure of ZIF-8. ZIF-8 crystallographic: crystallographic structure of ZIF-8. The atom pair distance distribution in the simulated structure and crystallographic structure of ZIF-8 was calculated using our in-house scripts. Source data are provided as a Source Data file.

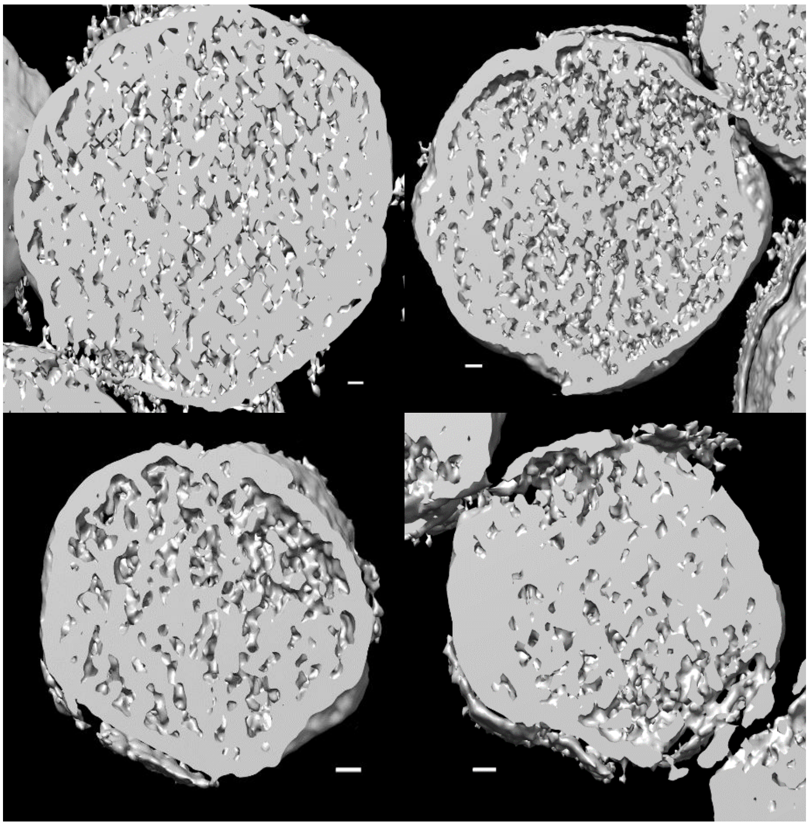

Supplementary Figure 14. Several Cryo-ET images of a single GOx-aZIF nanocomposite. The cross-section of GOx-aZIF nanocomposites exhibited spherical particles with mesopores plainly distributed inside the particle. The scale bar is 10 nm.

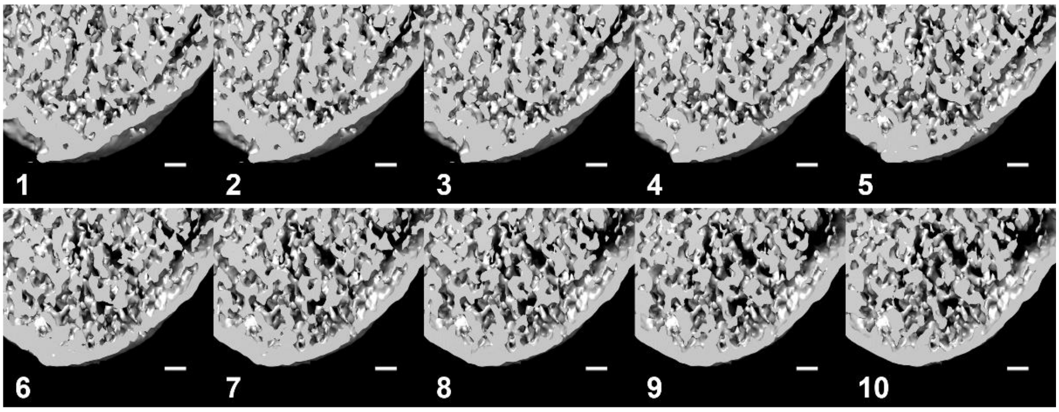

Supplementary Figure 15. A series of cross sections of a single GOx-aZIF nanocomposite. This series of images suggested that the generated mesopores were interconnected. The scale bar is 10 nm.

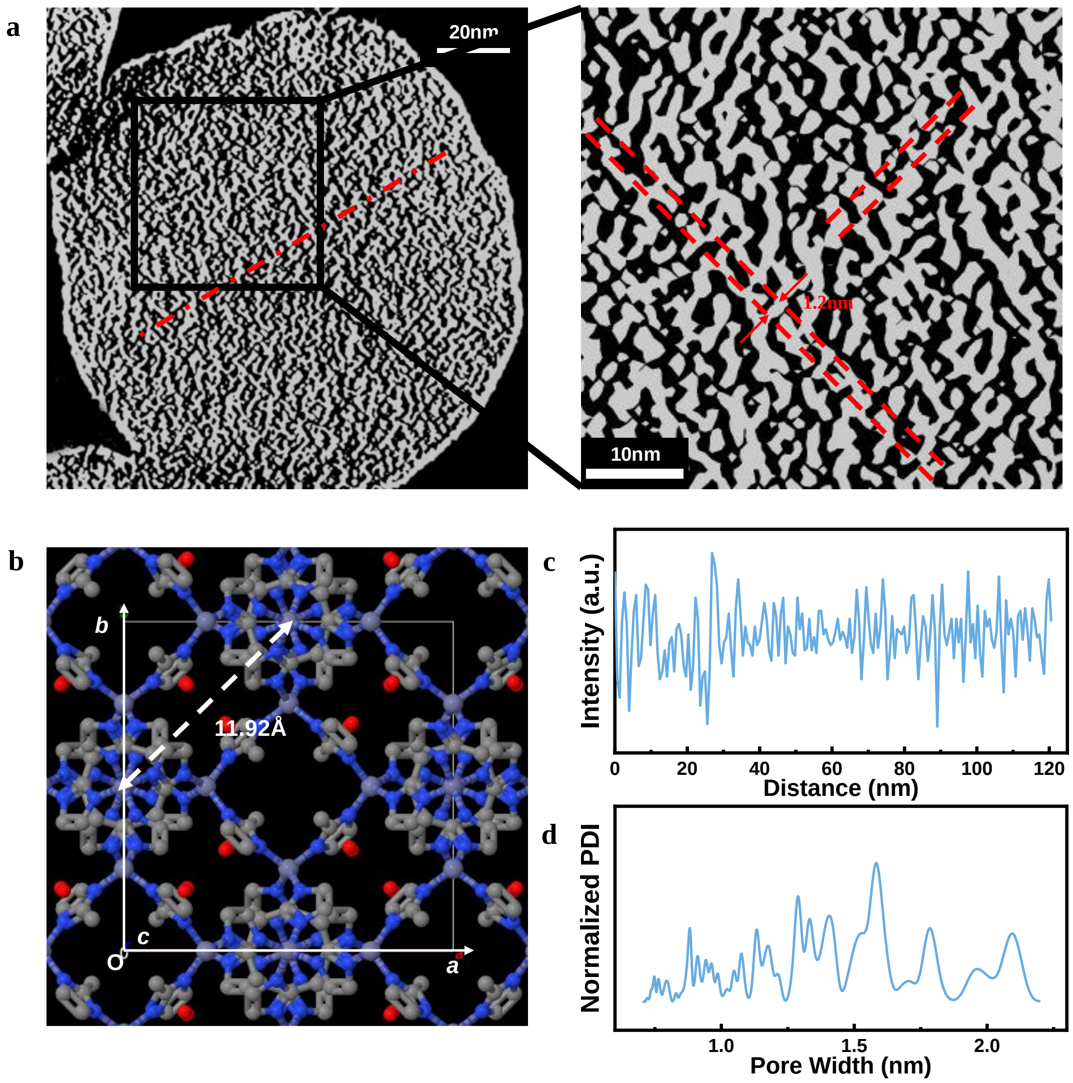

Supplementary Figure 16. Cryo-electron tomography (Cryo-ET) reconstruction of a single crystalline ZIF-8 and pore size analysis. Cryo-ET reconstruction and its zoomed image of a single crystalline ZIF-8 (a) and unit cell of ZIF-8 with its interplanar spacing of planes (1 1 0) (b), data was collected from Cambridge Crystallographic Data Centre (CCDC) (DOI: 10.5517/ccy0cy7). Linear scan of electron density along the dashed line (c) and fast Fourier transformation of the electron density linear scan are converted to pore size distribution (d) of the crystalline ZIF-8. Normalized PDI: normalized pore distribution intensity. Source data are

provided as a Source Data file.

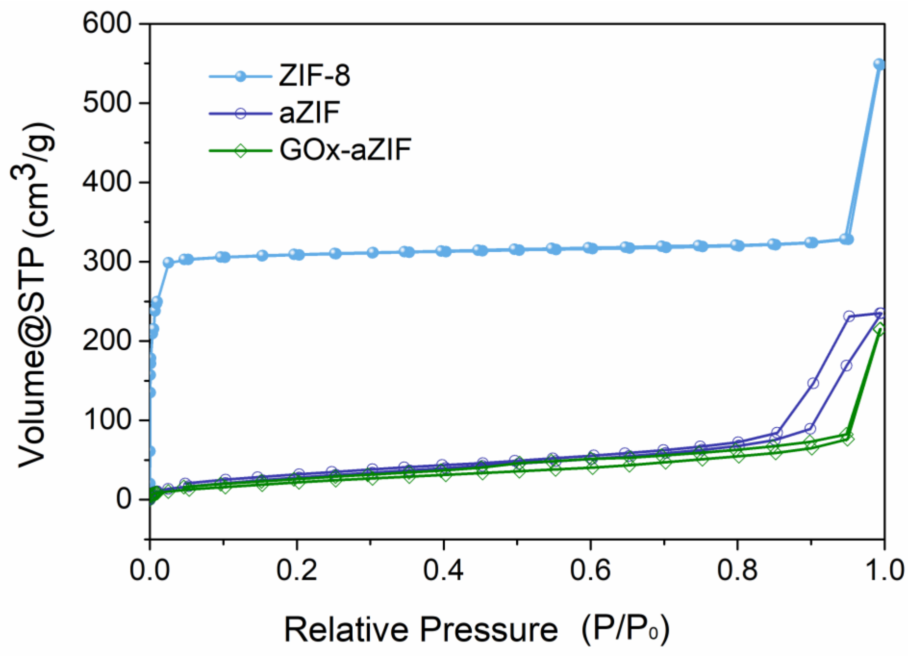

Supplementary Figure 17. Nitrogen adsorption and desorption curves. Nitrogen adsorption and desorption curves of ZIF-8, amorphous ZIF and GOx-aZIF nanocomposites. Source data are provided as a Source Data file.

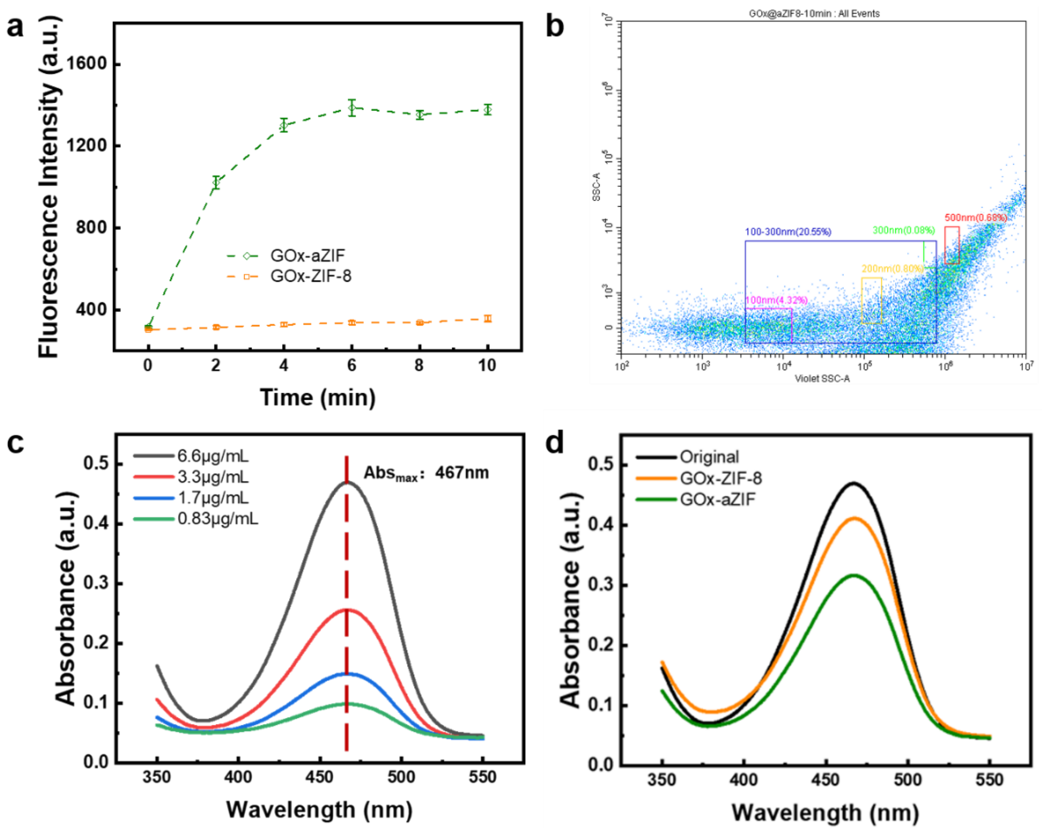

Supplementary Figure 18. Determination of substrate transport in GOx-ZIF-8 and GOx-aZIF nanocomposites. (a) Kinetics of fluorescent glucose analogues entering GOx-ZIF-8 and GOx-aZIF nanocomposites. Data were represented as mean ± SD (n=3). (b) An example of gating strategy. The gates for certain particle size (100 nm, 200 nm, 300 nm etc.) was calibrated using particles with known sizes (100nm, 200nm, 300 nm, etc.). Since the GOx-aZIF and GOx-ZIF-8 possessed the size around 100 nm and slight agglomeration (2-3 particles stuck together) would occur according to DLS data, these gates were then used to determine enzyme-nanoparticles with size around 100-300 nm. (c) Absorbance spectra of 2-NBDG (methanol) with different concentrations. (d) Comparison of absorbance spectra of original 2-NBDG methanol solution and the supernatants (methanol) for

GOx-ZIF-8 and GOx-aZIF, respectively. The experiment was repeated for 3 times. Source data are provided as a Source Data file.

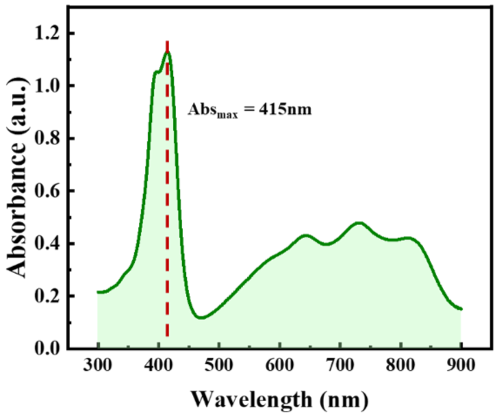

Supplementary Figure 19. Absorbance spectrum of ABTS·$^-$. The absorbance spectrum showed a peak value at 415nm. Source data are provided as a Source Data file.

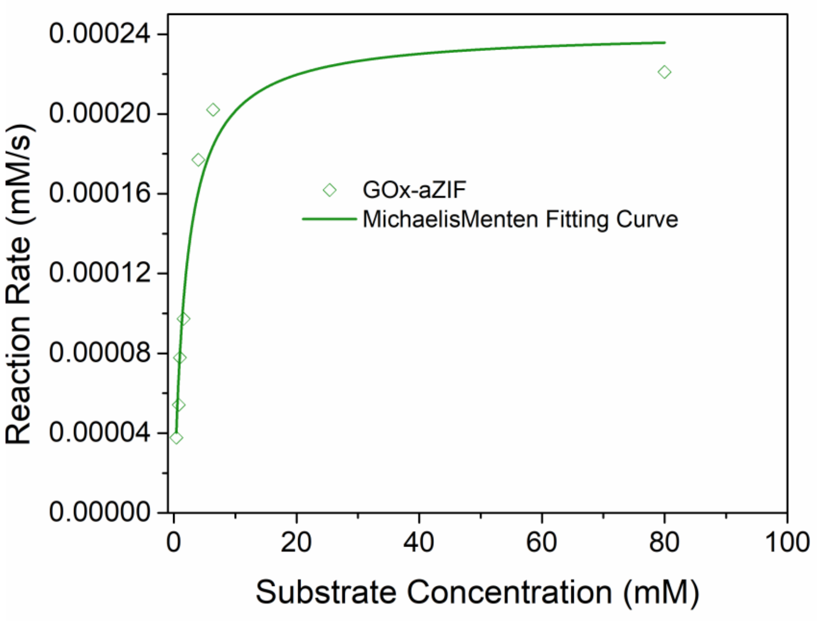

Supplementary Figure 20. Michaelis-Menten fitting of GOx-aZIF nanocomposites. Source data are provided as a Source Data file.

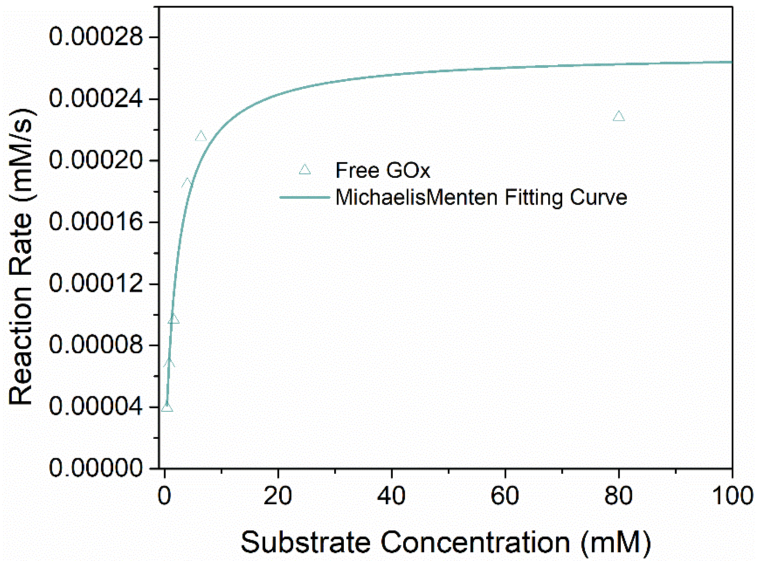

Supplementary Figure 21. Michaelis-Menten fitting of free GOx.

Source data are provided as a Source Data file.

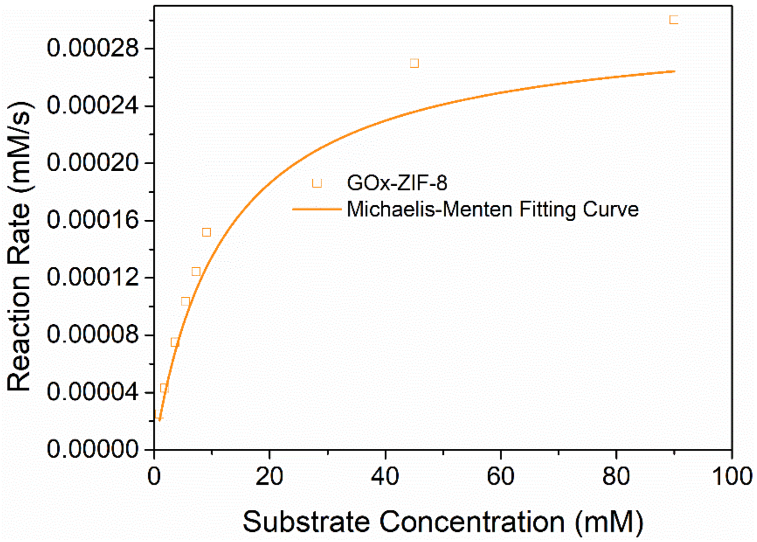

Supplementary Figure 22. Michaelis-Menten fitting for different glucose concentration for GOx-ZIF-8. Source data are provided as a Source Data file.

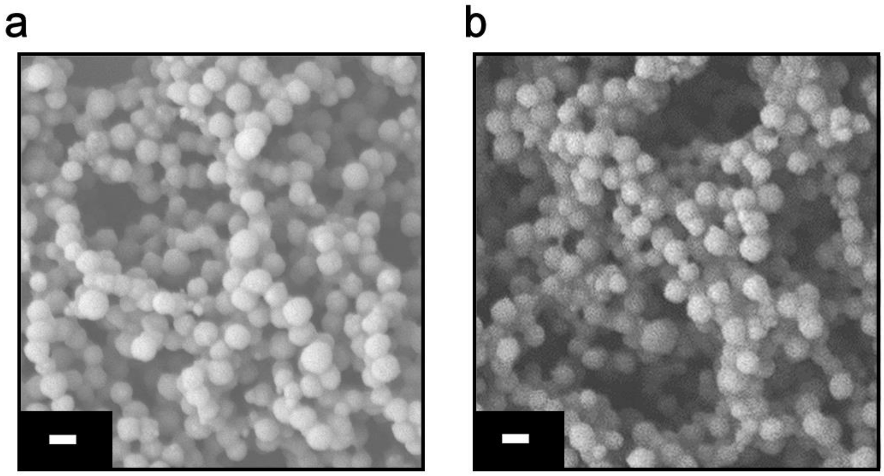

Supplementary Figure 23. SEM images of enzyme-aZIF nanocomposites. SEM images of (a) CALB-aZIF nanocomposites, (b) catalase-aZIF nanocomposites. Scale bars: 100 nm.

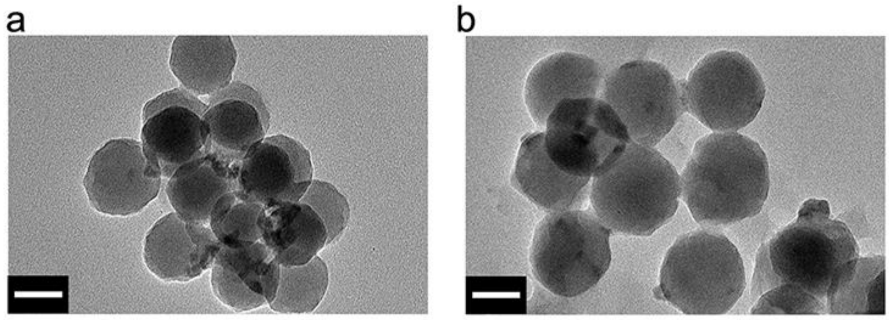

Supplementary Figure 24. TEM images of enzyme-aZIF nanocomposites. TEM images of (a) CALB-aZIF nanocomposites, (b) catalase-aZIF nanocomposites. These enzyme-aZIF nanocomposites showed similar spherical structures. Scale bars: 50 nm.

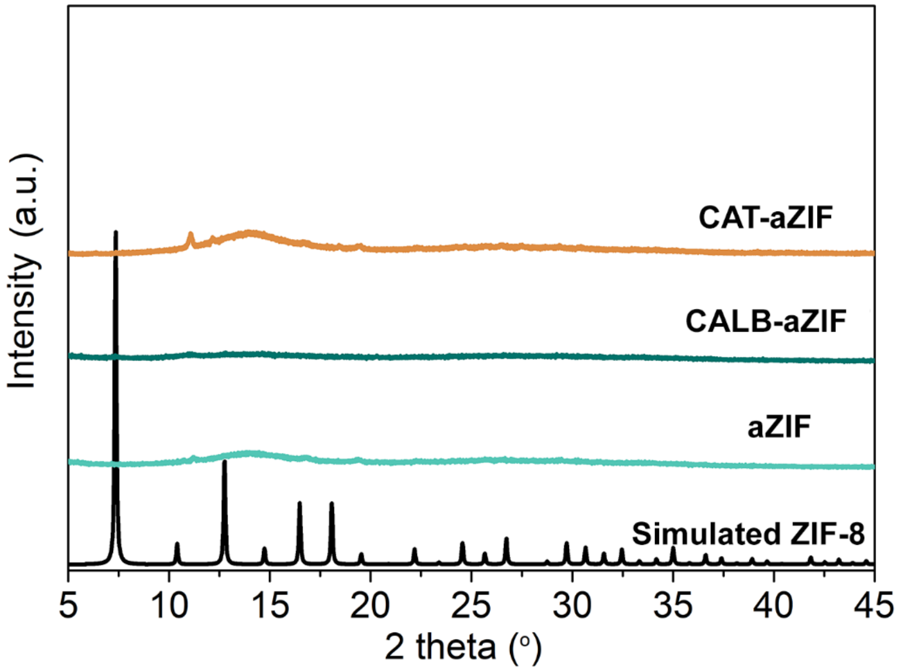

Supplementary Figure 25. XRD patterns of different enzyme-aZIF nanocomposites. CALB: *Candida antarctic* lipase B, CAT: catalase. These enzyme-aZIF nanocomposites also exhibited the amorphous structures. Source data are provided as a Source Data file.

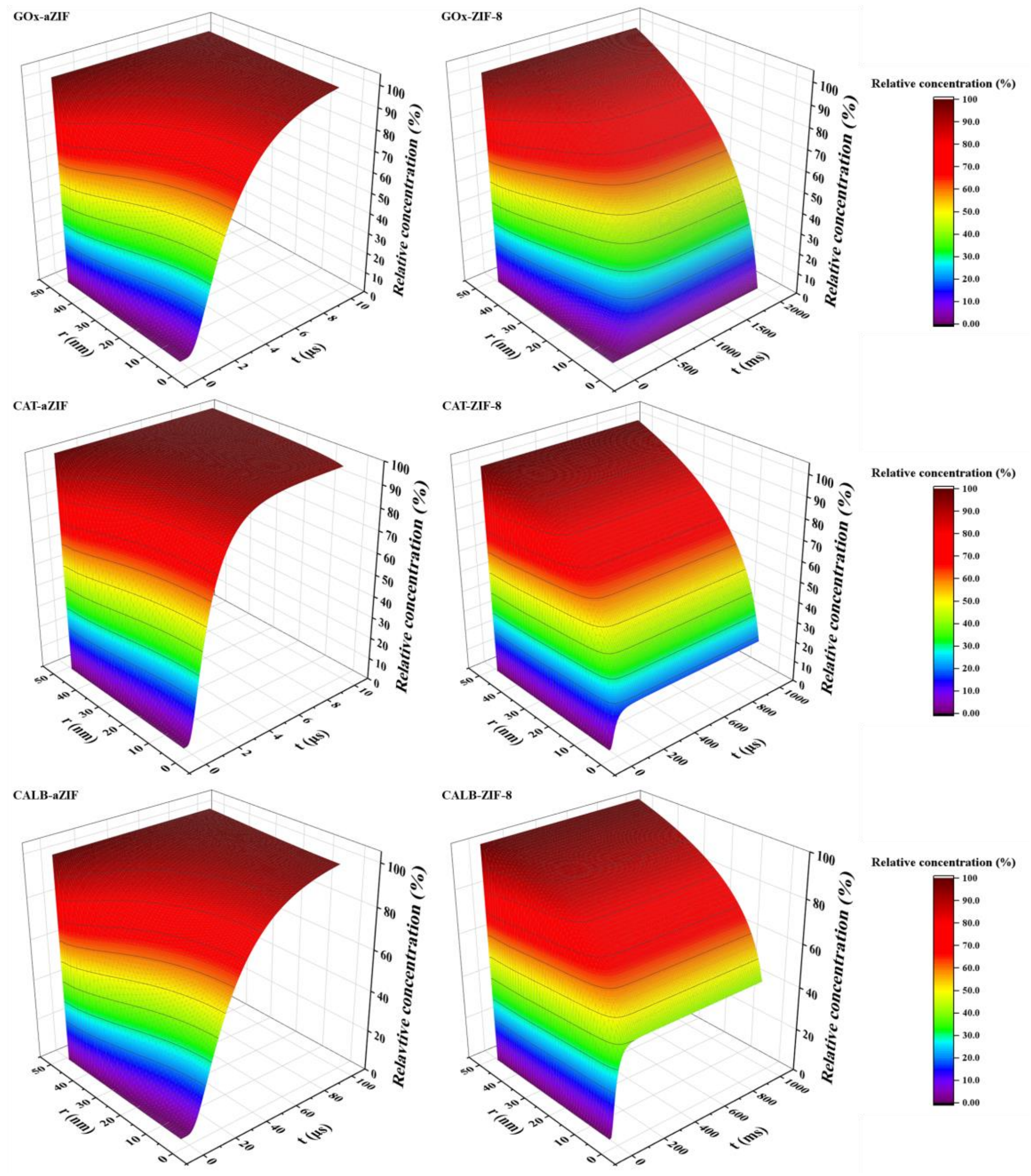

Supplementary Figure 26. Substrate concentration distribution inside enzyme-nanocomposites. Substrate concentration distribution inside enzyme-aZIF and enzyme-ZIF-8 composite based on diffusion-reaction model. Source data are provided as a Source Data file.

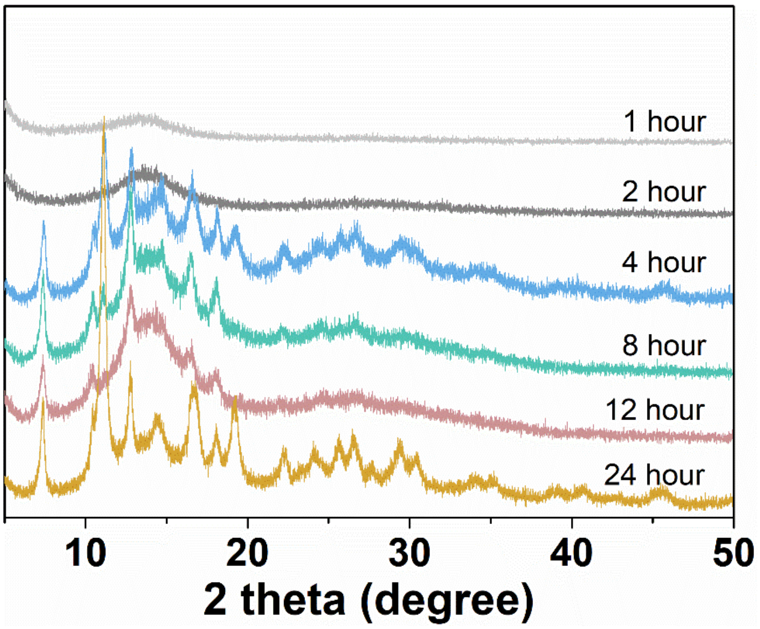

Supplementary Figure 27. XRD patterns of aZIF with different synthesis time. Source data are provided as a Source Data file.

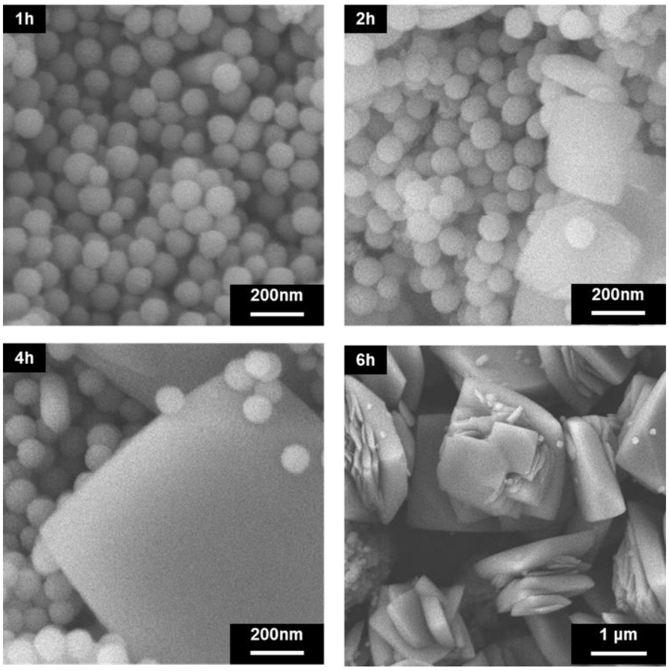

Supplementary Figure 28. Morphology evolution of aZIF with varying reaction time.

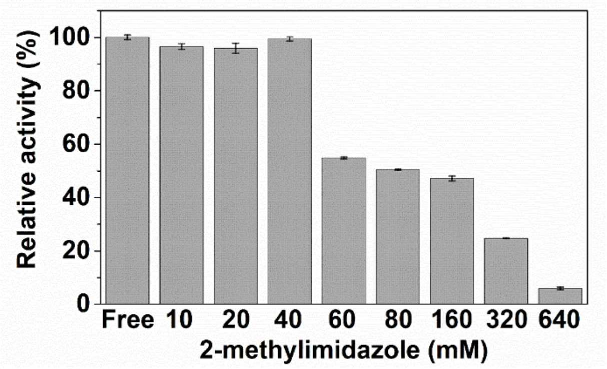

Supplementary Figure 29. Activity of GOx-aZIF composites prepared with different starting 2-methylimidazole concentrations. Source data are provided as a Source Data file. Data were represented as mean ± SD (n=3).

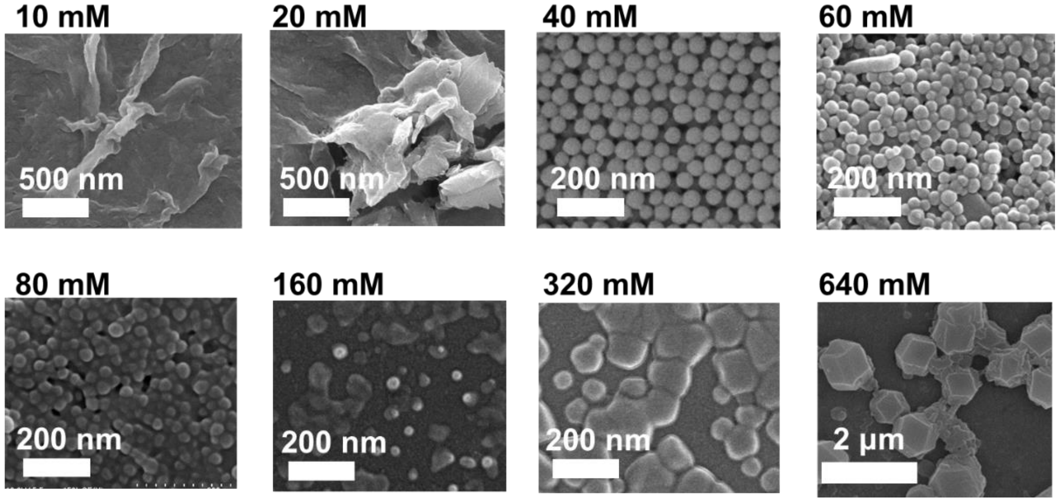

Supplementary Figure 30. SEM images. SEM images of GOx-aZIF composites prepared with different starting 2-methylimidazole concentrations.

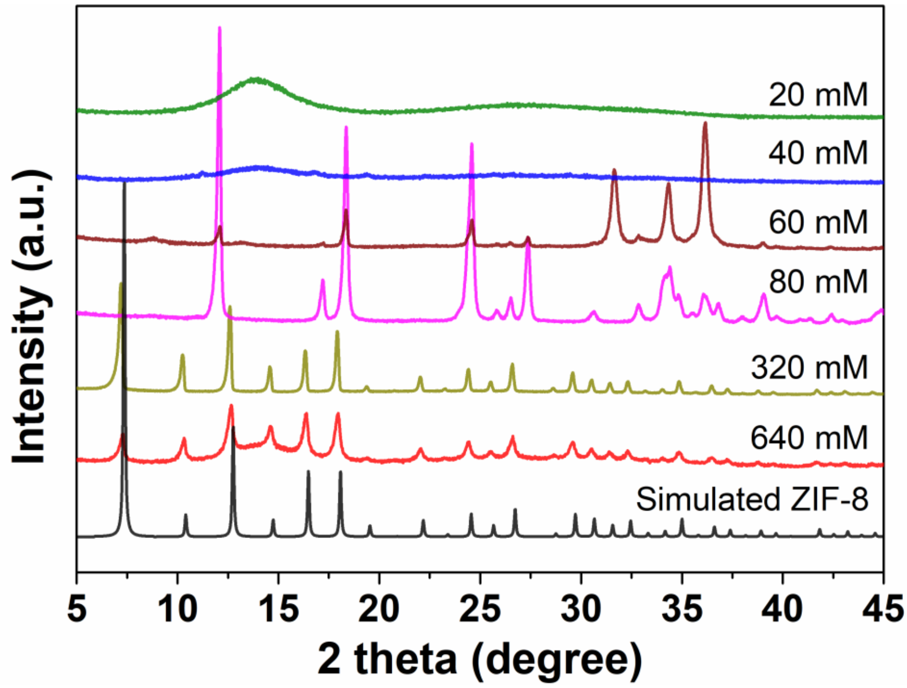

Supplementary Figure 31. XRD patterns of composites with different starting 2-methylimidazole concentrations. Source data are provided as a Source Data file.

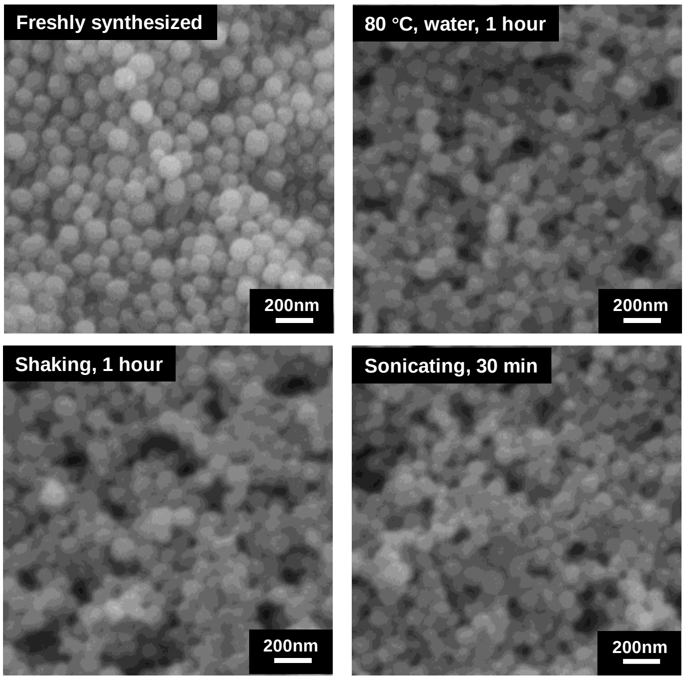

Supplementary Figure 32. SEM images. SEM images of aZIF of freshly synthesized, incubated in water at 80 °C for 1h, shaken for 1h and sonicated for 30min.

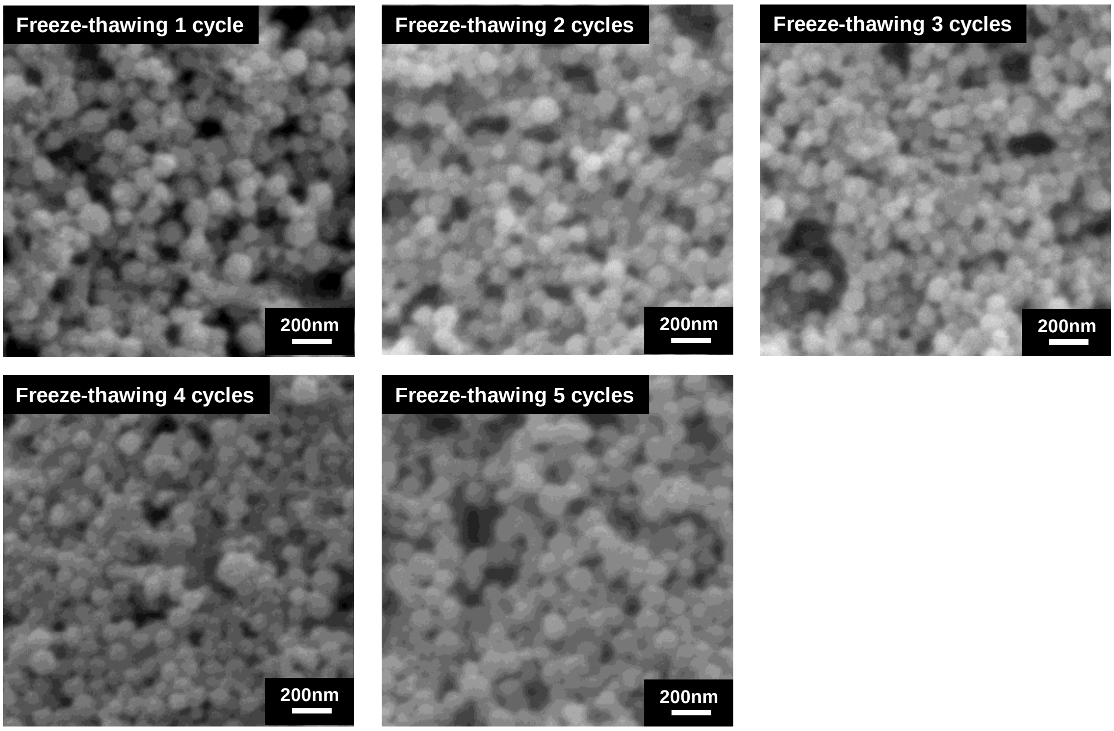

Supplementary Figure 33. SEM images of GOx-aZIF of freeze-thawing cycles. The structure remained unchanged after 5 freeze-thawing cycles.

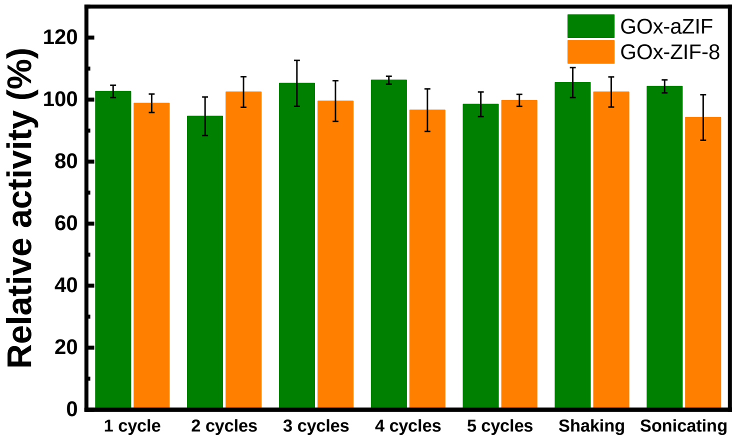

Supplementary Figure 34. Relative activity of GOx-aZIF. Relative activity of GOx-aZIF during the freeze-thawing cycles and after shaking for 1h and sonicating for 30min. Data were represented as mean ± SD (n=3). Source data are provided as a Source Data file.

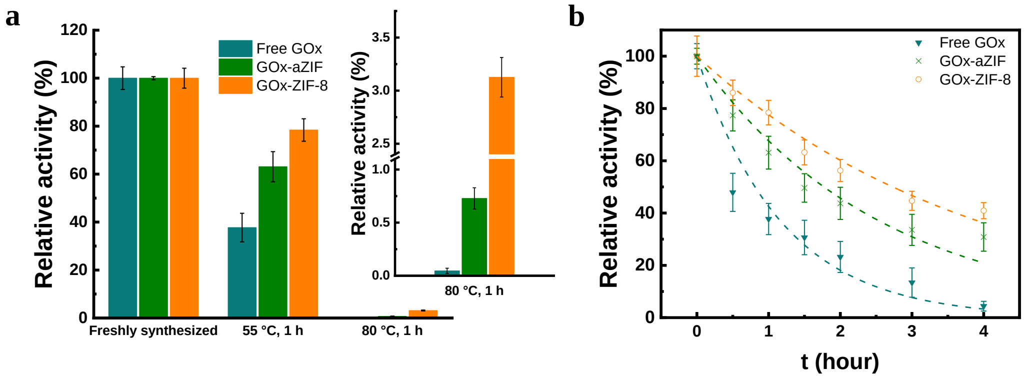

Supplementary Figure 35. Thermal stability. Thermal stability of free GOx, GOx-aZIF and GOx-ZIF-8. (a) Relative activity of free GOx, GOx-aZIF and GOx-ZIF-8 after incubation in aqueous solution at 55 ºC and 80 °C for 1 h. (b) Thermal deactivation kinetics of free GOx, GOx-aZIF and GOx-ZIF-8 at 55 °C. Data were represented as mean ± SD (n=3). Source data are provided as a Source Data file.

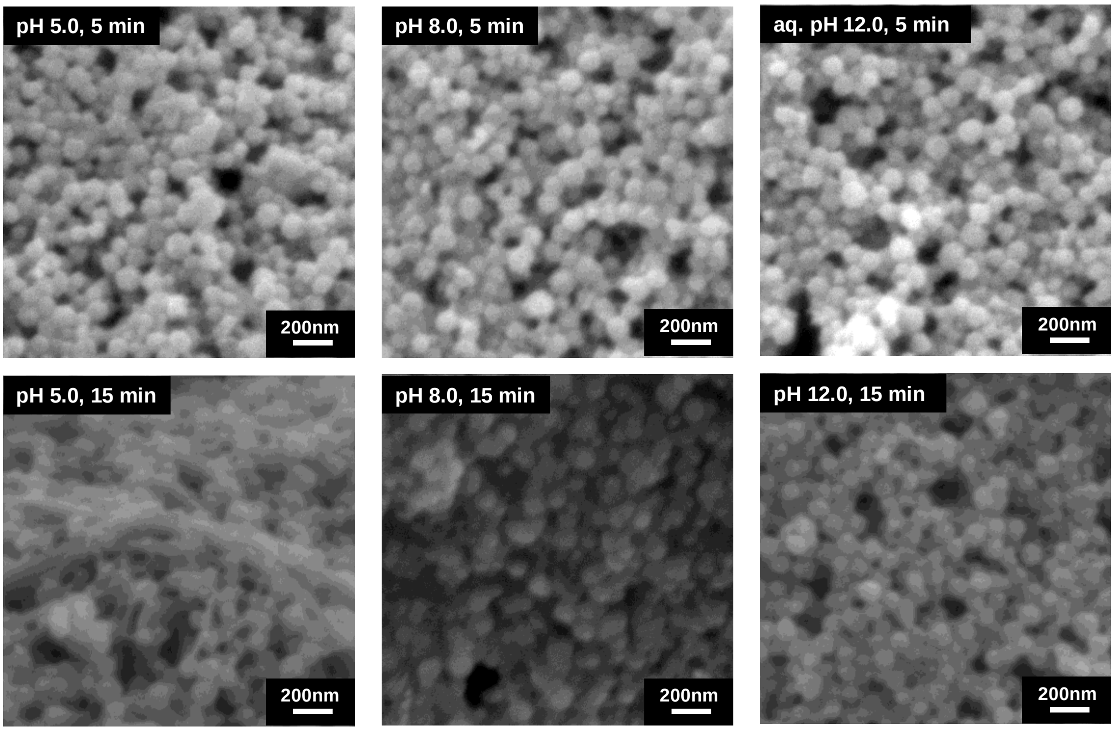

Supplementary Figure 36. SEM images of GOx-aZIF. SEM images of GOx-aZIF after incubating in acetate buffer (pH 5.0), HEPES buffer (pH 8.0) and NaOH solution (pH 12.0).

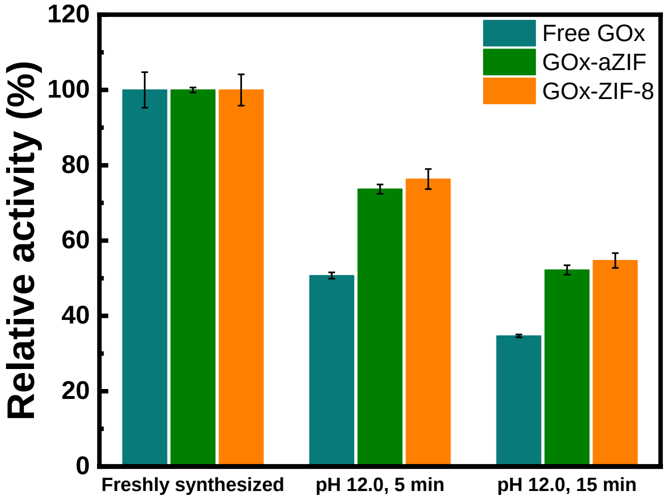

Supplementary Figure 37. Activity retention of enzymes. Activity retention of free GOx, GOx-aZIF and GOx-ZIF-8 after incubating at alkaline solution (pH 12.0). Data were represented as mean ± SD (n=3). Source data are provided as a Source Data file.

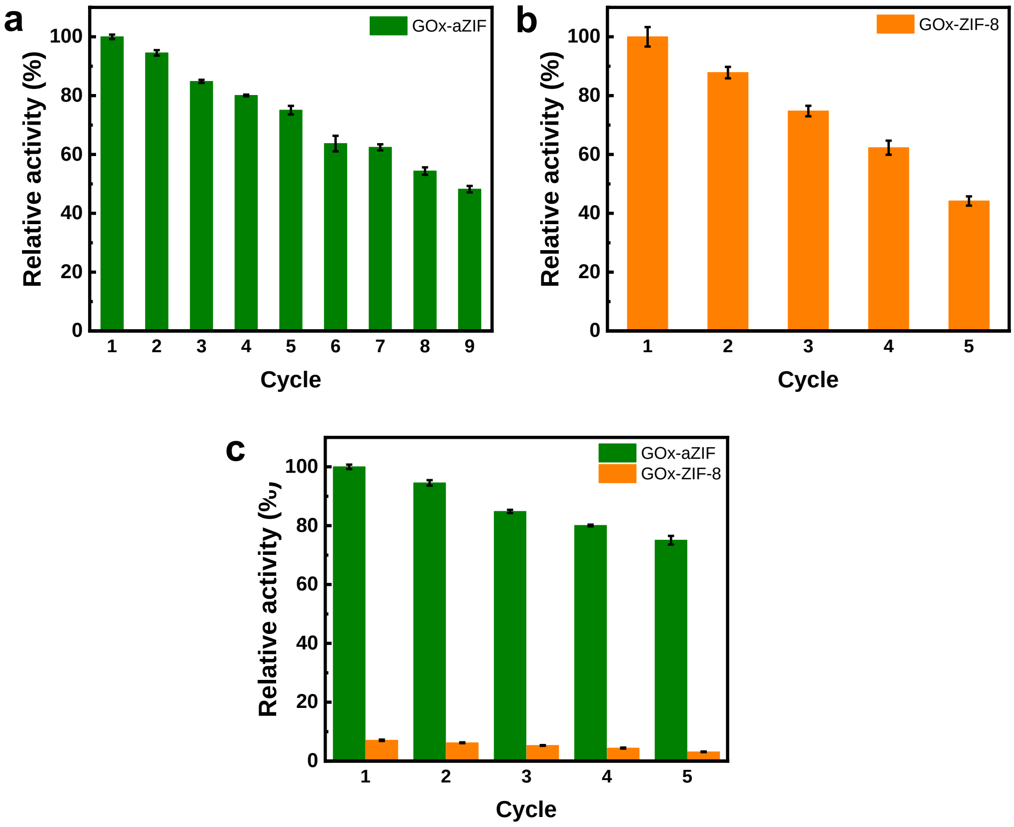

Supplementary Figure 38. Recycle performance of enzyme-nanocomposites. Recycle performance of GOx-aZIF (a) and GOx-ZIF-8 (b) and the comparison of relative activities (c). Data were represented as mean ± SD (n=3). Source data are provided as a Source Data file.

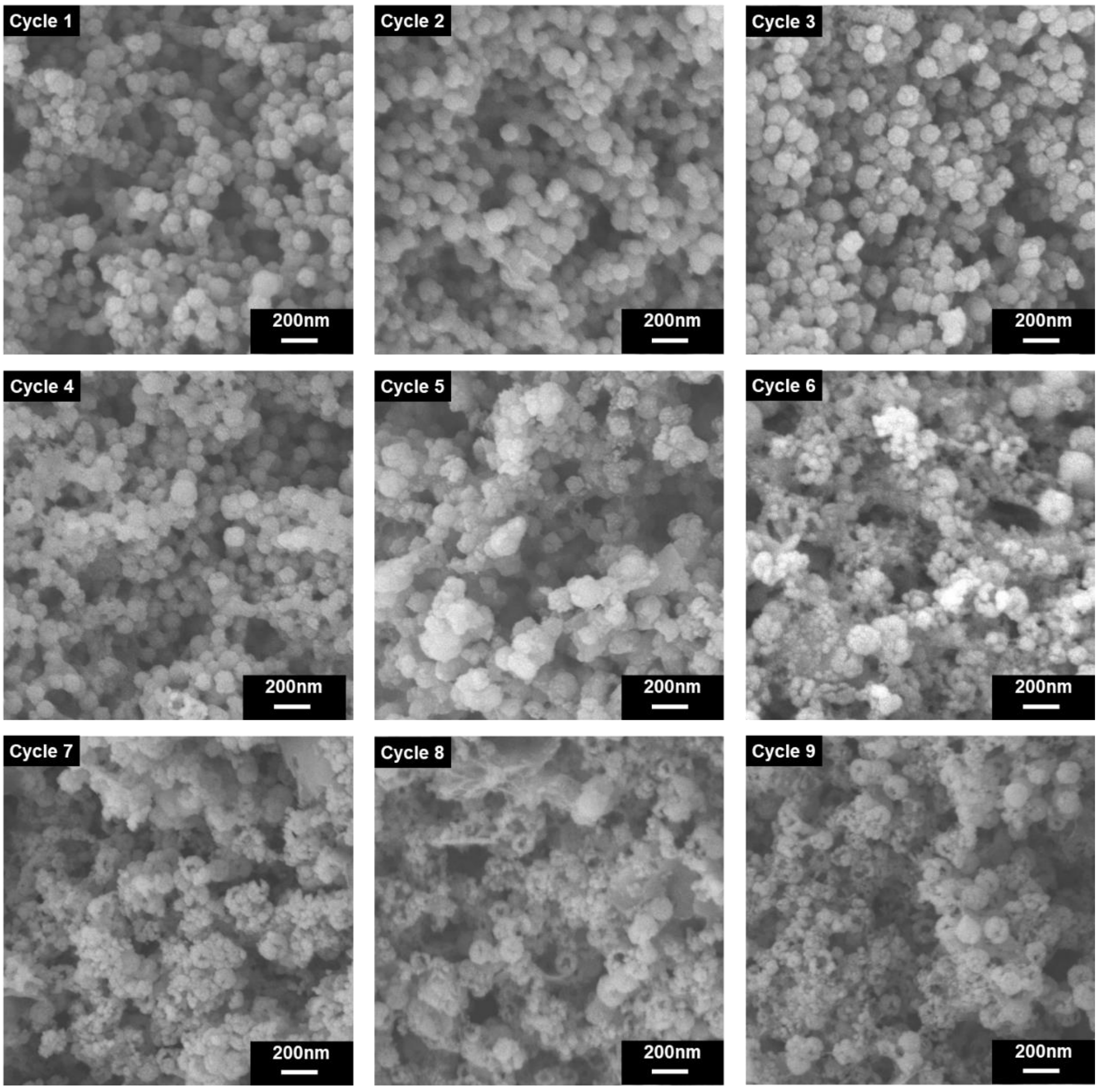

Supplementary Figure 39. Morphologies of GOx-aZIF after each reusing cycle. The SEM images demonstrated that the GOx-aZIF could remain its spherical structure in 5 cycles.

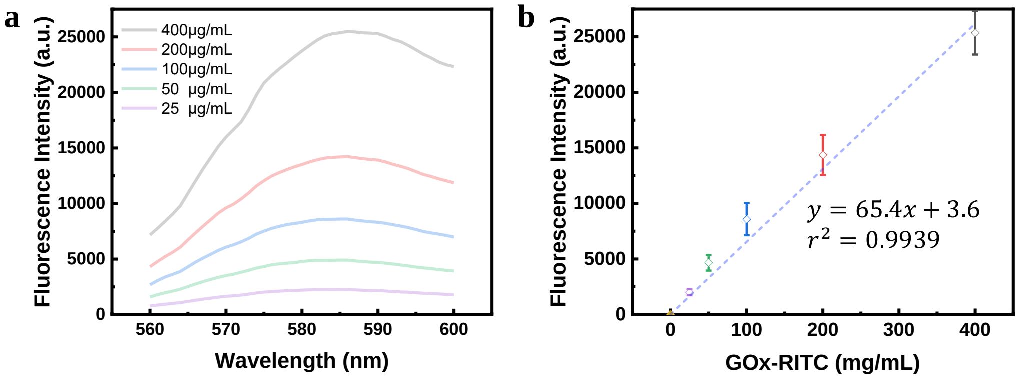

Supplementary Figure 40. Determination of enzyme release during the activity test. (a) Fluorescence emission spectra of GOx-RITC with various concentrations. (b) the calibration curve between fluorescence intensity and concentration of GOx-RITC. Data were represented as mean ± SD (n=3). Source data are provided as a Source Data file.

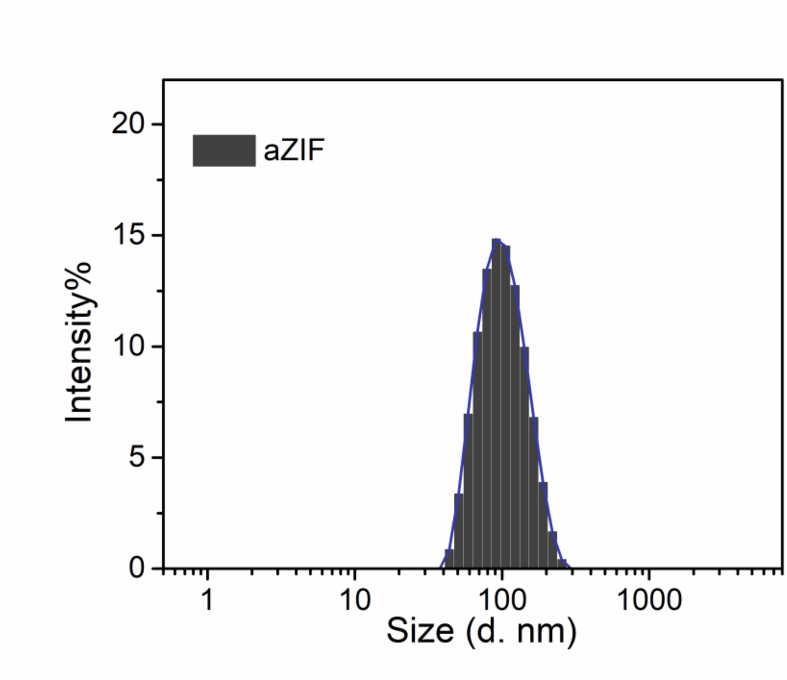

Supplementary Figure 41. Size distribution of aZIF. Size distribution of aZIF in phosphate buffer saline, determined by DLS. Source data are provided as a Source Data file.

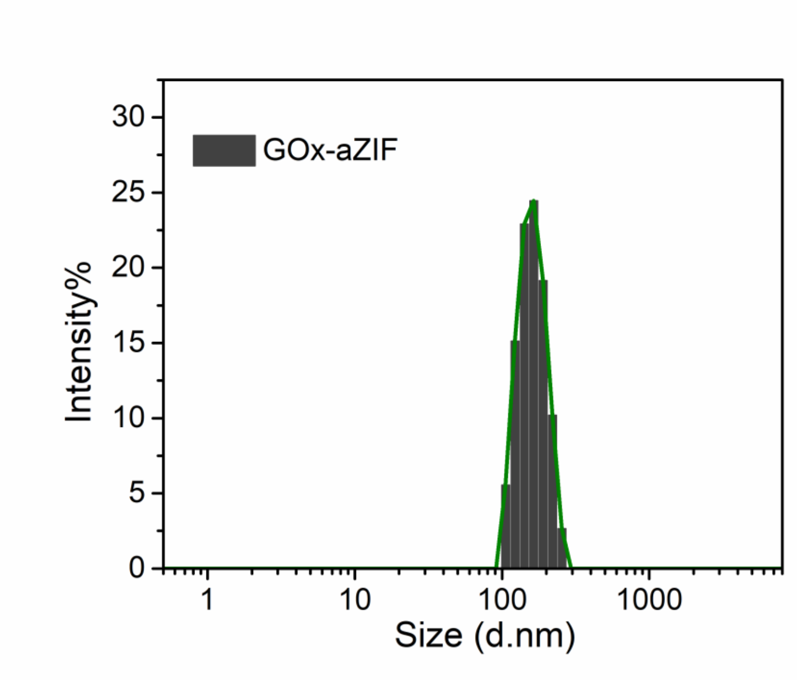

Supplementary Figure 42. Size distribution of GOx-aZIF. Size distribution of GOx-aZIF nanocomposites in phosphate buffer saline, determined by DLS. Source data are provided as a Source Data file.

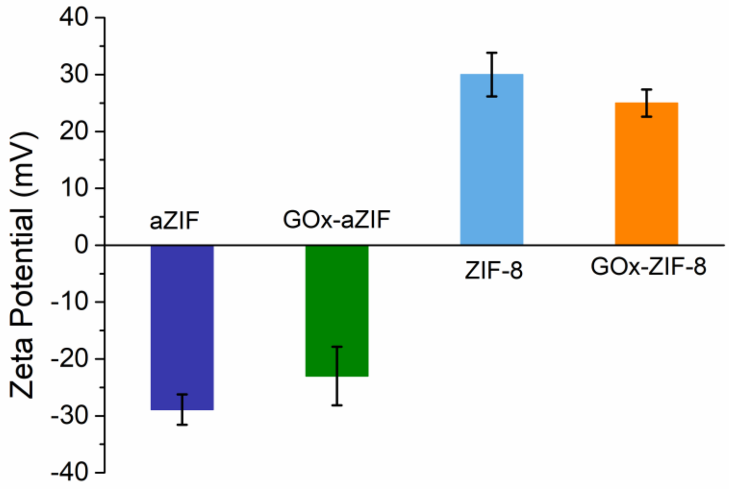

Supplementary Figure 43. Zeta potentials. Zeta potentials of aZIF, GOx-aZIF nanocomposites, ZIF-8 and GOx-ZIF-8 nanocomposites. Data were represented as mean ± SD (n=3). Source data are provided as a Source Data file.

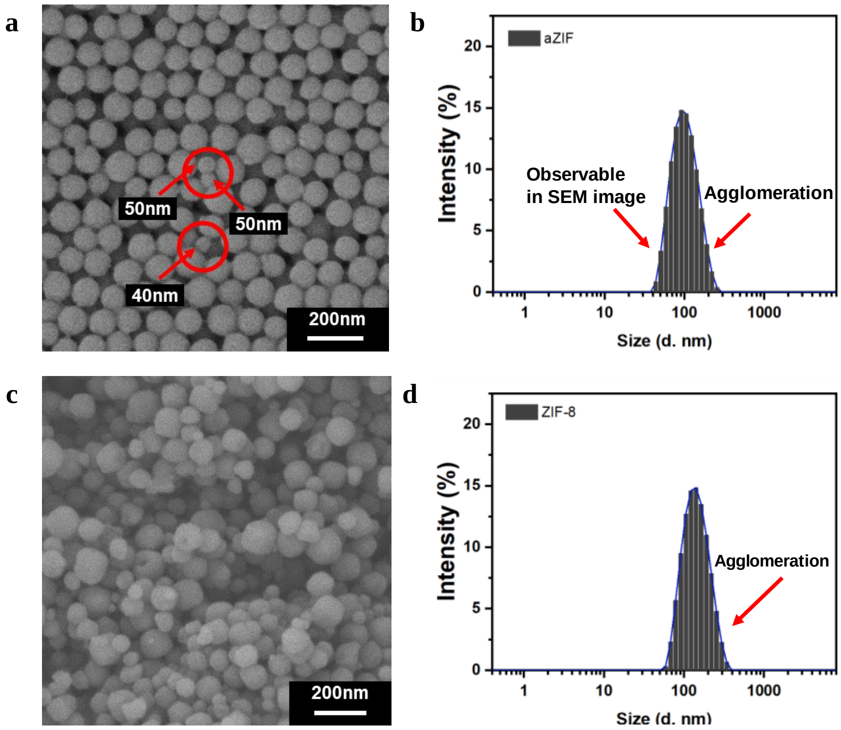

Supplementary Figure 44. SEM images and size distributions. SEM images (particles with sizes below the average size were circled in red) and size distributions by dynamic light scattering for aZIF (a, b) and ZIF-8 (c, d). Source data are provided as a Source Data file.

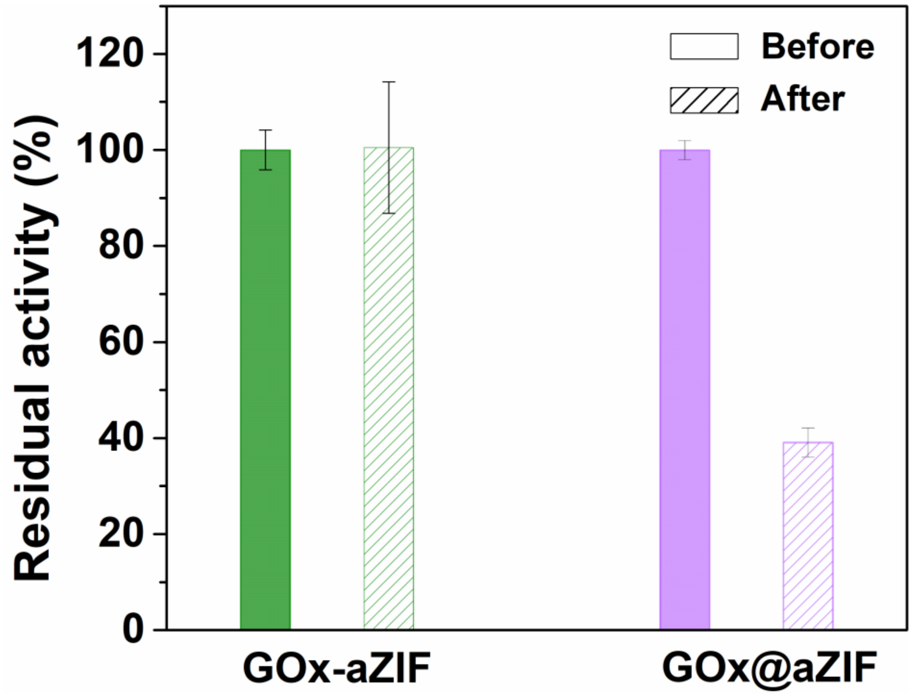

Supplementary Figure 45. Stability of GOx-aZIF and GOx@aZIF against protease digestion under 40 °C for 3h. GOx@aZIF was prepared by physical adsorption of GOx on the surface of aZIF. Data were represented as mean ± SD (n=3). Source data are provided as a Source Data file.

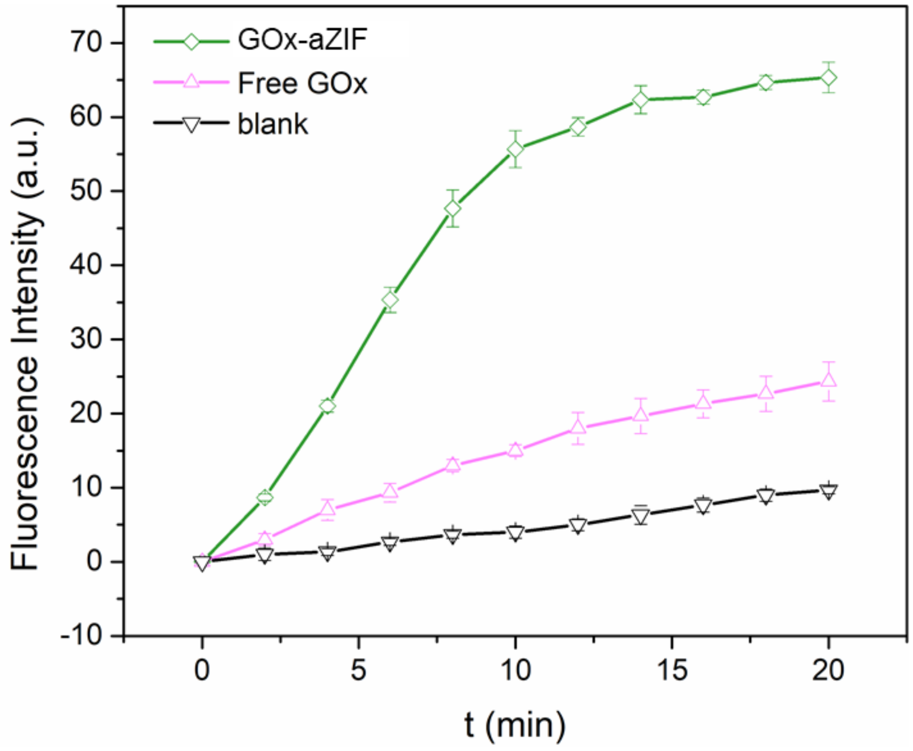

Supplementary Figure 46. Detection of glucose concentration in cell supernatant using GOx-aZIF or free GOx treated by trypsin. Data were represented as mean ± SD (n=3). Source data are provided as a Source Data file.

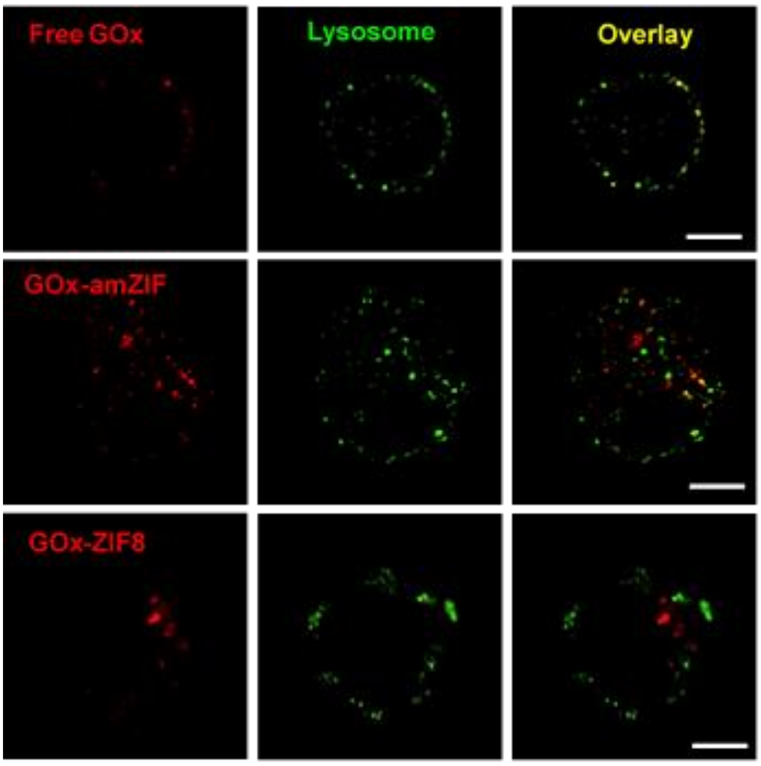

Supplementary Figure 47. SIM images showing the intracellular localization of different enzyme composites. GOx molecules were labeled with Rhodamine B isothiocyanate (RITC, red), while lysosomes were stained with the DND tracker (green). Scale bars, 5 μm.

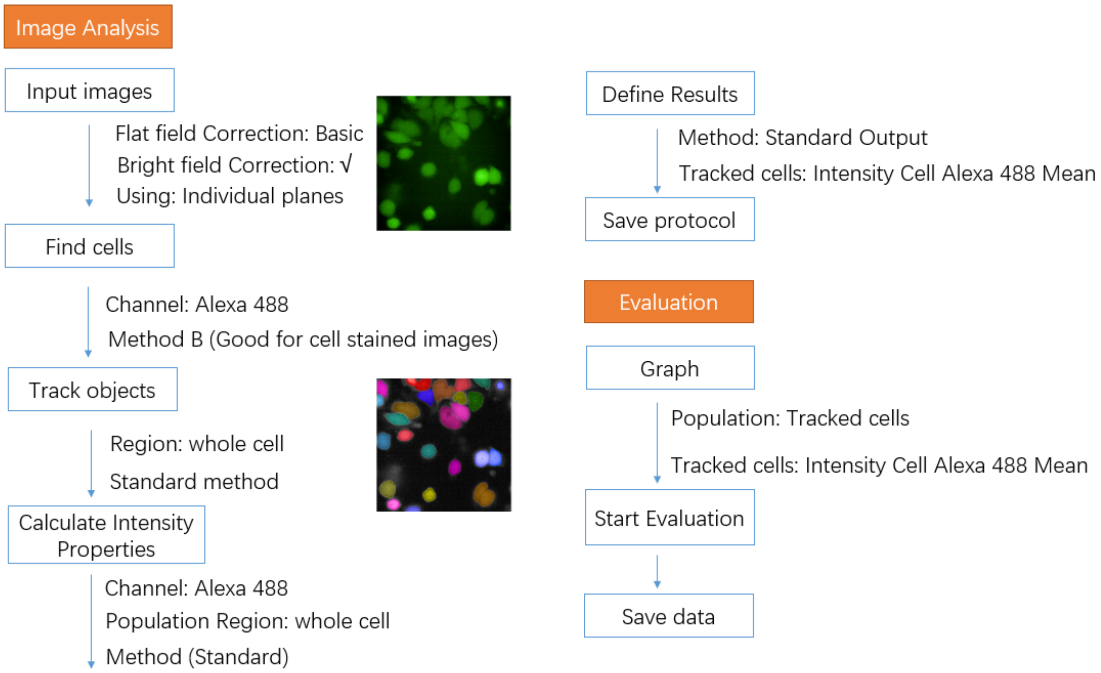

Supplementary Figure 48. The analysis flow diagram of the high throughput assay via Harmony software.

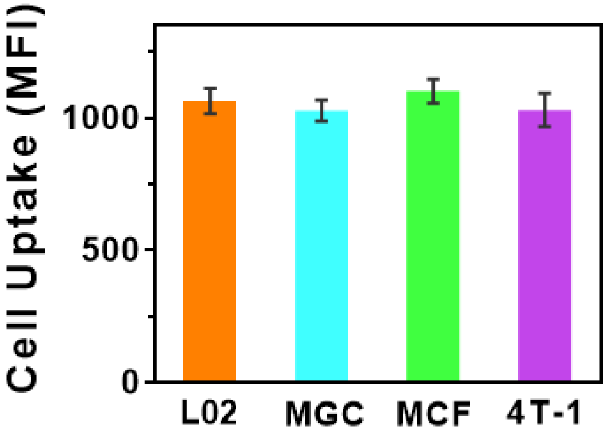

Supplementary Figure 49. Cellular uptake of the GOx-aZIF nanocomposite for different cell types. There was no significant difference in internalizing the nanocomposite among these non-phagocytes. Data were represented as mean ± SD (n=3 biologically independent samples). Source data are provided as a Source Data file.

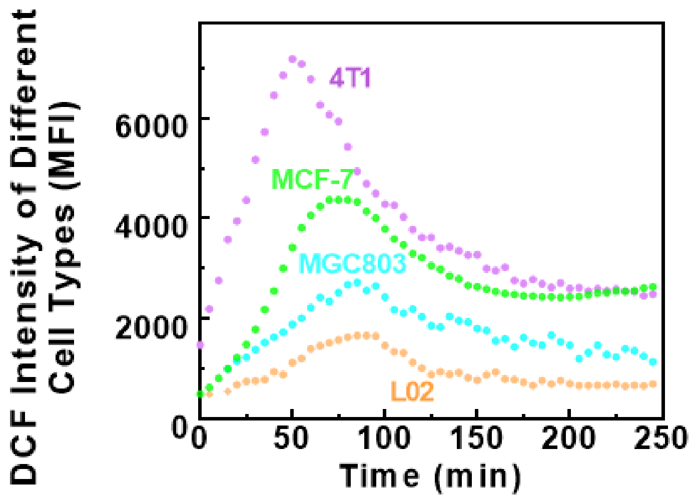

Supplementary Figure 50. The DCF fluorescence intensity of different cell types in the presence of GOx-aZIF. Every point in each curve represents the mean FI value that was calculated from the images of a total of ten different fields. Source data are provided as a Source Data file.

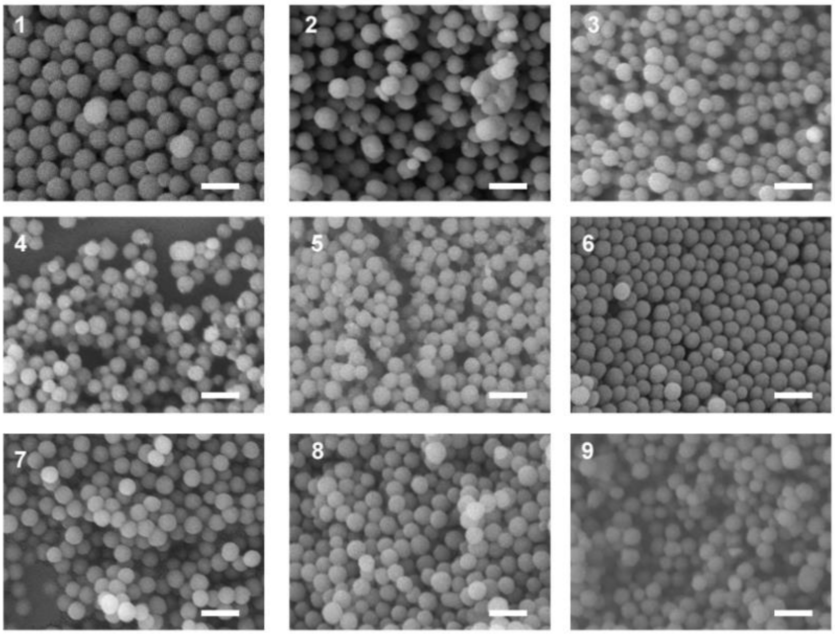

Supplementary Figure 51. SEM images showing morphology of aZIF synthesis from different batches. Scale bars are 200 nm.

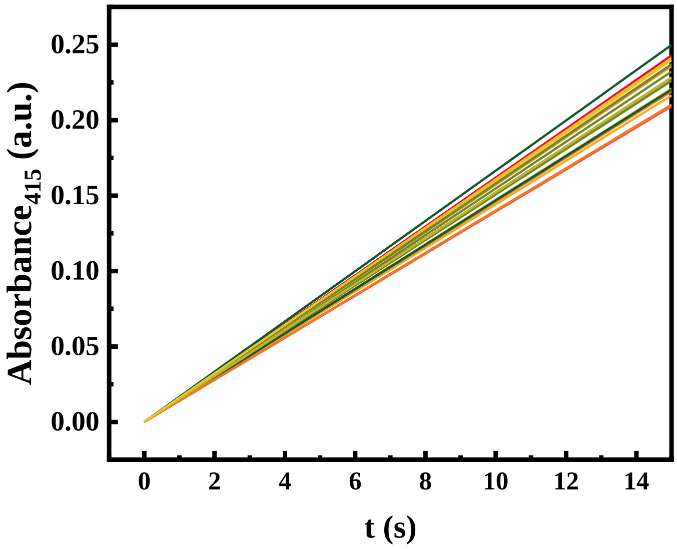

Supplementary Figure 52. Enzymatic reaction kinetics for the oxidation of glucose catalyzed by different batches of GOx-aZIF nanocomposites. Samples were obtained from 20 different batches. The slope represents the reaction rate catalyzed by GOx-aZIF nanocomposites, which is an indicator of enzymatic activity. Source data are provided as a Source Data file.

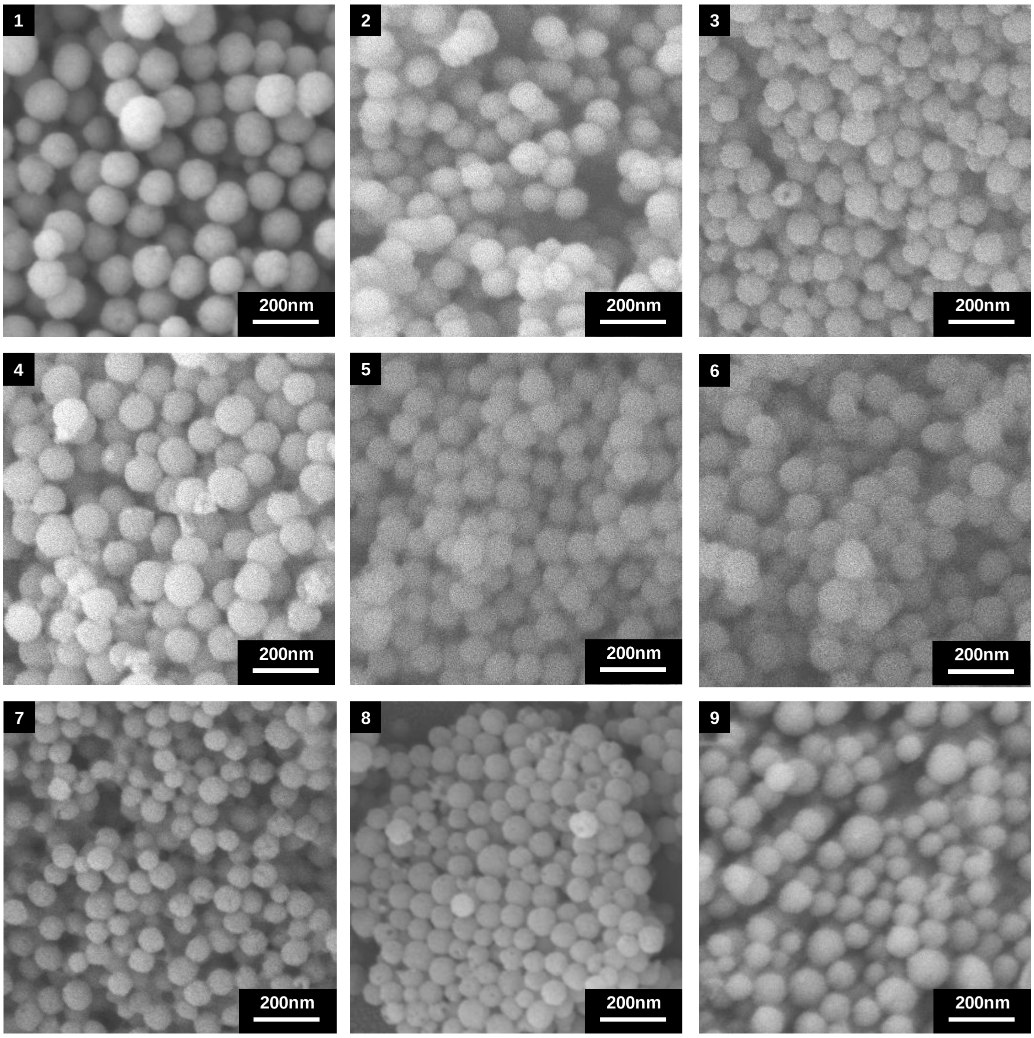

Supplementary Figure 53. SEM images showing morphology of GOx-aZIF synthesis from different batches. Scale bars are 200 nm.

Supplementary Tables

Supplementary Table 1. EXAFS data fitting results of aZIF and ZIF-8.

Samples	Shell	CN	R (Å)	$\sigma^2 \times 10^2$ (Å^2)	ΔE_0 (eV)
aZIF	Zn-N	3.8<br>(0.6)	1.99<br>(0.01)	0.49<br>(0.13)	6.1<br>(1.79)
ZIF-8	Zn-N	4.0	2.00<br>(0.02)	0.59<br>(0.07)	7.1<br>(1.04)

CN, the coordination number for the absorber-backscatterer pair.

R, the average absorber-backscatterer distance.

σ^2, the Debye-Waller factor.

ΔE_0, the inner potential correction.

The data range used for data fitting in k-space (Δk) and R-space (ΔR) are 2.5-13.1 Å^{-1} and1-4 Å.

Data fitting was carried out in Artemis program.

Supplementary Table 2. Element percentages of ZIF-8 and aZIF.

Materials	Zn (wt%)	N	N/Zn
aZIF	30.3	22	3.4
ZIF-8	28.5	24.5	4.0
ZIF-8 (theoretical value)	28.4	24.5	4.0

Supplementary Table 3. BET surface area and pore volume of ZIF-8, aZIF and GOx-aZIF nanocomposites.

Materials	BET surface area ($m^2\ g^{-1}$)	Pore Volume($cm^3\ g^{-1}$)
ZIF-8	1208	0.63
aZIF	96	0.356
GOx-aZIF	75	0.212
ZIF-8 reported[1]	1810	0.636

Supplementary Table 4. Levene's test and F-test K_m value for sample comparison.

F test	Degree of freedom	Sum of squares	Mean Square	F value	P value
Factor	1	0.0222	0.0222	0.0715	0.8024
Error	4	1.2415	0.3104		
Total	5	1.2637			

Levene's test using absolute deviation	Degree of freedom	Sum of squares	Mean square	F value	P value
Factor	1	0.1061	0.1061	1.9702	0.2331
Error	4	0.2154	0.05384		

Supplementary Table 5. Substrate size of different enzymes and enzyme activity.

Enzyme	Substrate	Substrate size	Enzyme activity
GOx	Glucose	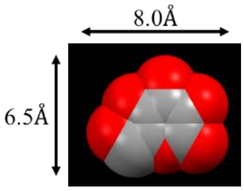	ca. 100 U mg^{-1}
CAT	Hydrogen peroxide (H_2O_2)	ca. 4.5 Å x 3.2 Å	ca. 2000 U mg^{-1}
CALB	P-Nitrophenyl Butyrate (*p*-NPB)	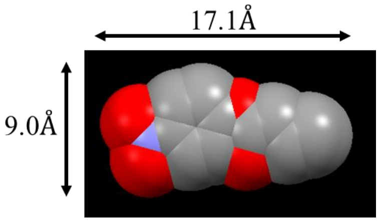	ca. 0.5 U mg^{-1}

Supplementary Table 6. N/Zn ratio of aZIF with varying reaction time.

Reaction Time	Zn (wt%)	N (wt%)	N/Zn
1h	31.9	21.1	3.1
2h	33.1	20.7	2.9
4h	33.5	20.4	2.8
6h	34.3	18.4	2.5

Supplementary Table 7. Technologies of detecting intracellular glucose.

Technology	Key point	Shortage
Förster resonance energy transfer (FRET)	Introduction of a fusion protein	Require a genetic engineering of cells[2]
Electrochemical sensors	Nanopipette puncture into cells	Destructive to cells[3]
Positron emission tomography (PET)	Fluorodeoxyglucose (FdG) as imaging agent	Limited resolution of millimeters[4]
Desorption electrospray ionization mass spectrometry imaging (DESI-MSI)	Label-free molecular imaging technique	Analysis on tissue level[5]

Supplementary Methods

Materials.

Zinc diacetate dihydrate, hydrogen peroxide (30wt%), 2-methylimidazle (2-MeIM), glucose oxidase (GOx) from *Aspergillus niger*, peroxidase from horseradish (HRP), catalase (CAT), *p*-nitrophenylpalmitate (*p*-NPB), 2,2'-azino-bis (3-ethylbenzothiazoline-6-sulfonic acid) diammonium salt (ABTS), 2-deoxy-2-[(7-nitro-2,1,3-benzoxadiazol-4-yl)amino]-D-glucose (2-NBDG), Rhodamine B isothiocyanate (RITC) and 2’,7’-dichlorodihydrofluorescein diacetate (DCFH-DA, with absorption/emission wavelengths at around 470-505/510-540 nm) were purchased from Sigma-Aldrich. Lyso-Tracker probe was purchased from Life Sciences.

Synthesis of amorphous ZIF and GOx-amorphous ZIF.

In a typical experiment, 1 mL of zinc acetate solution (20 mM) was added into 1 mL of 2-methylimidazole (80 mM), followed by stirring for 30 minutes in a 5 mL glass bottle. The synthesized aZIF was then centrifugated (20000 g, 5 minutes, ambient temperature), washed for 3 times with DI water and lyophilized. Due to the excess amount of 2-methylimidazole, the yield was calculated based on the conversion of zinc. The product was weighed to be 1.8 ± 0.1 mg with a yield of ca. 41% according to ICP-OES results.

The synthesis of GOx-aZIF was similar as that of aZIF. After adding zinc acetate solution to 2-methylimidazole, 80 μL, 0.5-5mg mL^{-1} GOx was immediately added to the reactor and stirred for 30 minutes, followed by the same washing and drying procedure. The weight of the product was 2.00 ± 0.1 mg. The yield was calculated to be ca. 45% according to ICP-OES results. The GOx in the supernatant was determined by Coomassie Bright Blue assay. Neither protein nor enzyme activity can be detected in the supernatant, which confirmed 100% encapsulation of GOx in aZIF.

Enzymatic activity assay of GOx-amorphous ZIF.

The activity of glucose oxidase (GOx) in aqueous solution was measured by using glucose as the substrate in phosphate buffer saline (pH 7.4). In a typical measurement, 50 μL of GOx-aZIF nanocomposites or free GOx with the same amount of protein was added in phosphate buffer saline containing glucose (100 mM), ABTS (0.28 mg mL^{-1}) and HRP (0.05 mg mL^{-1}). The increase of absorbance at 415 nm was measured by using a UV/Vis spectrophotometer. The Michaelis-Menten (MM) constant, K_m, was obtained by the non-linear fitting of initial reaction rate with substrate concentration according to the Michaelis-Menten equation.

Enzymatic activity assay of CALB-amorphous ZIF.

The hydrolytic activity of CALB/ZIF-8 was determined using *p*-nitrophenyl butyrate (*p*-NPB) as the substrate. Firstly, *p*-NPB was dissolved in acetone and then diluted with phosphate buffer solution (50 mM, pH 7.0) containing 1.25% (w/v) Triton X-100, with a final concentration of 0.5 mM. The reaction was initiated by adding 50 µL of enzyme solution (phosphate buffer, 50 mM, pH 7.0) to 950 µL of the substrate solution. The absorbance at 348 nm was recorded using a UV/Vis spectrophotometer.

Labelling GOx with Cy5.

The GOx was dissolved in phosphate buffer saline (10 mM, pH 7.4). Ten micrograms of Cy5 was added in GOx solution and stirred for 12 hours in dark. After reaction, the solution was then dialyzed against DI water to remove unreacted Cy5. The obtained GOx-Cy5 was utilized instead of GOx in the following synthesis.

HAADF-STEM and EDS analysis.

The aqueous suspension containing the amorphous ZIF or enzyme-amorphous ZIF nanocomposites was added on a micrograte and dried at room temperature. High-angle annular dark field (HAADF) images and energy dispersive spectrum (EDS) mapping were conducted at 300 kV on FEI Titan Cubed Themis 60-300 with double correctors and monochromator.

Electron microscopy of enzyme-amorphous ZIF nanocomposites.

1.6 mg GOx-aZIF nanocomposite was diluted, sonicated and resuspended thoroughly in 500 µL deionized water. For conventional imaging, the sample was added on a carbon grid and dried at room temperature. TEM images were taken on a JEOL JEM-2010 high-resolution microscope operating at an accelerating voltage of 120 kV. For Cryo-ET imaging of GOx-aZIF or crystalline ZIF-8, a 6-µL aliquot containing a 1:1 mixture of sample solution:gold fiducial beads (10 nm diameter, Aurion, The Netherlands) was applied onto a glow discharged copper grid coated with holey carbon (C-flat; Protochips, Raleigh, NC) prior to plunge-freezing. The grids were single-side blotted away from the sample side for 3.5 s, vitrified by plunge-freezing into liquid ethane using a Vitrobot Mark IV (Thermal Fisher Scientific).

The grids were screened using a Tecnai Arctica microscope operated at a voltage of 200 kV equipped with a Falcon III direct electron detector (Thermal Fisher Scientific) at a nominal magnification of 53,000×. Grids with continuous carbon and thin ice were subsequently imaged on a Titan Krios microscope operated at a voltage of 300 kV equipped with an energy filter (slit width 20 eV; GIF Quantum LS, Gatan) and K2 Summit direct electron detector (Gatan). Sample present in a single layer were recorded at a nominal magnification of 105,000x, resulting in a calibrated

pixel size of 1.34 Å. For tilt-series data collection, the stage was tilted from -60° to 60° at 2° step.

The electron beam induced motion was corrected by MotionCor[6] by averaging 16 frames for each tilt. Defocus of the tilt series were measured using CTFFIND4[7]. The tilt series were contrast transfer function corrected and tomograms were reconstructed by weighted back projection in IMOD[8]. Four times binned tomograms were intensity inverted and visualized in UCSF Chimera[9].

SEM analysis of amorphous ZIF and enzyme-amorphous ZIF.

Scanning electron microscope (SEM) images of samples were taken on a JSM 7401 field emission gun-scanning electron microscope at an accelerating voltage of 3.0-10.0 kV. The lyophilized powder of samples was firstly attached to a carbon paste and then sputter-coated with a thin layer of conductive gold to improve the electrical conductivity.

XRD analysis of amorphous ZIF and enzyme-amorphous ZIF.

Powder X-ray diffraction (XRD) patterns were recorded using a Bruker D8 Advance X-Ray diffractometer with a Cu Kα anode (λ= 0.15406 nm) at 40 kV and 40 mA, with step size of 2° min^{-1}.

Fourier Transform Infrared Spectrometer (FTIR)

The FTIR measurement was carried out on a Nicolet 6700 FTIR spectrometer in transmission mode at ambient temperature. The sample powder was mixed with KBr of spectroscopic purity, pressed to disc and exposed to FTIR analysis. 128 scans were performed with 1 cm^{-1} interval. Data was collected, averaged and smoothed.

Thermogravimetric analysis.

Thermal gravimetric analyses (TGA) in air were performed on a TA Instruments TGA 2050 Thermo gravimetric Analyzer. The sample was heated from room temperature to 600 ^{o}C at a rate of 20 ^{o}C min^{-1} under air atmosphere.

XPS measurement.

The X-ray photoelectron spectroscopy measurement (XPS) was conducted on Thermo Fisher ESCALAB 250Xi. The samples were synthesized, purified and lyophilized before applying the XPS test. The source gun type was be Al K Alpha. Energy step size was set to be 0.050 eV.

ICP-OES detection.

The induced coupled plasma optical emission spectrometer (ICP-OES) was conducted on IRIS Intrepid II (Thermo). Before ICP test, the samples were heated and then dissolved in *aqua regia* to form a solution.

N_2 sorption isotherms.

The nitrogen sorption measurements were conducted on Quantachrome Instrument at 77 K. The samples were degassed at 200 °C for 3h before test. The surface area was determined by the Brunauer-Emmett-Teller (BET) method. The pore size distribution was determined by Non-Local Density Functional Theory (NLDFT) method assuming a slit/cylinder pore. The range of relative pressure (P/P_0) for aZIF and GOx-aZIF was from 2.34e-5 to 9.9e-1, while for ZIF-8 was from 7.36e-6 to 9.00e-1. The BET surface area was determined using relative pressure (P/P_0) ranging from 9.67e-2 to 2.98e-1 for aZIF and GOx-aZIF, and from 5.01e-3 to 9.96e-2 for crystalline ZIF-8.

DLS analysis.

Dynamic light scattering (DLS) was performed on Malvern ZetaSizer Nano ZS90. The samples (aZIF, ZIF-8, GOx-aZIF) were synthesized, centrifugated and washed for three times. The precipitates were then resuspended in phosphate buffer saline (10 mM, pH 7.4). The mass concentration of the precipitates in solution was 0.5-1 mg mL^{-1}. Before test, the samples were sonicated for 1 minute. During one test, 800 μL solution was added to a cuvette. The temperature was at 25 °C. The detection angle was set to 90 degree.

EXAFS data collection and analysis of samples.

The X-ray absorption at the Zn K-edge of samples was recorded at room temperature in transmission mode with ion chambers at beamline BL14W1 of the Shanghai Synchrotron Radiation Facility (SSRF), China. The photon energy was calibrated with the first inflection point of Zn K-edge in zinc metal foil. Data analysis was performed using the software Athena. Data was processed by removing the smooth background from the measured absorption coefficient $\mu(E)$ vs E data, normalizing the X-ray absorption coefficient, converting the $\mu(E)$ to $\chi(k)$ and Fourier transforming the resultant spectrum from k-space to R-space. The EXAFS data fitting was performed in the Artemis program using theoretical models from the ZIF-8 crystallographic data.

Synchrotron radiation X-ray pair distribution function.

The set-up of a synchrotron radiation X-ray pair distribution function (PDF) experiment was shown in Supplementary Figure 11. Data was collected by loading grinded samples into a 1.0 mm diameter silica capillary with a collection time of 10 min on a Mar 3450 detector with a small lead shielding plate sticked. The energy of the X-ray is ~ 70 keV and the distance between the sample and detector was 26.5 cm. Experimental data was integrated and converted by using Fit2D[10] and further transferred to a program PDFGETX3[11] to produce total scattering factor, S(Q) and pair distribution function, G(r) with Q_{max} of 13.5 angstrom^{-1}. No difference was observed in the short-range order (below 6 angstrom) between aZIF and crystalline ZIF-8 (Figure 2e, f), which was also demonstrated by XAFS results. For the crystalline ZIF-8, a significant ordered distribution up to 15 angstrom was observed. In contrast, a featureless distribution was found in the aZIF, demonstrating the loss of medium to long-range order.

Confocal laser scanning microscopy.

The Confocal Laser Scanning Microscopy (CLSM) was performed on a FV1200 Confocal/FLIM/FCS Olympus. 50 μL 2-NBDG was incubated with 0.2mL GOx-aZIF or GOx-ZIF-8 of the same concentration (c.a. 1.6mg mL^{-1}) for 10 minutes under the same conditions followed by centrifugation. The precipitates were then washed 3 times using deionized water (DI) in order to remove the 2-NBDG adsorbed on the surface of the particle. Before conduction the CLSM, the precipitates were resuspended to 1.6 mg mL^{-1}. The excitation and emission wavelength were respectively 488nm and 525nm for 2-NBDG. The A1 N-SIM STORM system was equipped with Nikon A1 plus camera. The laser power for FITC channel of confocal microscopy was 80 mW, and 7.5% power was set to avoid saturated pixels. The pinholes were 35.8 μm.

Flow cytometry.

Flow cytometry was carried out on a CytoFLEX LX Flow Cytometer. The fluorescent 2-NBDG were respectively incubated with GOx-aZIF and GOx-ZIF-8 for 2, 4, 6, 8, 10 minutes. The concentration of GOx-aZIF and GOx-ZIF-8 was both 1.6mg mL^{-1}. The particles were subsequently centrifugated and washed for 3 times. Before the flow cytometry, the precipitates were resuspended to 1.6mg mL^{-1} including a blank GOx-aZIF and GOx-ZIF-8 sample without 2-NBDG. The excitation wavelength and emission wavelength for 2-NBDG were set to be 488 nm, 525 nm, respectively. CytoFLEX LX Flow Cytometer was equipped with 6 laser devices (488 nm, 638 nm, 405 nm, 561 nm, 355 nm, 808nm, whose power was 50 mW, 50 mW, 80 mW, 30 mW, 20 mW, 60mW respectively.) The sheath fluid was deionized water. The threshold for Violet SSC channel was set to 750. The sample input rate was ca. 1500 particles per second. Each sample would collect no less than 10000 events to display.

Determination of the 2-NBDG loading efficiency.

GOx-aZIF or GOx-ZIF-8 was firstly incubated with 2-NBDG methanol solution and then centrifugated. The supernatant was collected, and its absorbance spectrum was measured and compared with the 2-NBDG solution before incubation (Supplementary Figure 18c, d). The loading efficiency of 2-NBDG was estimated to be 0.119 ± 0.002 μg mg^{-1} for GOx-aZIF, and 0.045 ± 0.004 μg mg^{-1} for GOx-ZIF-8.

Molecular simulations.

The all-atom model of ZIF-8 was constructed based on the structure of unit cell reported in CCDC (ID 864311, http://www.ccdc.cam.ac.uk) and ChemTube3D (http://www.chemtube3d.com/). For 2-MeIM, its topology was generated by the PRODRG (http://davapc1.bioch.dundee.ac.uk/cgi-bin/prodrg). Its charge group and charge distribution were defined based on the parameters from quantum chemistry calculations. Then a cubic simulation box of ZIF-8 was constructed with a side length of 15.2937 nm, which contains 8748 Zn^{2+} and 17496 2-MeIM (one hydrogen lost). To construct the simulation box of aZIF, 2700 2-MeIM were randomly selected and deleted according to the experimental molar ratio of Zn^{2+} to 2-MeIM. The system was neutralized by 2700 Cl^{-} as counter ions.

After energy minimization, MD simulations with a simulated annealing protocol were performed using Gromacs 5.1.4 package[12] (http://www.gromacs.org/) and Gromos96 43a1 force field[13]. A 2 fs time step was used to integrate the equations of motion. The cut-off of the Lennard-Jones (LJ) potential was set to 1.2 nm. Standard PME function was used to deal with the Coulomb potential. Periodic boundary was used in the x, y and z directions. Neighbor list was updated every 10 steps. The initial system temperature was set to 298.15 K. The initial velocities of beads were generated according to the Maxwell distribution at system

temperature. Then, annealing protocol was applied. The system temperature was controlled to linearly increase from initial 298.15 K to 800 K at 20 ns, followed by a linear decrease to 298.15 K at 80 ns. Then 20 ns MD simulations in the NVT ensemble at 298.15 K were performed obtain the equilibrated structure for analysis. Four independent simulations were performed for each set of conditions. The snapshots were prepared using Rasmol[14] (http://rasmol.org). Radial distribution function (RDF) between each atom pair in aZIF or ZIF-8 was calculated to clarify the difference in the coordination state of Zn and the change of atomic structure, where $g_{ij}(r)$ of each atom pair between C and N of 2-MeIM, and Zn was calculated using auxiliary program rdf provided by Gromacs.

Cell culture.

The human gastric cancer cell MGC803, human breast cancer cell MCF7, and hepatic cancer cell $HepG_2$ were maintained in DMEM medium containing 10% fetal bovine serum (FBS). Mouse breast cancer cell 4T1 and Human normal hepatocyte L02 were maintained in PRIM1640 medium containing 10% fetal bovine serum (FBS). All cells were incubated at 37 °C in an atmosphere of 5% $CO_{2.}$ MCF-7 and 4T1 were from the ATCC (American Type Culture Collection). MGC803 was from the China Infrastructure of Cell Line Resource. L02 was from the Cell bank of Type Culture Collection of the Chinese Academy of Sciences.

Glucose concentrations in different types of cells.

The determination of glucose concentration in cells was performed using enzymatic cascade. In a typical assay, cells are digested by trypsin. Cell suspension was then washed 3 times by PBS in order to remove the glucose in cell culture. Cells were resuspended and counted by handheld automated cell counter (MILLIPORE Scepter Cell Counter). Afterwards, cell disruption was implemented by drastic supersonic in ice-water bath. Different volumes of supernatant were added to a 96-well plate and PBS was then added to achieve the same volume. Solutions containing different concentrations of glucose were also added to the 96-well plate to obtain the calibration curve. After that, PBS containing GOx, HRP as well as ABTS was added. The absorbance at 415 nm was measured on a microplate reader (Tecan Infinite 200 Pro).

Detection of glucose concentration in cell supernatant.

Cells were firstly disrupted and centrifugated. The supernatant containing glucose was added by GOx-aZIF nanocomposites or free GOx (both were treated by trypsin digestion for 3 hours at 35 °C) with CALB (enzyme used to active DCFH-DA outside cells) and DCFH-DA. A blank experiment was implemented without adding GOx-aZIF or free GOx.

Cell uptake assay.

Cells were seeded into the 96-well plates for 24 h, and then be refreshed with glucose-free culture for three times. Once the cells were added with RITC-labelled GOx nanocomposites, the red fluorescent intensity (excited at 561 nm) and corresponding images of each cell were immediately recorded using a Perking Elmer high content screening system (HCS) “Operetta”. The peak intensity of different cell types, which showed the cellular uptake capacity for the nanocomposite was analyzed by using an integrated image analysis software “Harmony”.

Intracellular trafficking analysis.

Cells were seeded into the petri dish for 24 h, and then be refreshed with glucose-free culture for three times. Following that, the RITC labeled GOx nanocomposites were added to the cells and co-incubated for 1 h. Then, the medium was refreshed, and Lyso-Tracker Green probe was added for 30 min at 37 °C. Finally, the fluorescent images were captured by using a structured illumination super-resolution microscope (SIM).

Intracellular detection of glucose.

The intracellular glucose was dynamically monitored by using a •OH-sensitive DCFH-DA dye, which could be de-esterified intracellularly to form DCFH. DCFH could react with H_2O_2 (the product of glucose conversion catalyzed by GOx) and produce highly fluorescent DCF with excitation at 488 nm. Cells were primarily allowed to adhere for 24 h in 96-well plates and be washed for three times with glucose-free PBS prior to the detection. Subsequently, the DCFH-DA dye (at a work concentration of 10 μM) and the GOx-aZIF or GOx-ZIF-8 at certain concentrations were simultaneously added into the cells for dynamic detection (4.5 μg mL^{-1}, 15 μg mL^{-1}, 45 μg mL^{-1}). The fluorescent images of the cells were recorded during 4-h incubation period (37 °C and 5% CO_2) via the "Operetta CLS" High Content System (Perkin Elmer). Alex 488 channel (LED power) was selected to acquire the fluorescent signal from DCF, which was excited at 460-490 nm and recorded at 500-550 nm emission wavelength via standard filter sets. 10% power was set for the excitation, and 10 ms exposure time was controlled to avoid saturated pixels. Particularly, the instrument was equipped with a 16-bit sCMOS camera, which operated in a fast acquisition mode for exposure time ≤ 10 ms. The pinhole size was 55 μm.

To obtain the intensity values of the cells, all images of each cell types were quantified with the Harmony analysis software (PerkinElmer).

With the assist of the “Harmony” software, the MFI data were acquired and analyzed by subtracting the MFI data of DCF of control cell sample in the absence of enzyme. A typical analysis flow diagram has been added as Supplementary Figure 48. By selecting a Standard Method “Calculate Intensity Properties” of Harmony, the Mean value and the standard deviation that characterizing an intensity histogram were calculated. Particularly, the images in duplicated wells with a total of ten different fields were captured regarding the assays for enzyme concentrations and cell types. All the images were adjusted at a same contrast via “Image Channels” in “Harmony”. Data in the calibration plot were presented as mean ± SD. Corresponding graph was one representative image for each group.

Supplementary Notes

Supplementary Note 1

The bands at 3131 and 2929 cm^{-1} were attributed to the aromatic and aliphatic C-H stretch of the imidazole, respectively. Peak at 1584 cm^{-1} was assigned to the C=N stretch mode, while bands at 1350-1500 cm^{-1} corresponded to the entire ring stretching. The bands at 900-1350 cm^{-1} were attributed to the in-plane bending of the ring. The peak at 1147 cm^{-1} was assigned to the aromatic C-N stretching mode. Peaks at 996 and 761 cm^{-1} came from the C-N bending vibration and C-H stretching mode. Bands below 800 cm^{-1} were assigned as out-plane bending. The peak at 421 cm^{-1} was the characteristic Zn-N stretching mode, indicating that zinc ions coordinated with the nitrogen atoms. The characteristic absorption bands at 1640 to 1660 cm^{-1} were the typical stretching mode from carbonyl group of amino acid from protein, thus confirming the presence of GOx.

Supplementary Note 2

In a typical synthesis, 1 mL of zinc acetate (20 mM) was mixed with 1 mL of 2-methlyimidaozle (80 mM) and 0.08 mL of GOx (2.5mg mL^{-1}). Due to the excess of 2-methylimidaozle, the theoretical yield was calculated on the conversion of zinc. By ICP-OES, the yield was determined to be ca. 40%. All the GOx molecules were encapsulated in aZIF, which was confirmed by Bright Coomassie Assay and enzyme activity test in the supernatant. The GOx-aZIF collected was ca. 2.0±0.1 mg, of which ca. 0.2 mg was GOx. Therefore, the weight percentage of GOx in the composite was consequently ca. 10%, which agreed well with the TGA result.

The decomposition of crystalline ZIF-8 began at around 400 °C, while the decomposition temperature of amorphous ZIF started at around 300 °C, showing obviously lower thermal stability. This is in consistent with a previous report[15], which showed that when the crystalline MOFs changed to amorphous ones gradually by ball milling, the stability of MOFs decreased accordingly.

Supplementary Note 3

The superimposition of ZIF-8 structure obtained from simulation on the crystallographic structure was obtained using auxiliary program provided by Gromacs (Supplementary Figure 12a). XRD spectrum of ZIF-8 obtained from the simulation model was obtained using PowderCell (http://www.ccp14.ac.uk/tutorial/powdcell/). All the main peaks obtained from experimental results can be repeated in the simulations (Supplementary Figure 12b). However, more peaks appear in simulation because of the thermal motion of atoms. For further confirmation, restriction was applied on the atoms to examine the change of XRD (Supplementary Figure 12c). It can be seen that more similar curve to the one of crystallographic structure was obtained under stronger restriction, confirming that more peaks appear due to the thermal motion of atoms. Thus, in consideration of thermal motion, XRD spectrum from simulation model agreed well with the experimental one, which demonstrates the accuracy of the simulation model.

Supplementary Note 4

Please note that the Fourier Transformation of linear electron density did not directly show the pore size of the cavity in crystalline ZIF-8 or amorphous ZIF. However, it represents the variation of electron density along the line. Since the cavity shows lower electron density and the structure shows higher electron density (after inversion), the less frequently changed electron density reflects larger pore size while more frequently changed one would represent a smaller pore size.

Supplementary Note 5

The F-test was employed to statistically determine whether there is significant difference between K_m value of GOx and GOx-aZIF. Levene's test using absolute deviation was conducted so that the homogeneity of variance was ensured for these groups of K_m data. For F-test, the null hypothesis H_0 is that there is no significant difference between K_m values of two groups of data. The level of significance α was set to 5%. The Levene's test and F-test results were displayed in Supplementary Table 4.

According to the Levene's test, the homogeneity of variance could be assured. F-test results accepts the null hypothesis, showing that there is no significant difference of K_m value between GOx and GOx-aZIF, and thus indicating a similar affinity toward substrate glucose.

Supplementary Note 6

During the activity test, it is generally accepted that the substrate would firstly diffuse inside MOFs, and then the enzyme reaction would occur when the substrate reaches the active site of the encapsulated enzyme. Therefore, both the diffusion of substrates and the intrinsic enzymatic reaction kinetics need to be taken into consideration to explain the different apparent activities between enzyme-aZIF and enzyme-ZIF-8. The apparent activity of GOx-ZIF-8 was majorly restricted by the small diffusion rate of substrate in ZIF-8, instead of the intrinsic enzymatic reaction kinetics. Therefore, the apparent activity of GOx-ZIF-8 (only 5%) was much lower than free enzyme. In contrast, the diffusion rate of substrate in amorphous ZIF with larger pores became faster. The substrate diffusion was not the major restriction in this case. The apparent activity of GOx-aZIF was only restricted by the intrinsic enzymatic reaction kinetics, thus displaying similar activity (~100%) as free GOx.

In the case of CAT, the substrate hydrogen peroxide has smaller molecular size than glucose and thus faster diffusion rate in ZIF-8. Therefore, the relative activity of CAT-ZIF-8 (25%) was higher compared to that case of GOx-ZIF-8 (5%). For CAT-aZIF, the apparent activity was only restricted by the intrinsic enzymatic reaction kinetics, thus displaying similar activity (~100%) as free CAT. As a result, the activity enhancement of CAT was only 5 times.

In the case of CALB, the substrate p-NPB has larger size than glucose, and CALB has lower intrinsic activity than GOx. Therefore, the apparent activity of CALB-ZIF-8 was restricted by both the diffusion and the enzyme reaction. Thus, the relative activity of CALB-ZIF-8 (20%) was higher compared to that case of GOx-ZIF-8 (5%). For CALB-aZIF, the apparent activity of CALB-aZIF was restricted predominantly by the intrinsic enzymatic reaction kinetics, thus displaying similar activity (~100%) as free CALB. As a result, the activity enhancement of CALB was only 5 times. To verify the hypothesis above, we tried to establish a diffusion-reaction model as follows.

The model is Maxwell-Stephan diffusion model. Considering aZIF having mesopores of ca 2-10 nm as observed under cryo-ET, the diffusion of substrates in aZIF could be depicted using substrate diffusion model in porous media which involved the bulk diffusion as well as the Knudson diffusion. The pore size of microporous ZIF-8 was similar to the molecular sizes of substrates, and thus the surface diffusion was predominant in this situation. To simplify the model, some general hypothesis was stated here: 1) the nanoparticles (both aZIF and ZIF-8) were regarded as spheres with identical radius (50 nm); 2) the encapsulated enzymes in the nanoparticle agglomerated as a sphere in the center of the particle.

The substrate diffusion model in porous media for enzyme-aZIF was constructed as follows.

Generally, the Maxwell-Stephan(M-S) equation was written as:

$$-\mathbf{d}_i = \sum_{\substack{j=1 \\ j\neq i}}^{n} \frac{x_i\mathbf{N}_j - x_j\mathbf{N}_i}{C_t Đ_{ij}}, i = 1,2,\dots,n \qquad (1)$$

,

where $Đ_{ij}$ denotes the Maxwell-Stephan (M-S) diffusivity between component i and j, $\mathbf{N}_i$ is the molar flux of species i, and $\mathbf{d}_i$ represents the diffusion driving force of species i.

In porous media, the M-S equation[16] was derived as

$$-C_t \nabla x_i = \sum_{\substack{j=1 \\ j\neq i}}^{n} \frac{x_j\mathbf{N}_i^d - x_i\mathbf{N}_j^d}{Đ_{ij}^e} + \frac{\mathbf{N}_i^d}{Đ_{iM}^e}, i = 1,2,\dots,n \qquad (2)$$

,

in which $Đ_{iM}^e$ portrays the effective interaction between component i with the porous media, reflecting the Knudson diffusion, the viscous flow as well as the surface diffusion. In the case of diffusion in aZIF, the Knudson diffusion was predominant. The relation between M-S diffusivity and effective M-S diffusivity $Đ_{ij}^e$ could be described using the following equation:

$$Đ_{ij}^e = \frac{\varepsilon}{\tau} Đ_{ij} \qquad (3)$$

In Supplementary Equation 3, the ε is the void fraction of the media, while τ depicts the tortuosity of the pores. As for binary mixture (glucose aqueous solution), we define species 1 as water, and species 2 as glucose. For equimolar diffusion, we have

$$\mathbf{N}_1^d + \mathbf{N}_2^d = \mathbf{0}, \tag{4}$$

and for binary mixtures, we have

$$x_1 + x_2 = 1. \tag{5}$$

Combining Supplementary Equations 2, 4and 5, the M-S equation for component 2 could be written as

$$\nabla c_2 = -\left(\frac{1}{Ð_{12}^e} + \frac{1}{Ð_{2M}^e}\right)\mathbf{N}_2^d \tag{6}$$

Based on Supplementary Equation 6, we could obtain the conservation equation for glucose in spherical coordinates as

$$\frac{\partial c_2}{\partial t} = \frac{1}{r}\frac{\partial\left(rD_2^e(\partial c_2/\partial r)\right)}{\partial r} \tag{7}$$

Where the $Ð_2^e$ is defined as

$$\frac{1}{Ð_2^e} = \left(\frac{1}{Ð_{12}^e} + \frac{1}{Ð_{2M}^e}\right). \tag{8}$$

The boundary condition of partial differential equation (PDE) (Supplementary Equation 7) is defined as:

$$c_2(r_{np}, t) = c_{2,bulk} \tag{9}$$

$$Ð_2^e \nabla c_2(r_{en}, t) = R_S, \tag{10}$$

where r_{np} is the radius of the particle, r_{en} is the radius of agglomerated enzyme clusters, and R_S is the reaction rate of enzymes. By applying the Michaelis-Menten equation, the enzyme reaction rate R_S could be obtained as:

$$R_S = -\frac{k_{cat}c_2 n_{en}^S}{K_M + c_2}, \tag{11}$$

Finally, the initial condition of Supplementary Equation 7 is

$$c_2(r, 0) = 0 \tag{12}$$

The model of surface diffusion for enzyme-ZIF-8 are built up as follows. The fraction occupancy, θ_i is commonly used in surface diffusion, and can be defined as

$$\theta_i = \frac{c_i}{c_{i,sat}} \tag{13}$$

The $c_{i,sat}$ is the thermodynamic parameter that depicts the saturated surface concentration. Thus, the M-S equation for surface diffusion becomes

$$-\frac{\theta_i}{RT}\nabla\mu_i = \sum_{\substack{j=1 \\ j\neq i}}^{n} \frac{1}{Đ_{ij}^S}\left(\frac{\theta_j \mathbf{N}_i}{c_{i,sat}} - \frac{\theta_i \mathbf{N}_j}{c_{j,sat}}\right) + \frac{\mathbf{N}_i}{c_{i,sat}Đ_{iV}^S}, i = 1,2, \dots, n \tag{14}$$

In Supplementary Equation 14, the $Đ_{iV}^S$ is the surface M-S diffusivity of species i, and the $\nabla\mu_i$ is the gradient of chemical potential, which could be written as,

$$\frac{\theta_i}{RT}\nabla_{T,P}\mu_i^S = \sum_{j=1}^{n} \Gamma_{ij}^S \nabla\theta_j \tag{15}$$

Where

$$\Gamma_{ij}^S \equiv \theta_i \frac{\partial \ln a_i^*}{\partial \theta_j} \tag{16}$$

By applying the Langmuir adsorption model, fraction occupancy could be written as

$$\theta_i = \frac{b_i c_{i,sat}}{1 + \sum_{j=1}^{n} b_j c_{j,sat}} \tag{17}$$

Derived from Supplementary Equations 16, 17, for dilute solution

(substrate aqueous solution in this case), the thermodynamic factor Γ_{ij}^{S} could be written as

$$\Gamma_{ij}^{S} = \delta_{ij} + \frac{\theta_i}{\theta_V}, \quad (18)$$

We consider there is no 'reverse exchange', that is to say, the 'Single File Diffusion' (SFD) situation. Hence, the conservation equation is

$$\frac{\partial \theta}{\partial t} = \nabla \cdot \left([D^S](\nabla \theta)\right) \quad (19)$$

For a binary mixture model (species 1 as water, species 2 as substrate), $[D^S]$ is written as follow:

$$[D^S] = \begin{bmatrix} Đ_{1V}^{S} & 0 \\ 0 & Đ_{2V}^{S} \end{bmatrix} \begin{bmatrix} 1-\theta_2 & \theta_1 \\ \theta_2 & 1-\theta_1 \end{bmatrix} / (1-\theta_1-\theta_2) \quad (20)$$

Similar as the dusty gas model, the boundary condition for PDE (Supplementary Equation 19) for binary mixtures could be written as

$$\theta_i(r_{np}, t) = \frac{c_{i,bulk}}{c_{i,sat}} \quad (21)$$

$$[D^S]\nabla\theta(r_{en}, t) = \begin{bmatrix} 0 \\ R_{S2} \end{bmatrix} \quad (22)$$

Where

$$R_{S2} = \frac{k_{cat}[S]n_{en}^{S}}{K_M + [S]} \quad (23)$$

Here, the substrate concentration could be calculated using

$$[S] = \theta_2(r_{en}, t)c_{2,sat} \quad (24)$$

As for the initial condition, there are

$$\theta_1(r, 0) = \theta_{10} \quad (25)$$

$\theta_2(r, 0) = 0$ (26)

These two models involving the PDE could be solved by MATLAB (2014b). The parameters of enzymes were obtained by experiments in our laboratory. The order of M-S diffusivity was estimated according to work of other colleagues[17]. The results (Supplementary Figure 26) showed that, for GOx-aZIF, the glucose concentration inside aZIF reached almost the same as bulk solution in several microseconds, and thus the coupling diffusion-reaction rate was majorly restricted by enzyme reaction rate. In the case of GOx-ZIF-8, the glucose concentration remained null at enzyme clusters, indicating that the substrate was consumed before it could accumulate. In this case, the diffusion-reaction rate was restricted by diffusion rate and consequently the apparent enzyme reaction rate would be significantly reduced.

The concentration of hydrogen peroxide at enzyme clusters reached a plateau (~100%) for CAT-aZIF, while for CAT-ZIF-8 it increased to 20% of bulk solution concentration and remained constant. This is because the diffusivity of hydrogen peroxide was significantly larger than that of glucose, and thus the diffusion rate of CAT-ZIF-8 augmented. In this case, the enzyme reaction was affected by both diffusion and reaction process. As for CALB-aZIF and CALB-ZIF-8, the catalytic rate of CALB was considerably slower than GOx and CAT (Supplementary Table 5). As

shown in the Supplementary Figure 26, due to the significantly reduced enzyme activity, although the diffusion rate slowed down because of larger substrate size, the substrate could accumulate in CALB-ZIF-8. Hence, the enzyme reaction for CALB-ZIF-8 was also influenced by both diffusion and reaction process.

To sum up, the catalytic process of GOx-ZIF-8 was restricted by diffusion rate, while GOx-aZIF could reached the same substrate concentration as bulk solution. In consequent, GOx-aZIF showed great enhancement of enzyme activity than GOx-ZIF-8. In the case of CAT-ZIF-8 and CALB-ZIF-8, the hydrogen peroxidase diffused faster and CALB catalyzed slower. The substrate was able to accumulate inside CAT-ZIF-8 and CALB-ZIF-8, and the enzymes could sense a higher substrate concentration, resulting in higher relative activity of crystalline composites and therefore less enhancement of enzyme activity for amorphous composites for CAT and CALB.

Supplementary Note 7

To investigate the structure evolution of aZIF, samples with different synthesis time (1h, 2h, 4h, 8h, 12h, 24h) were taken out, followed by the centrifugation and washing procedure, and then characterized by XRD. As shown in Supplementary Figure 27, when the synthesis time was less than 2h, no obvious Bragg diffraction peaks was observed. When synthesis time was longer than 4h, the amorphous composite gradually transformed into crystalline one. This result indicates that the synthesis of amorphous structure in our study is time dependent, and the well control of reaction time results in the formation of amorphous structures.

Supplementary Note 8

We prolonged the reaction times to 1h, 2h, 4h, 6h respectively, and checked the morphology using SEM and N/Zn ratio using ICP-OES (for Zn) and elemental analysis (for C, H, O). The N/Zn ratio was listed in Supplementary Table 6. The N/Zn ratio decreased as the reaction became longer, which meant a more defective structure. According to the SEM images (Supplementary Figure 28), the nanoparticles remained its spherical structure before 1 hour. Subsequently, the nanoparticles aggregated to form square structure. This square structure became larger when the reaction proceeded. After 6 hours, the large square particles became a lamellar structure. The XRD results (Supplementary Figure 27) further confirmed the formation of crystalline structure when we extended the reaction time.

Supplementary Note 9

GOx-aZIF composites with different ligand concentrations were prepared (10 mM, 20 mM, 40 mM, 80 mM, 160 mM, 320 mM, 640 mM). As shown in Supplementary Figure 29, when the concentration of 2-methylimidaozle was less than 40 mM, the encapsulated GOx retained almost 100% of its original activity. Further increase of ligand concentration led to decreased activity retention. When the ligand concentration increased from 40 mM to 80 mM, activity decreased from ~100% to ~50%. When the ligand concentration was increased to 320 and 640 mM, the activity of encapsulated GOx further decreased to 25% and 5%. SEM images of aZIF prepared with difference ligand concentrations were given in Supplementary Figure 30, which indicated that the ligand concentration also affected the morphology. When the ligand concentration was less than 20 mM, no regular shape was observed. In the range of 40 mM to 80 mM, aZIF exhibited as nanospheres with some aggregation. When the ligand concentration was further increased, particles with dodecahedron morphology were obtained. This result was in line with XRD result (Supplementary Figure 31) which showed that characteristic XRD peaks assigned to ZIF-8 appeared and strengthened with the ligand concentration increased.

Supplementary Note 10

As can be seen in Supplementary Figures 32, 33, sonicating, shaking, incubation at high temperature and freeze-thawing cycles would not alter the morphology of GOx-aZIF. Moreover, activity retention of GOx-aZIF was almost 100% under these conditions, similar to GOx-ZIF-8 (Supplementary Figure 34).

Thermal stability of free GOx, GOx-aZIF and GOx-ZIF-8 was determined by incubating samples at 55 °C and 80 °C in aqueous solution for 1h. As can be seen in Supplementary Figure 35a, at 55 °C, free GOx retained less than 40% of original activity, while GOx-aZIF and GOx-ZIF-8 retained ~65% and ~80% of original activity, respectively. Similar result was obtained at 80 °C. Thermal deactivation kinetics (55 °C) was also investigated and an exponential decay model ($y=A_0e^{-\frac{t}{\tau}}$) could be used to depict the process (Supplementary Figure 35b). The time constant τ for free GOx, GOx-aZIF and GOx-ZIF-8 was 1.2 hours, 2.6 hours and 3.9 hours, respectively, indicating that GOx-aZIF would deactivate slower than free GOx and faster than GOx-ZIF-8.

The pH stability of GOx-aZIF was tested by incubating GOx-aZIF in acetate buffer (10 mM, pH 5.0), HEPEs buffer (10 mM, pH 8.0) and NaOH solution of pH 12.0. After incubation for 5min and 15 min, samples were taken out and followed by centrifugation and washed by DI water for 2 times and then exposed to SEM observation. As can been seen

in Supplementary Figure 36, after incubation in acetate buffer for 15min, obvious collapse of GOx-aZIF can be observed. Cohesion of cracked nanospheres appeared. While in alkaline HEPEs buffer and in NaOH solution, morphology of GOx-aZIF remained intact even after incubation for 15 min. The activity retention of GOx-aZIF in alkaline solution was comparable with GOx-ZIF-8 (Supplementary Figure 37).

The reusability of GOx-aZIF was tested for 9 cycles with triplicates. For each cycle, GOx-aZIF precipitates were resuspended and incubated with glucose solution (5 mM) for 1 minute. Please note here in our study the substrate glucose solution was utilized to ensure the measurement of the real reusability of GOx-aZIF in the practical catalytic environment. The catalysis of glucose by GOx will generate H_2O_2, which might cause the deactivation of enzyme. After incubation, 1 mL of solution was taken from solution and centrifugated. The GOx-aZIF precipitate was resuspended with deionized water (DI) and subjected to the standard enzyme activity assay. Then, the GOx-aZIF in glucose solution was centrifugated, resuspended, incubated and subjected to activity assay for next cycle. Similar procedure was applied for detection of GOx-ZIF-8 reuse performance. The experiment was replicated for three times.

GOx-aZIF retained ~50% of activity even after 9 cycles (Supplementary Figure 38a). After 5 cycles, GOx-aZIF retained more than 80% of initial activity (Supplementary Figure 38a) with

morphologies almost unchanged (Supplementary Figure 39). For GOx-ZIF-8, ~50% of initial activity was retained after 5 cycles. When setting activity of equivalent amount of free GOx as the 100% reference, after 5 cycles, GOx-aZIF retained ~80% of activity while less than 2% of activity was retained for GOx-ZIF-8 due to its very low initial activity.

Supplementary Note 11

To determine whether GOx was released during the activity test, RITC labelled GOx instead of GOx was used in the synthesis of GOx-aZIF. The release of GOx from GOx-aZIF thus can be quantified by measuring the fluorescence intensity in the supernatant after activity test. In addition, the activity of released GOx in the supernatant from GOx-aZIF was measured and compared with the total activity of GOx-aZIF. Experiments were carried out in triplicates.

After the activity test, the solution was centrifugated, and the supernatant was collected to measure the fluorescence intensity of GOx-RITC. Results (Supplementary Figure 40) showed that only 8.1% of GOx was released during the activity test. The activity of released GOx was 6.7% compared with the total activity of GOx-aZIF. These experiments confirmed that only a small amount of enzyme was released from aZIF during the activity test.

Supplementary Note 12

To prove that almost no GOx was adsorbed on the surface of aZIF, a control sample of adsorbing GOx on the as-synthesized aZIF (denoted as GOx@aZIF) was prepared. The GOx aqueous solution (80 μL, 5 mg mL^{-1}) was incubated with aZIF suspension (2 mL, 2 mg mL^{-1}) for 30min, followed by the same washing procedure as GOx-aZIF. The amount of GOx adsorbed on the surface of aZIF for the sample of GOx@aZIF was determined as ca. 250 μg. Subsequently, GOx@aZIF was resuspended in aqueous solution containing trypsin (10 mg mL^{-1}) at 40 °C for 3 hours. As comparison, GOx-aZIF was incubated at the same condition with trypsin. The enzyme activity was tested for each sample. Three independent samples were prepared for each condition. The results (Supplementary Figure 45) showed that GOx@aZIF lost ca. 60% of its activity, while no activity loss of GOx-aZIF was observed. In addition, free GOx lost 80% of its activity (Figure 2m). Compared with GOx@aZIF and GOx, the high activity retention of GOx-aZIF demonstrated that GOx was majorly encapsulated inside aZIF instead of being adsorbed on the surface of aZIF.

Supplementary Note 13

Free GOx were majorly co-localized with the lysosomes as indicated by the overlapped yellow spots. Together with the in vitro result that the activity of free GOx sharply decreased upon trypsin digestion (Supplementary Figure 46), the exposure of free GOx in acid degradation environment (e.g. pH 5.0 in cell lysosomes) further excluded the feasibility of using free GOx in glucose detection in living cells. In contrast, most of the GOx-ZIF-8 and GOx-aZIF nanocomposites escaped from the lysosomal degradation owing to the proton sponge effect of the ZIFs.

Supplementary Note 14

For different cell types (4T1, MCF-7, MGC803, and L02), the time is 50 min, 75 min, 85 min, and 90 min, respectively. Supplementary Figure 50 showed corresponding kinetic curves for different cell types. We performed duplicate wells for each cell type, and five different fields in one well were imaged for quantification analysis. Therefore, every point in each curve in Supplementary Figure 50 represents the mean FI value that was calculated from the images of a total of ten different fields. The given graph for each type of cell in Figure 3h was a representative image in one filed.

Supplementary Note 15

Reproducibility of the synthesis was tested for at least 10 batches. The morphologies of products from different batches synthesized by different researchers in our lab were given in Supplementary Figure 51, which showed almost no difference.

Also, the GOx-aZIF sample from 20 different batches were subjected for activity assays. As shown in Supplementary Figure 52, the slope k of enzymatic reactions of different samples was 0.9123 ± 0.0435 (Δabs_{415} min^{-1}) with a coefficient of variation (c_v) of 4.8%, confirming the good reproducibility of enzyme activity for different batches. The homogenous size of GOx-aZIF and the spherical shape under SEM images also demonstrated the good reproducibility of synthesis procedure (Supplementary Figure 53). Besides, no enzymatic activity was detected by UV-Vis spectrophotometer and no protein was detected using Coomassie Bright Blue assay in the supernatant after synthesis for each batch, demonstrating the same encapsulation ratio (~100%) for different batches.

Supplementary References

1. Park, K.S., *et al.* Exceptional chemical and thermal stability of zeolitic imidazolate frameworks. *Proc. Natl. Acad. Sci.* **103**, 10186-10191 (2006).
2. Coverdale, J.P.C., *et al.* Asymmetric transfer hydrogenation by synthetic catalysts in cancer cells. *Nat. Chem.* **10**, 347-354 (2018).
3. Nascimento, R.A.S., *et al.* Single Cell "Glucose Nanosensor" Verifies Elevated Glucose Levels in Individual Cancer Cells. *Nano Lett.* **16**, 1194-1200 (2016).
4. Gambhir, S.S. Molecular imaging of cancer with positron emission tomography. *Nat Rev Cancer* **2**, 683 (2002).
5. Banerjee, S., *et al.* Diagnosis of prostate cancer by desorption electrospray ionization mass spectrometric imaging of small metabolites and lipids. *Proc. Natl. Acad. Sci.* **114**, 3334-3339 (2017).
6. Li, X., *et al.* Electron counting and beam-induced motion correction enable near-atomic-resolution single-particle cryo-EM. *Nat. Methods* **10**, 584 (2013).
7. Rohou, A. & Grigorieff, N. CTFFIND4: Fast and accurate defocus estimation from electron micrographs. *J. Struct. Biol.* **192**, 216-221 (2015).
8. Kremer, J.R., Mastronarde, D.N. & McIntosh, J.R. Computer Visualization of Three-Dimensional Image Data Using IMOD. *J. Struct. Biol.* **116**, 71-76 (1996).
9. Pettersen, E.F., *et al.* UCSF Chimera-A visualization system for exploratory research and analysis. *J. Comput. Chem.* **25**, 1605-1612 (2004).
10. Hammersley, A.P., Svensson, S.O., Hanfland, M., Fitch, A.N. & Hausermann, D. Two-dimensional detector software: From real detector to idealised image or two-theta scan. *High Pressure Res.* **14**, 235-248 (1996).
11. Juhas, P., Davis, T., Farrow, C.L. & Billinge, S.J.L. PDFgetX3: a rapid and highly automatable program for processing powder diffraction data into total scattering pair distribution functions. *J. Appl. Crystallogr.* **46**, 560-566 (2013).
12. Berendsen, H.J.C., van der Spoel, D. & van Drunen, R. GROMACS: A message-passing parallel molecular dynamics implementation. *Comput. Phys. Commun.* **91**, 43-56 (1995).
13. Mackerell Jr., A.D. Empirical force fields for biological macromolecules: Overview and issues. *J. Comput. Chem.* **25**, 1584-1604 (2004).
14. Sayle, R.A. & Milner-White, E.J. RASMOL: biomolecular graphics for all. *Trends Biochem. Sci.* **20**, 374-376 (1995).
15. Cao, S., Bennett, T.D., Keen, D.A., Goodwin, A.L. & Cheetham, A.K. Amorphization of the prototypical zeolitic imidazolate framework ZIF-8 by ball-milling. *Chem. Commun.* **48**, 7805-7807 (2012).
16. Krishna, R. & van Baten, J.M. An investigation of the characteristics of Maxwell–Stefan diffusivities of binary mixtures in silica nanopores. *Chem. Eng. Sci.* **64**, 870-882 (2009).
17. Krishna, R. & van Baten, J.M. Unified Maxwell–Stefan description of binary mixture diffusion in micro- and meso-porous materials. *Chem. Eng. Sci.* **64**, 3159-3178 (2009).
