# Supplementary material for: Packaging and delivering enzymes by amorphous metal-organic frameworks
Source: Nat Commun. 2019 Nov 14;10:5165. doi: 10.1038/s41467-019-13153-x (PMC6856190; doi:10.1038/s41467-019-13153-x)
Supplement: Supplementary file 2 — Description of Additional Supplementary Files [file 41467_2019_13153_MOESM2_ESM.pdf]

Description of Additional Supplementary Files

File name: Supplementary Movie 1

Description: Representative 3D images stack from a single GOx-aZIF nanocomposite.

File name: Supplementary Movie 2

Description: Zoomed images of cross section of a single GOx-aZIF nanocomposite showing pore evolution.

File name: Supplementary Movie 3

Description: Kinetic fluorescence intensity of DCFH-DA cell-staining images in MCF-7 during 4 hour incubation with GOx-aZIF nanocomposite.

File name: Supplementary Movie 4

Description: Kinetic fluorescence intensity showing DCFH-DA cell-staining images in MCF-7 during 4 hour incubation with GOx-ZIF-8 composite.

File name: Supplementary Movie 5

Description: Kinetic fluorescence intensity of DCFH-DA cell-staining images in non-cancer cells (L02) and cancer cells (HepG2) during incubation with GOx-aZIF for 4 hours.
