# Supplementary material for: Packaging and delivering enzymes by amorphous metal-organic frameworks
Source: Nat Commun. 2019 Nov 14;10:5165. doi: 10.1038/s41467-019-13153-x (PMC6856190; doi:10.1038/s41467-019-13153-x)
Supplement: Supplementary file 8 — Reporting Summary [file 41467_2019_13153_MOESM8_ESM.pdf]

nature research

Corresponding author(s): Jun Ge, Wei Wei, Lin Zhang, Sai Li

Last updated by author(s): Oct 10, 2019

Reporting Summary

Nature Research wishes to improve the reproducibility of the work that we publish. This form provides structure for consistency and transparency in reporting. For further information on Nature Research policies, see Authors & Referees and the Editorial Policy Checklist .

Statistics

For all statistical analyses, confirm that the following items are present in the figure legend, table legend, main text, or Methods section.

n/a	Confirmed	
☐	☒	The exact sample size (*n*) for each experimental group/condition, given as a discrete number and unit of measurement
☐	☒	A statement on whether measurements were taken from distinct samples or whether the same sample was measured repeatedly
☐	☒	The statistical test(s) used AND whether they are one- or two-sided<br>*Only common tests should be described solely by name; describe more complex techniques in the Methods section.*
☒	☐	A description of all covariates tested
☒	☐	A description of any assumptions or corrections, such as tests of normality and adjustment for multiple comparisons
☐	☒	A full description of the statistical parameters including central tendency (e.g. means) or other basic estimates (e.g. regression coefficient) AND variation (e.g. standard deviation) or associated estimates of uncertainty (e.g. confidence intervals)
☐	☒	For null hypothesis testing, the test statistic (e.g. *F*, *t*, *r*) with confidence intervals, effect sizes, degrees of freedom and *P* value noted<br>*Give P values as exact values whenever suitable.*
☒	☐	For Bayesian analysis, information on the choice of priors and Markov chain Monte Carlo settings
☒	☐	For hierarchical and complex designs, identification of the appropriate level for tests and full reporting of outcomes
☒	☐	Estimates of effect sizes (e.g. Cohen's *d*, Pearson's *r*), indicating how they were calculated

Our web collection on statistics for biologists contains articles on many of the points above.

Software and code

Policy information about availability of computer code

Data collection	Gromacs 5.1.4, PRODRG 2, Matlab (2015b)
Data analysis	Rasmol 2.7.2.1.1, custom code written in C

For manuscripts utilizing custom algorithms or software that are central to the research but not yet described in published literature, software must be made available to editors/reviewers. We strongly encourage code deposition in a community repository (e.g. GitHub). See the Nature Research guidelines for submitting code & software for further information.

Data

Policy information about availability of data

All manuscripts must include a data availability statement. This statement should provide the following information, where applicable:

- Accession codes, unique identifiers, or web links for publicly available datasets
- A list of figures that have associated raw data
- A description of any restrictions on data availability

Data supporting the findings of this work are available within the paper and its Supplementary Information files. The datasets generated and analyzed during the current study are available from the corresponding author upon request. The source data for all figures and supplementary figures in this paper are provided as Source Data files.

Field-specific reporting

Please select the one below that is the best fit for your research. If you are not sure, read the appropriate sections before making your selection.

Life sciences study design

All studies must disclose on these points even when the disclosure is negative.

Sample size	Cells density was determined by using a Scepyter Handheld Automated Cell Counter, and 50000 cells per well were seeded in the 96-well plates for the in vitro test. Once determined by a High content screening system (HCS), an image analysis software "Harmony"was also helping to analyze the cell number and morphology.
Data exclusions	Do data were exluded.
Replication	All attempts at replication were successful.
Randomization	The allocation of the samples was random.
Blinding	The investigators were blinded to group allocation during data collections.

Reporting for specific materials, systems and methods

We require information from authors about some types of materials, experimental systems and methods used in many studies. Here, indicate whether each material, system or method listed is relevant to your study. If you are not sure if a list item applies to your research, read the appropriate section before selecting a response.

Materials & experimental systems

n/a	Involved in the study
☒	☐ Antibodies
☐	☒ Eukaryotic cell lines
☒	☐ Palaeontology
☒	☐ Animals and other organisms
☒	☐ Human research participants
☒	☐ Clinical data

Methods

n/a	Involved in the study
☒	☐ ChIP-seq
☐	☒ Flow cytometry
☒	☐ MRI-based neuroimaging

Eukaryotic cell lines

Policy information about cell lines

Cell line source(s)	MCF-7 and 4T1 were from the ATCC (American Type Culture Collection). MGC803 was from the China Infrastructure of Cell Line Resource. L02 was from the Cell bank of Type Culture Collection of the Chinese Academy of Sciences.
Authentication	MGC803 and L02 was officially authenticated by using the Short Tandem Repeat method. The other cell lines were not authenticated.
Mycoplasma contamination	We confirm that all cell lines tested negative for mycoplasma contamination.
Commonly misidentified lines (See ICLAC register)	No commonly misidentified lines are occupied.

Flow Cytometry

Plots

Confirm that:

☒ The axis labels state the marker and fluorochrome used (e.g. CD4-FITC).

☒ The axis scales are clearly visible. Include numbers along axes only for bottom left plot of group (a 'group' is an analysis of identical markers).

☒ All plots are contour plots with outliers or pseudocolor plots.

☒ A numerical value for number of cells or percentage (with statistics) is provided.

Methodology

Sample preparation	Flow cytometry was utilized to determine the fluorescent intensity of different nano-particles. The particles were prepared as described in Supporting Information. Before conducting the flow cytometry, particles were resuspended, sent to supersonic and then filtered by 400-mesh sieve.

Instrument	CytoFLEX LX Flow Cytometer
Software	CytExpert 2.1
Cell population abundance	The collection of particles didn't stop until the number of the nano-particles ranging from 100-300nm reaches 10000, which assured particle abundance.
Gating strategy	The gate was set according to the particle size.

☒ Tick this box to confirm that a figure exemplifying the gating strategy is provided in the Supplementary Information.
